# Nucleic acid binding by SAMHD1 contributes to the antiretroviral activity and is enhanced by the GpsN modification

**DOI:** 10.1038/s41467-021-21023-8

**Published:** 2021-02-02

**Authors:** Corey H. Yu, Akash Bhattacharya, Mirjana Persaud, Alexander B. Taylor, Zhonghua Wang, Angel Bulnes-Ramos, Joella Xu, Anastasia Selyutina, Alicia Martinez-Lopez, Kristin Cano, Borries Demeler, Baek Kim, Stephen C. Hardies, Felipe Diaz-Griffero, Dmitri N. Ivanov

**Affiliations:** 1 Department of Biochemistry and Structural Biology, UT Health San Antonio, San Antonio, TX USA; 2 Department of Microbiology and Immunology, Albert Einstein College of Medicine, Bronx, NY USA; 3 Department of Pediatrics, Emory School of Medicine, Atlanta, GA USA; 4 Department of Chemistry and Biochemistry, University of Lethbridge, Lethbridge, AB Canada; 5 Department of Chemistry and Biochemistry, University of Montana, Missoula, MT USA

**Keywords:** X-ray crystallography, Structural biology, Medical research

## Abstract

SAMHD1 impedes infection of myeloid cells and resting T lymphocytes by retroviruses, and the enzymatic activity of the protein—dephosphorylation of deoxynucleotide triphosphates (dNTPs)—implicates enzymatic dNTP depletion in innate antiviral immunity. Here we show that the allosteric binding sites of the enzyme are plastic and can accommodate oligonucleotides in place of the allosteric activators, GTP and dNTP. SAMHD1 displays a preference for oligonucleotides containing phosphorothioate bonds in the Rp configuration located 3’ to G nucleotides (GpsN), the modification pattern that occurs in a mechanism of antiviral defense in prokaryotes. In the presence of GTP and dNTPs, binding of GpsN-containing oligonucleotides promotes formation of a distinct tetramer with mixed occupancy of the allosteric sites. Mutations that impair formation of the mixed-occupancy complex abolish the antiretroviral activity of SAMHD1, but not its ability to deplete dNTPs. The findings link nucleic acid binding to the antiretroviral activity of SAMHD1, shed light on the immunomodulatory effects of synthetic phosphorothioated oligonucleotides and raise questions about the role of nucleic acid phosphorothioation in human innate immunity.

## Abstract (web summary)

SAMHD1 catalyses the dephosphorylation of deoxynucleotide triphosphates (dNTPs) and has antiretroviral activity. Here, the authors present the crystal structures of SAMHD1-oligonucleotide complexes, which reveal that the allosteric binding sites of SAMHD1 are plastic and can fit oligonucleotides in place of the two allosteric activators GTP and dNTP, and they also show that SAMHD1 recognises GpsN phosphorothioation modifications in nucleic acids, which is of interest in drug design.

## Introduction

Identification of SAMHD1 as the factor that impedes infection of myeloid cells by HIV and SIV brought to light an intrinsic cellular antiviral immunity mechanism that relies on controlled reduction of deoxynucleotide triphosphate (dNTP) availability^1–4^. Hydrolysis of dNTPs to unphosphorylated nucleosides and inorganic triphosphate catalyzed by SAMHD1—activity believed to be the main mechanism of enzymatic dNTP depletion in cells—correlates with the impaired ability of lentiviruses to undergo reverse transcription in myeloid and resting T cells and is thought to mediate the antiviral function of the protein^3–10^. To counter this infectivity block, HIV-2 and SIV viruses encode an auxiliary protein Vpx, which targets SAMHD1 for proteasomal degradation and facilitates infection of noncycling immune cells by these viruses^1,2,5,6,11,12^.

Antiviral defense mechanisms that depend on controlled depletion of dNTPs may also exist in prokaryotes. Ribonucleotide reductase and other enzymes of dNTP biosynthesis are frequently encoded in bacteriophage genomes, an indication that inadequate dNTP supply is a common problem encountered by bacteriophages^13,14^. Deoxynucleotide hydrolases of the same HD-domain superfamily as SAMHD1 are known in prokaryotes^15,16^, and the inhibition of the *E. coli* HD-domain dNTPase by the 1.2 protein of enterobacteria phage T7 evokes depletion of SAMHD1 by Vpx^17^.

Biochemical and structural studies revealed that dNTP hydrolysis by SAMHD1 is allosterically activated by binding of GTP and dNTP at the two adjacent allosteric binding sites A1 and A2 and the concomitant oligomerization of the protein into the catalytically active tetramer^18–22^. This substrate-activation mechanism may help establish stable equilibrium concentrations of dNTPs by making the protein more active at higher dNTP concentrations, but its contribution to the controlled dNTP depletion in noncycling cells is less clear. Several tetramerization-impaired mutants of SAMHD1 that display a pronounced dNTPase defect in vitro can nevertheless deplete dNTPs in cells and restrict retroviral replication, suggesting that an alternative or additional mechanism may contribute to the dNTPase activation of SAMHD1 in restrictive cells^23,24^.

Cellular control of SAMHD1 activity remains poorly understood. It is well established that phosphorylation of threonine 592 (T592) negatively correlates with HIV restriction by SAMHD1^25–27^, and multiple cellular signals have now been shown to alter T592 phosphorylation^28–34^. Surprisingly, phosphomimetic mutations of T592 abolish the antiretroviral activity, but do not affect dNTP hydrolysis in vitro or in cells, which raises questions about the exact relationship between dNTP depletion and the restriction of retroviral replication^23,25,27^. It is also unclear whether and how the dNTPase activity contributes to other, less well-understood biological functions of SAMHD1, such as its roles in the DNA double-strand break repair^35^, interferon signaling^36–38^, restriction of LINE-1 retroelements^39–41^, degradation of nascent DNA at stalled replication forks^42^, and posttranscriptional control of mRNAs in regulatory T cells^43^. Involvement of nucleic acids in these distinct cellular activities suggests that the interaction of SAMHD1 with nucleic acids^44–49^ may contribute to its function.

In this study, we show that the allosteric binding sites of the enzyme are plastic and can accommodate oligonucleotides in place of the allosteric activators, GTP and dNTP. Intriguingly, we observe that nucleic acid binding by SAMHD1 is enhanced by phosphorothioate linkages of Rp stereochemistry located 3′ to guanine nucleotides in nucleic acids. The phosphorothioation pattern recognized by SAMHD1, GpsN, matches modifications generated by the Dnd gene cluster in the enzymatic DNA phosphorothioation in bacteria and archea^50–52^ that are widespread in the human microbiome^53^ and the GpsG linkages recently detected in human RNA^54^, which suggests that the immune function of SAMHD1 in humans may have something in common with the unusual phosphorothioation-dependent mechanism of antiviral defense in prokaryotes.

The findings also shed light on the immunomodulatory effects of synthetic phosphorothioated oligonucleotides in mammalian cells. Phosphorothioation enhances nuclease resistance and cell permeability of synthetic oligonucleotides and is widely used in the emerging field of oligonucleotide therapeutics. One of the challenges in the clinical development of phosphorothioated oligonucleotides is posed by the well-known but poorly understood immunological or cytotoxic side effects associated with this modification^55,56^. SAMHD1 has recently been identified as the cellular factor whose direct interaction with short synthetic phosphorothioated oligonucleotides facilitates expansion of engineered regulatory T cells ex vivo^43,57^. In this study, we show how the stereochemistry and the location of phosphorothioate bonds can enhance binding of oligonucleotides to SAMHD1, and possibly other immune factors, opening a path for structure-guided optimization of these promising bioactive and therapeutic agents.

Collectively, our results shed new light onto the intricate allosteric regulation of SAMHD1 activity and raise questions about the role of nucleic acid phosphorothioation in dNTP metabolism and antiviral immunity in bacteria and in humans.

## Results

### Binding of short oligonucleotides to SAMHD1 is enhanced by phosphorothioation and is coupled to protein dimerization

**Fig. 1:**
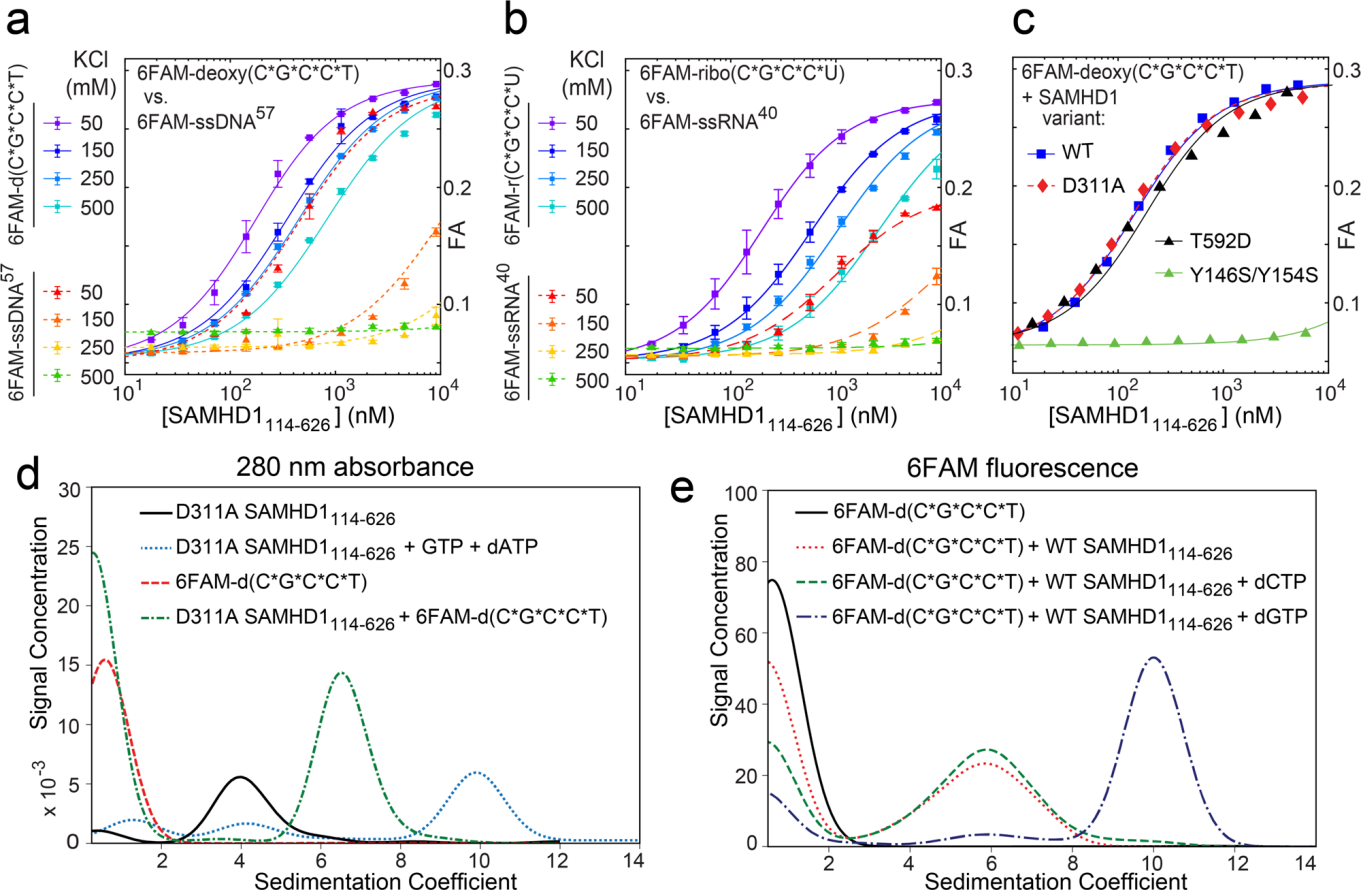
Interaction of SAMHD1 with short phosphorothioated oligonucleotides. **a**, **b** Fluorescence anisotropy (FA)-binding assays reveal that binding of short phosphorothioated oligonucleotides to SAMHD1 is remarkably tolerant to increasing salt concentration compared to phosphodiester-linked (6FAM-ssDNA^57^ and 6FAM-ssRNA^40^) oligonucleotide binding^47^. Location of phosphorothioate bonds is denoted with asterisks in all figures (*n* = 2 independent experiments). **c** Oligomerization-defective mutant Y146S/Y154S is impaired in oligonucleotide binding. **d** Analysis of SAMHD1 ligand-induced oligomerization by analytical ultracentrifugation (AUC) using 280-nm absorbance readout. **e** AUC analysis using 6FAM fluorescence detection reveals association of oligonucleotides with SAMHD1 dimers and tetramers. Error bars represent s.d. of two replicate measurements performed on distinct samples. Source data are provided as a Source Data File.

The role of nucleic acid binding in the biological function of SAMHD1 remains elusive because nucleic acid-binding sites are not well-defined and key interacting residues have not been identified^44–49^. In a survey of oligonucleotide binding by SAMHD1, we observed that short phosphorothioated oligonucleotides bind to SAMHD1 with affinities comparable to affinities of the longer RNA and DNA oligonucleotides investigated previously^47^ (Fig. 1a, b) (asterisks are used to denote location of phosphorothioated linkages in oligonucleotides). Notably, whereas binding of single-stranded phosphodiester-linked oligonucleotides was strongly attenuated by increasing ionic strength of the buffer, binding affinities of phosphorothioate-containing oligonucleotides were much less sensitive to higher salt concentrations. The SAM domain of SAMHD1 is dispensable for binding of short oligonucleotides (Supplementary Fig. 1A) and the HD-domain constructs (SAMHD1_114–626_) were used throughout this study, unless otherwise noted, because neither the dNTPase activity (Supplementary Fig. 1B) nor the retroviral restriction activity of SAMHD1^44^ is affected by SAM deletion, and the SAMHD1_114–626_ HD-domain construct is less prone to aggregation in vitro. We also monitored nuclease activities of our SAMHD1 protein preps to ensure that the activity of copurifying nucleases was sufficiently low not to affect any of the experiments described in this study (Supplementary Figs. 1C, D and 2D).

To further evaluate the specificity of the interaction, we investigated whether the binding is affected by point mutations known to perturb SAMHD1 function. D311A, the mutation that disrupts binding of the catalytic metal in the active site, and the phosphomimetic mutation T592D had no effect on the binding^25^. In contrast, the oligomerization-defective mutant Y146S/Y154S^24^ impaired the interaction, which suggested that oligonucleotides bind at a specific site formed upon SAMHD1 oligomerization (Fig. 1c).

The relationship between oligonucleotide binding and SAMHD1 oligomerization was then investigated by analytical ultracentrifugation (AUC). AUC experiments were performed with the 6-carboxyfluoroscein (6FAM)-labeled oligonucleotides used in the binding assays, and sedimentation was monitored by either absorbance at 280 nm (Fig. 1d) or by fluorescence detection (Fig. 1e). First, we analyzed the D311A SAMHD1_114–626_ variant using 280-nm absorbance (Fig. 1d). The D311A mutation has no effect on tetramerization, but it abolishes the dNTPase activity and prevents dNTP depletion during the sedimentation experiment^23^. In agreement with previous studies^23,24,58,59^, we observed that upon addition of GTP and dATP to SAMHD1, the protein tetramerizes and sediments at S ≈ 10. In contrast, addition of the 6FAM-d(C*G*C*C*T) oligonucleotide to D311A SAMHD1_114–626_ promotes formation of a distinct species at S ≈ 6, which is close to the predicted sedimentation coefficient of the SAMHD1 dimer.

Fluorescence-detection experiments revealed that in the absence of nucleotide triphosphates, protein-bound oligonucleotides sediment at the sedimentation coefficient of the SAMHD1 dimer and not the monomer (Fig. 1e). We then investigated the effect of dNTP addition on oligonucleotide sedimentation. The protein was first incubated for 3 min with the oligonucleotide and 50 μM of dNTP was then added prior to AUC analysis. Addition of dCTP to the sample had no effect on the sedimentation of SAMHD1-bound oligonucleotides, whereas in the presence of dGTP, which can occupy both A1 and A2 allosteric sites and promotes SAMHD1 tetramerization, most of the protein-bound 6FAM-d(C*G*C*C*T) sedimented as the SAMHD1 tetramer.

Collectively, the experiments reveal that oligonucleotide binding to SAMHD1 is coupled to protein dimerization, in agreement with previously published results^47^. Furthermore, oligonucleotides can also associate with SAMHD1 tetramers formed in the presence of nucleotide triphosphates.

### Oligonucleotides bind at the allosteric sites, but do not promote SAMHD1 tetramerization or the dNTPase activity in the absence of allosteric activators, GTP and dNTP

**Fig. 2:**
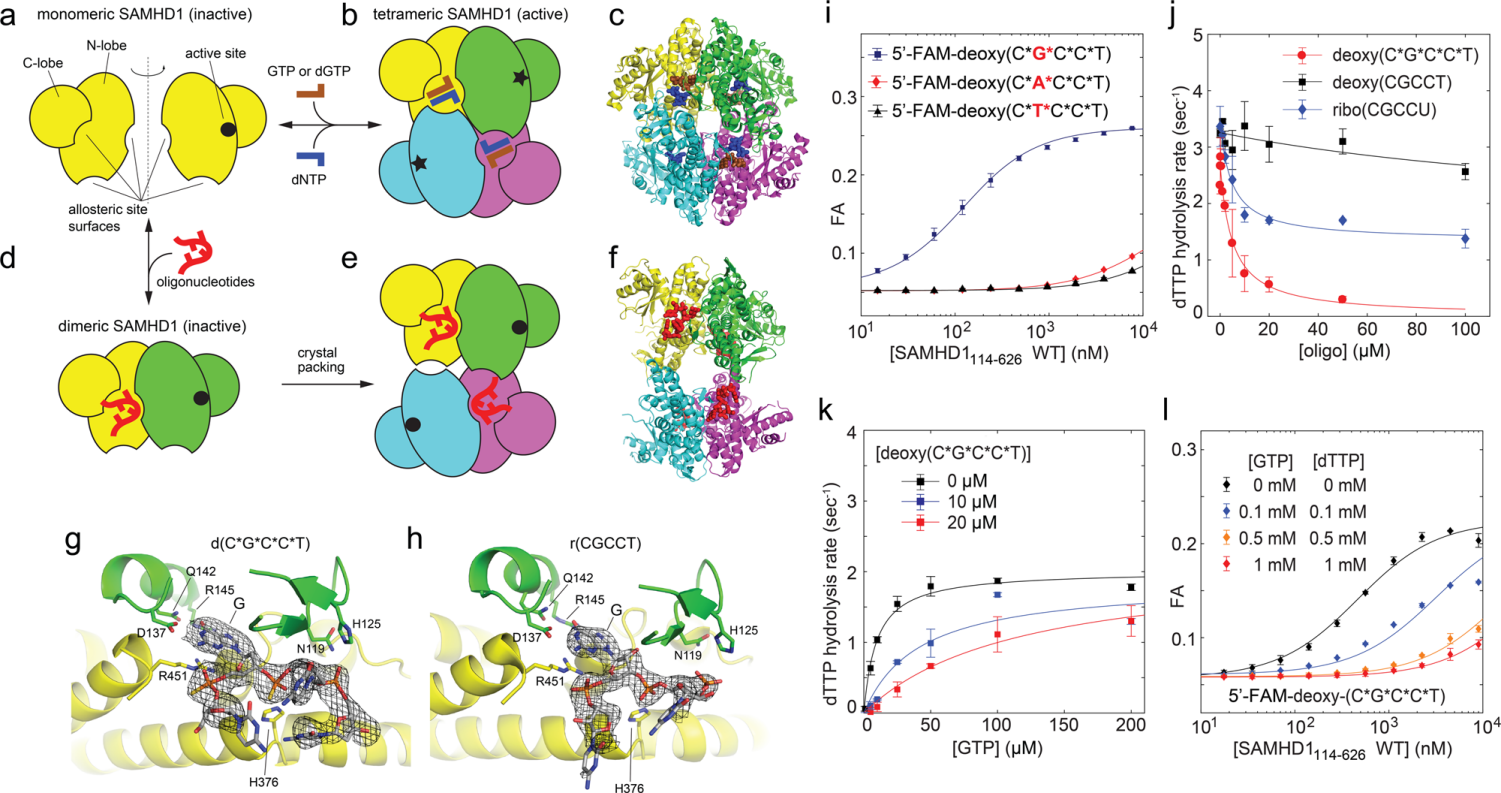
Crystal structures of SAMHD1:oligonucleotide complexes reveal that oligonucleotides compete with nucleotide triphosphates for binding to the allosteric sites. **a** Without allosteric ligands, the HD-domain construct of SAMHD1 is monomeric in solution. **b** In the presence of nucleotide triphosphates, it assembles into a tetramer with almost perfect 222-point symmetry. **c** Backbone cartoon of the GTP/dCTP-bound SAMHD1 structure^18^ (PDB: 4TNP). **d** In the presence of oligonucleotides SAMHD1 dimerizes. **e** Packing of two SAMHD1 dimers in the asymmetric unit of the oligonucleotide-bound crystals lacks 222-point symmetry. **f** Backbone cartoon of the d(C*G*C*C*T)-bound SAMHD1 structure (PDB: 6U6X). Electron-density maps of the d(C*G*C*C*T) (**g**) and r(CGCCU) (**h**) oligonucleotides bound in the allosteric sites of SAMHD1 (PDB: 6U6X and PDB: 6U6X, respectively). The mesh shows the composite 2mFo-DFc omit map contoured at 1.5 *σ*. **i** Binding of the G nucleobase in the guanine-recognition pocket of A1 is a determinant of the oligonucleotide- binding affinity (*n* = 2 independent experiments). **j** Phosphorothioation enhances dNTPase inhibition by oligonucleotide binding. dTTP hydrolysis rates were measured at [GTP] = 50 μM and [dTTP]=1 mM (*n* = 2 independent experiments). **k** *EC*_*50*_^*GTP*^ but not *k*_*cat*_ of the dNTPase activity is affected by d(C*G*C*C*T) binding, which is consistent with competition between d(C*G*C*C*T) and GTP for the allosteric sites (*n* = 2 independent experiments). **l** Increasing GTP concentrations attenuate binding of 6FAM-d(C*G*C*C*T) to SAMHD1 as monitored by fluorescence anisotropy (*n* = 2 independent experiments). Error bars represent s.d. of two replicate measurements performed on distinct samples. Source data are provided as a Source Data File.

To gain further mechanistic insight into the interaction of SAMHD1 with nucleic acids, we determined structures of SAMHD1_114–626_ bound to the phosphorothioate-linked deoxy(C*G*C*C*T) and the phosphodiester-linked ribo(CGCCU) oligonucleotides by X-ray crystallography (Table 1). Similar to other crystallographic studies of SAMHD1, all of the crystals analyzed in this study contained four SAMHD1 monomers within the asymmetric unit, which should not be confused with the oligomerization state of the protein. It is generally accepted that the catalytically active state of SAMHD1 is a tightly packed tetramer with almost perfect 222-point symmetry, first observed in the dGTP-bound structure of the protein^18^, which forms upon binding of allosteric nucleotide-triphosphate activators at two adjacent sites, A1 and A2 (Fig. 2a–c). This tetramer can be described as a dimer of dimers, with one major interface (dimer-1 interface) mediating SAMHD1 dimerization and another interface (dimer-2 or tetramer interface) bringing two SAMHD1 dimers together into a tetramer. In contrast, in our oligonucleotide-bound structures, only the dimer-1 interface is intact, but the packing of the two SAMHD1 dimers in the asymmetric unit is looser, the dimer-2 (tetramer) interface is not properly formed, and no 222-point symmetry is present. We conclude that in our crystals, the content of the asymmetric unit represents crystal packing of two oligonucleotide-bound SAMHD1 dimers (Fig. 2d–f). This is in agreement with our AUC data, which revealed coupling between oligonucleotide binding and SAMHD1 dimerization, and with previous studies by others, which suggested that oligonucleotide binding traps SAMHD1 in the inactive dimeric state^47^.

**Table 1:** Data collection and refinement statistics.

	ribo(CGCCU) complex	deoxy(C*G*C*C*T) complex	deoxy(TG*TTCA) complex	
Data collection				
PDB code	6U6Y	6U6X	6U6Z	
X-ray source	APS NE-CAT ID-E	APS NE-CAT ID-E	APS NE-CAT ID-C	APS NE-CAT ID-C
Space group	*P*2_1_	*P*1	*P*1	*P*1
Cell dimensions				
*a*, *b*, *c* (Å)	77.3, 183.6, 81.6	69.4, 81.9, 106.0	81.6, 94.7, 96.3	81.7, 94.7, 96.5
*α, β, γ* (°)	90, 100.9, 90	69.7, 76.3, 82.8	72.9, 70.6, 65.0	72.9, 71.0, 65.0
Wavelength (Å)	0.97919	0.97923	0.97910	1.77120
Resolution (Å)	75.96–2.47	61.66–2.58	68.27–2.10	63.89–2.24
	(2.53–2.47)^a^	(2.65–2.58)	(2.15–2.10)	(2.30–2.24)
*R*_merge_	0.113 (1.64)	0.050 (0.971)	0.060 (0.899)	0.087 (1.23)
*R*_pim_	0.065 (0.995)	0.035 (0.684)	0.036 (0.534)	0.034 (0.536)
CC_1/2_	(0.50)	(0.62)	(0.73)	(0.65)
Mean *Ι* / *σΙ*	9.5 (1.2)	12.3 (1.2)	10.5 (1.4)	11.0 (1.3)
Completeness (%)	99.9 (99.7)	97.8 (97.9)	93.0 (93.8)	91.7 (89.5)
Redundancy	3.8 (3.7)	2.9 (3.0)	3.7 (3.7)	7.2 (7.0)
Wilson value (Å^2^)	43.0	67.3	48.8	51.7
Refinement				
Resolution (Å)	75.96–2.47	61.22–2.58	68.27–2.10	
No. reflections	79,670	65,286	131,790	
*R*_work_/*R*_free_	0.180/0.236	0.196/0.237	0.173/0.206	
No. atoms				
Protein	14,173	13,242	14,134	
Nucleic acid	285	273	184	
Zn^2+^	4	4	4	
Solvent	209	12	294	
B-factors (Å^2^)				
Protein	62.3	98.9	64.4	
Nucleic acid	100.1	117.5	89.0	
Zn^2+^	57.6	110.6	45.2	
Solvent	47.2	68.1	52.6	
R.m.s deviations				
Bond lengths (Å)	0.009	0.017	0.009	
Bond angles (°)	0.969	1.193	0.909	
Ramachandran plot				
Favored (%)	98.0	96.9	98.1	
Allowed (%)	1.9	3.0	1.8	
Outliers (%)	0.1	0.1	0.1	

^a^Values in parentheses are for the highest-resolution shell.

The structures revealed that bound oligonucleotides span both allosteric binding sites A1 and A2 and engage many of the same residues that mediate binding of nucleotide triphosphates (Fig. 2g, h). For example, the guanine nucleobase of the sole G nucleotide within the oligos is accommodated in the guanine-recognition pocket of the A1 formed by SAMHD1 residues D137, Q142, R145, and R451. The most notable contact with the oligonucleotide backbone is formed by the residue H376 that we discuss below in more detail. The key difference between the oligonucleotide-bound and nucleotide-triphosphate-bound structures is that in the nucleotide-triphosphate-bound tetramer, the allosteric ligands in the A1 and A2 sites make contacts with residues of three distinct monomers, whereas in our oligonucleotide-bound structures, residues of only two monomers are involved (Fig. 2b, e).

The oligonucleotide-bound structures explain the inhibition of dNTPase activity by oligonucleotide binding^44,47^. Oligonucleotides compete with nucleotide triphosphates for the allosteric sites, but, in the absence of GTP and dNTPs, the oligonucleotide-bound SAMHD1 dimers are not capable of assembling into the catalytically active tetramer. To further test this, we first investigated whether binding of the G nucleobase in the guanine-recognition pocket of the A1 site is one of the requirements for high-affinity interaction with short oligonucleotides. Indeed, fluorescence-polarization binding experiments revealed that substitution of the G nucleotide with A or T abolished the binding of the 6FAM-labeled oligonucleotides to SAMHD1 (Fig. 2i).

We then looked at the dNTPase activity of SAMHD1 in the presence of oligonucleotides using the NMR-based dNTPase assay, which revealed that incubation with phosphorothioated oligonucleotides resulted in the most potent inhibition of dNTPase activity (Fig. 2j). To confirm that inhibition of dNTPase activity was due to competition of oligonucleotides with nucleotide triphosphates for the allosteric sites, we measured dNTP hydrolysis rates as a function of GTP concentration in the presence of two different concentrations of d(C*G*C*C*T). The data are consistent with competition between GTP and oligonucleotides for the allosteric sites: the apparent *EC*_*50*_^*GTP*^ of the enzyme was higher at higher concentration of oligonucleotide, but the maximal hydrolysis rate (*k*_*cat*_) was not affected (Fig. 2k). We also investigated competition between GTP and 6FAM-d(C*G*C*C*T) in the fluorescence anisotropy-binding experiments. We observed that the apparent *K*_*d*_ of oligonucleotide binding was increased at higher GTP concentrations as expected for competitive binding of two ligands to the same binding site (Fig. 2l).

Collectively, these data establish that short oligonucleotides used in this study compete with allosteric nucleotide-triphosphate activators for the same binding sites, and, when present at high concentration, inhibit the dNTPase activity of SAMHD1.

### The GpsN modification—a phosphorothioate bond of Rp chirality located 3′ to a G nucleotide—is a determinant of high-affinity nucleic acid binding to SAMHD1

**Fig. 3:**
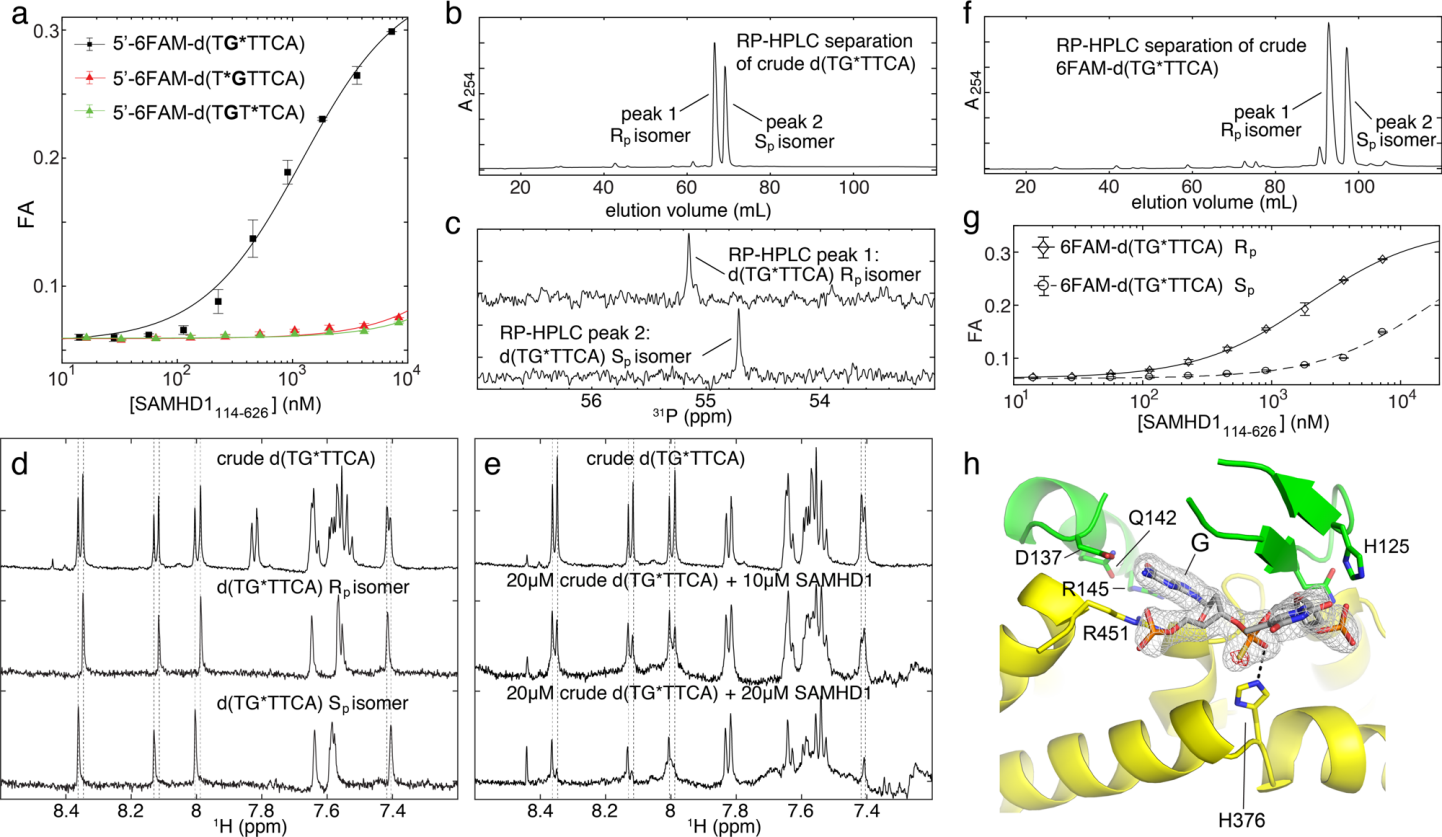
Stereospecificity of the SAMHD1–GpsN binding. **a** Location of the phosphorothioate bond within the oligo is a determinant of SAMHD1-binding affinity (*n* = 2 independent experiments). **b** *Rp* and *Sp* diastereomers can be separated by reverse-phase chromatography. **c** *Sp* and *Rp* diastereomers can be identified using ^31^P NMR^60^. **d** Doublet peaks in the ^1^H NMR spectra can be assigned to *Sp* and *Rp* stereoisomers. **e** Addition of SAMHD1 to crude d(TG*TTCA) oligos selectively broadens NMR signals of the Rp stereoisomer. **f** Purification of 6FAM-labeled *Rp* and *Sp* diastereomers by reverse-phase chromatography. **g** SAMHD1 displays about tenfold higher affinity for the *Rp* stereoisomer of 6FAM-d(TG*TTCA) (*n* = 2 independent experiments). **h** Difference (black) and anomalous difference Fourier (red) diffraction electron-density maps reveal that in the crystals of the SAMHD1:d(TG*TTCA) complex, the bound oligonucleotide is the Rp stereoisomer. The mesh shows the composite 2mFo-DFc omit map contoured at 1.5 *σ*. Error bars represent s.d. of two replicate measurements performed on distinct samples. Source data are provided as a Source Data File.

The structures also offer insight into why phosphorothioation increases oligonucleotide-binding affinity. In the d(C*G*C*C*T)-bound structure, histidine H376 appears to form a hydrogen bond with one of the nonbridging atoms of the phosphorothioate linkage between the G2 and C3 nucleotides in the oligo. A similar contact is observed between H125 and the phosphorothioate bond between C3 and C4. To evaluate the contribution of these interactions to the binding affinity, we first performed binding experiments with 6FAM-labeled d(TGTTCA) oligonucleotides containing single phosphorothioate linkages at distinct locations. These experiments revealed that the phosphorothioate bond following the G nucleotide is the main determinant of SAMHD1 preference for phosphorothioated oligonucleotides (Fig. 3a). Furthermore, there was no significant difference in binding affinity of deoxy(TG*TTCA) and ribo(UG*UUCA) oligos (Supplementary Fig. 2A). The addition of a single GpsN phosphorothioate bond made oligonucleotides significantly more salt-tolerant than nonphosphorothioated oligos (Fig. 1a, b and Supplementary Fig. 2B, C).

We then investigated the stereospecificity of the interaction. DNA phosphorothioation replaces one nonbridging oxygen with sulfur and creates a chiral center at phosphorous. Conventional solid-phase synthesis of phosphorothioated oligonucleotides is not stereoselective and the two stereoisomers, Rp and Sp, can occur with roughly equal probability at each phosphorothioate linkage. Although the resolution of the electron-density map limited our ability to determine positions of sulfur atoms in the phosphorothioate linkages in the d(C*G*C*C*T)-SAMHD1 complex with high confidence, the data indicated that the interaction may be stereoselective. To further explore this, we used NMR to investigate the interaction of SAMHD1 with the d(TG*TTCA) oligonucleotide containing a single phosphorothioate bond after the sole G nucleotide. This oligonucleotide was used because it could be readily separated into pure Rp and Sp diastereomers by reverse-phase chromatography (Fig. 3b). Identity of the two purified stereoisomers was determined using ^31^P NMR as previously described^60^ (Fig. 3c). We could then assign doublets in the ^1^H NMR spectra of the crude d(TG*TTCA) to the two diastereomers of the oligonucleotide (Fig. 3d). When SAMHD1 was titrated into the NMR sample of crude d(TG*TTCA), we observed that ^1^H NMR signals corresponding to the Rp diastereomer of the oligo gradually disappeared, whereas the signals of the Sp diastereomer were affected to a much lesser degree (Fig. 3e). We also prepared stereopure 6FAM-d(TG*TTCA) oligonucleotides (Fig. 3f) and performed fluorescence-polarization-binding experiments to confirm the higher affinity of SAMHD1 for the Rp diastereomer (Fig. 3g). Finally, we cocrystallized SAMHD1 with the crude d(TG*TTCA) oligonucleotide and resolved positions of the sulfur atoms in the crystal using anomalous scattering of sulfur in the monochromatic X-ray beam at a long wavelength (1.7712 Å). In the anomalous electron-density map, location of the sulfur atoms in the SAMHD1/ d(TG*TTCA) complex corresponded to the Rp stereoisomer (Fig. 3h). Furthermore, when the oligonucleotide was modeled without the phosphorothioate modification (e.g., oxygen modeled in place of sulfur), a significant positive difference map peak appeared at the Rp position to indicate the presence of the larger sulfur scatterer. In summary, the recognition of the GpsN modification by SAMHD1 is stereospecific for the Rp conformation of the phosphorothioate linkage.

### In the presence of oligonucleotides, GTP and dNTP, SAMHD1 assembles into mixed-occupancy tetramers

The results described above raise an intriguing question: how do oligonucleotides associate with SAMHD1 tetramers (Fig. 1e) if they compete for the same binding sites with nucleotide triphosphates, whose binding is required for SAMHD1 tetramerization? To gain further insight into this, we investigated oligonucleotide-binding stoichiometry in the SAMHD1-oligonucleotide complexes formed with and without nucleotide triphosphates.

**Fig. 4:**
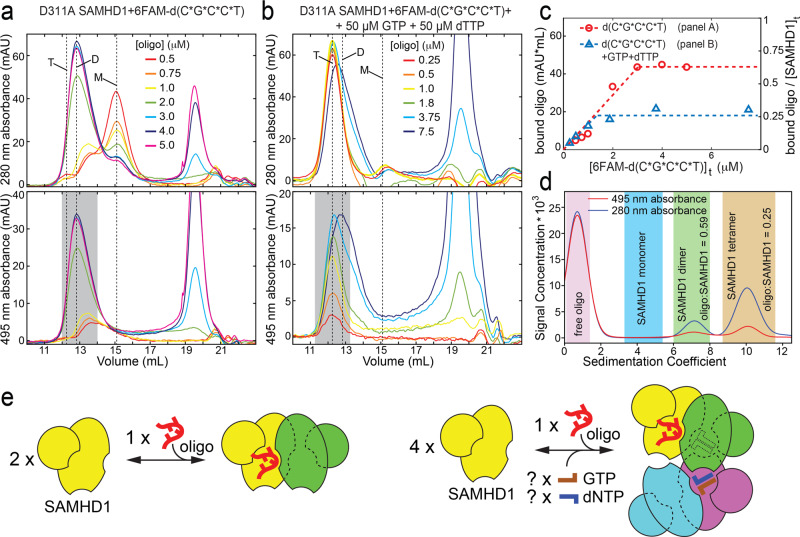
Binding stoichiometry in SAMHD1:oligonucleotide complexes. **a** Size-exclusion chromatography (SEC) analysis of oligonucleotide:D311A SAMHD1 complexes formed with no GTP or dATP present. Absorbance measurements at two wavelengths, 280 and 495 nm, allow independent quantification of SAMHD1 and the oligonucleotide. The amount of oligonucleotide associated with the SAMHD1 dimer was determined by integrating the shaded area of the 495-nm chromatogram. The three dashed lines indicate the retention volumes of SAMHD1 monomer (M), dimer (D), and tetramer (T). **b** The same experiment as in (**a**) but performed in the presence of 50 μM GTP and 50 μM dATP in the running buffer and during incubation with the oligonucleotide. **c** Amounts of D311A SAMHD1-bound oligonucleotide, as determined by integrating the shaded areas of the 495-nm chromatograms in (**a**, **b**), plotted as a function of the total amount of the crude d(C*G*C*C*T) oligonucleotide added to SAMHD1. The left *Y* axis indicates absolute amounts and the right axis shows it as a fraction of the total amount of SAMHD1 (~5 μΜ) used in the titrations. **d** Analytical ultracentrifugation analysis of the d(C*G*C*C*T):D311A SAMHD1 complexes formed in the presence of GTP and dATP using 280- and 495-nm multiwavelength detection. Quantification of SAMHD1 and the oligonucleotide present in distinct complexes was performed by integrating the shaded areas of the plot. **e** Asymmetric oligonucleotide:SAMHD1 complexes formed as a result of the allosteric communication between distinct SAMHD1 monomers in the oligomer.

We first quantified the stoichiometry of oligonucleotide binding to the catalytically inactive D311A SAMHD1 construct by size-exclusion chromatography (SEC) with absorbance monitored at two wavelengths, 280 and 495 nm. The use of multiwavelength detection in these experiments enabled independent quantification of the FAM-labeled oligonucleotides and SAMHD1 present throughout the size-exclusion chromatogram (see “Methods”). A fixed amount of D311A SAMHD1 (~5 μM) was incubated with increasing amounts of oligonucleotides and the complexes were then analyzed by SEC. The first set of titrations was carried out with no nucleotide triphosphates added (Fig. 4a). In the second set of titrations, 50 μM of GTP and 50 μM of dTTP were included during preincubation with the oligonucleotide and also in the SEC running buffer (Fig. 4b). The amount of SAMHD1-bound oligonucleotide was quantified by integrating 495-nm absorbance in the areas shaded in gray in the A_495_ chromatograms. These studies revealed that when no GTP/dTTP were present, the amount of SAMHD1-bound oligonucleotide gradually increased and then saturated at roughly 1:2 ratio to the total SAMHD1 (Fig. 4c). This result is in agreement with the NMR titration experiments, which revealed that complete disappearance of the NMR signals corresponding to the Rp enantiomer occurred when the ratio Rp d(TG*TTCA):SAMHD1 ratio was close to 1:2 (Fig. 3e). Remarkably, in the presence of GTP/dTTP, the oligonucleotide:SAMHD1 ratio saturated at the 1:4 value (Fig. 4c), which suggested that a SAMHD1 tetramer with distinct occupancy of the allosteric sites is formed in the presence of oligonucleotides (see “Methods” for more details).

We then investigated the stoichiometry in the 6FAM-d(C*G*C*C*T):D311A SAMHD1 complexes assembled in the presence of GTP/dATP by AUC. We used the 280- and 495-nm multiwavelength acquisition capability of the Beckman Optima AUC instrument to quantify amounts of SAMHD1 and 6FAM-d(C*G*C*C*T) present in each of the distinct sedimentation species in the sample. In agreement with our fluorescence-detection AUC studies (Fig. 1e) and SEC studies, we observed that 6FAM-d(C*G*C*C*T) was associated with both SAMHD1 dimers (S ≈ 6) and SAMHD1 tetramers (S ≈ 10). Integration of 280- and 495-nm absorbance of the dimer and tetramer yielded 6FAM-d(C*G*C*C*T):SAMHD1 ratio values of 0.59 and 0.25, respectively, which is a very close match with the values we obtained from the SEC experiments (Fig. 4c, d).

Collectively, these data reveal anti-cooperativity of oligonucleotide binding to SAMHD1 and allosteric communication between SAMHD1 subunits within the tetramer. These phenomena facilitate formation of SAMHD1 oligomers with mixed, but well-defined occupancy of the allosteric sites (Fig. 4e). It is notable that although at high concentrations oligonucleotides compete with GTP and dNTP and trap the enzyme in the inactive dimeric state, at substoichiometric amounts, GpsN-containing oligonucleotides promote a distinct allosteric state of the enzyme: a tetramer, in which one A1/A2 site is occupied by the bound oligonucleotide, whereas the remaining allosteric sites are likely occupied by nucleotide triphosphates.

Finally, we sought to determine whether this mixed-occupancy SAMHD1 tetramer is catalytically active. To this end, we repeated SEC-analyzed oligonucleotide titration series using the catalytically active WT SAMHD1 construct (Supplementary Fig. 3). WT SAMHD1 tetramers were prepared in the presence of GTP and dCTP and increasing amounts of 6FAM-d(C*G*C*C*T). The samples were then passed through the size-exclusion column and the dNTPase activity of the protein eluting at 12-mL retention volume was immediately measured using the NMR-based dNTPase assay. In agreement with earlier results, we observed that oligonucleotide binding by the WT SAMHD1 is virtually indistinguishable from that of the D311A variant and similarly saturates at ~1:4 oligo:SAMHD1 ratio in the presence of GTP/dCTP. Notably, the dNTPase activity of WT SAMHD1 tetramer (12-mL retention volume) gradually decreased as the amount of bound oligonucleotide increased, and as the amount of the bound oligonucleotide saturated, the dNTPase activity stabilized at ~20% of the full activity without oligonucleotides present. The data suggest that the oligonucleotide-bound mixed-occupancy tetramer retains some dNTPase activity, but we cannot completely rule out the possibility that the nonzero residual activity results from the re-equilibration between oligonucleotide and GTP/dCTP binding taking place between the SEC run and the dNTPase measurement. A more careful examination of how nucleic acid binding affects dNTPase activity will have to be performed once the physiological nucleic acid ligands of SAMHD1 have been identified (see “Discussion”).

### Residues involved in oligonucleotide binding and the recognition of the GpsN pattern are highly conserved in prokaryotic variants of SAMHD1

**Fig. 5:**
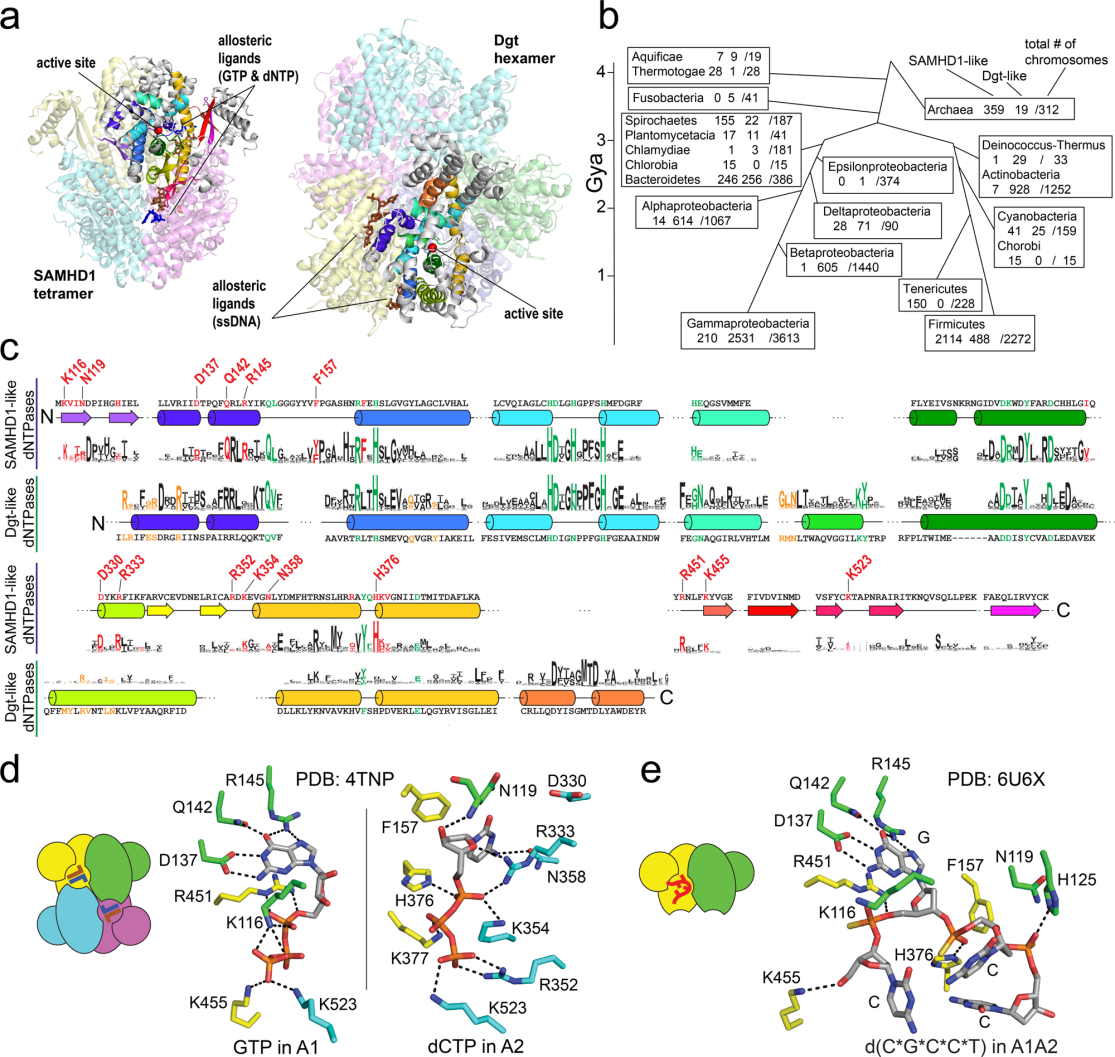
Functional determinants of sequence conservation in prokaryotic SAMHD1-like dNTPases. **a** Cartoon representations of the SAMHD1 tetramer (PDB: 4BZC) and Dgt hexamer (PDB:4X9E). The two structures are shown such that the conserved secondary structure elements in one of the monomers (color-coded) are oriented the same way. **b** Census of HD-domain dNTPases in a database of complete prokaryotic chromosomes. The three numbers shown are the total number of identified SAMHD1-like dNTPases, Dgt-like dNTPases, and the total number of searched chromosomes, respectively. Each taxon is displayed linked to the time of its common ancestor with Gammaproteobacteria or Firmicutes^93^. **c** Sequence logo representations of multiple-sequence alignments for the SAMHD1-like and Dgt-like families of prokaryotic dNTPases. The secondary structure cartoons show the location of conserved secondary structure elements in the primary sequence of SAMHD1 and Dgt positioned above and below the secondary structure cartoons, respectively. Residues located within 5 Å of the active sites are colored green. Residues located within 5 Å of the allosteric ligands are colored red and orange in SAMHD1 and Dgt, respectively. Residues surrounding the SAMHD1 allosteric sites are also labeled. **d** Interactions of GTP and dCTP with the residues of the allosteric binding sites of SAMHD1^21^ (PDB: 4TNP). **e** Interactions of the d(C*G*C*C*T) oligonucleotide with the allosteric sites (PDB:6U6X).

HD-domain dNTP hydrolases can be identified in prokaryotes by reverse PSIBLAST screening with models from the NCBI-conserved domain database COG1078 YdhJ and COG0232 Dgt. The HD domain of SAMHD1 is detected by the YdhJ profile that was composed most heavily from Archaeal sequences with a secondary component from Gram-positive bacteria and a few representatives from other phyla, including one from yeast. The Dgt profile, named after the *E. coli* Dgt dNTPase, is most heavily composed of sequences from Gammaproteobacteria, but with components from other scattered phyla. Known structures of prokaryotic HD-domain hydrolases also fall into two distinct families—SAMHD1-like and Dgt-like—by structural comparison (Fig. 5a). Specifically, dNTPases from *Enterococcus faecalis* (PDB: 3IRH), *Bacteroides thetaiotaomicron* (PDB: 2Q14), and *Aquifex aeolicus* (PDB: 2HEK), as well as the HD domain of SAMHD1 itself (e.g., PDB: 4BZB) are highly similar throughout their entire HD-domain structures, which consist of a large catalytic lobe (N-lobe) containing a characteristic N-terminal beta hairpin and a smaller C-terminal lobe (C-lobe) with a distinctive extended coil element that contributes to multimerization and allosteric activation in SAMHD1 (Supplementary Fig. 4). Structures of the Dgt-like enzymes, which include dNTPases of *E. coli* (PDB: 4X9E), *Thermus thermophilus* (PDB: 2DQB), *Pseudomonas syringae* (PDB: 2PGS), and *Flavobacterium sp. MED217* (PDB: 3BG2), are also very similar to each other but differ from SAMHD1-like structures. They lack the C-lobe, and two beta-hairpin elements conserved in the SAMHD1 family are not present in their catalytic lobes.

The two distinct families of prokaryotic HD-domain dNTPases were explored further through sequence searching. A complete census from completely sequenced bacterial chromosomes was derived and HD-domain hydrolase sequences were assigned to either the SAMHD1-like family or the Dgt-like family as described in “Methods” (Fig. 5b). Both families were found in virtually every phylum in bacteria, and both were also found in Archaea. Both families are auxiliary genes in bacteria, meaning not found in every genome of a given taxon, with individual chromosomes having 0, 1, 2, or 3 representatives. We also found cases of both SAMHD1-like and Dgt-like dNTPases present in the same genome. The multiplicity of paralogs in individual genomes suggests the existence of functionally specialized variants and the inconsistent copy numbers suggest movement within taxons by horizontal transfer. The nature of two functional Dgt-like paralogs has been characterized in *Pseudomonas*^61^. However, there is evidence that some of the instances are pseudogenes (see “Methods”). In the two bacterial phyla most heavily sampled by sequenced genomes, the SAMHD1-like family dominates the Gram-positive Firmicutes phylum, and the Dgt-like family dominates the Gram-negative Proteobacteria phylum. The preference for the Dgt family was stable through the descent of the Proteobacterial classes (alpha, beta, gamma, delta, and epsilon), but preference for family was not stable among phyla generated earlier in the history of the earth. The multiple-sequence alignments for the two families were thinned as described in “Methods” to provide a normalized representation of sequence variation across all prokaryotes, and sequence logos were prepared to visualize residue conservation patterns (Fig. 5c).

Sequence analysis reveals that similar architectures of the active sites explain residue conservation patterns shared by both dNTPase families (Fig. 5a, c). In contrast, oligomerization and allosteric activation mechanisms are different in the SAMHD1-like and Dgt-like enzymes, which explains the conservation patterns that distinguish the two families. For example, the recognition of the guanine base at the A1 site mediated by D137, Q142, R145, and R451 in SAMHD1 is a highly conserved functionality in the SAMHD1-like prokaryotic dNTPases, but not in the Dgt-like enzymes (Fig. 5c–e). The biological significance of SAMHD1 dependence on GTP or dGTP binding at the A1 site remains unknown, and the contribution of these residues to the recognition of the GpsN modification offers one potential explanation. Another striking feature is the remarkably high conservation of the phosphorothioate-interacting residue, H376. Indeed, H376 is one of the most conserved residues in the SAMHD1-like family of prokaryotic dNTPases on par with the histidines coordinating the catalytic metal of the active site. In the GTP-/dNTP-bound structures of SAMHD1, H376 forms a hydrogen bond with the alpha phosphate of the dNTP ligand bound in A2, but its conservation in prokaryotic enzymes is remarkably higher than that of other residues making similar hydrogen bonds with the triphosphate portion of the dNTP in A2 site of SAMHD1 (e.g., K354, K377, R352, or K523) (Fig. 5d, e). Once again, the role of H376 in GpsN recognition could explain its exceptional conservation.

### Mutations that impair formation of the oligonucleotide-bound tetramer abolish the antiretroviral activity of SAMHD1, but not its ability to deplete dNTPs

**Fig. 6:**
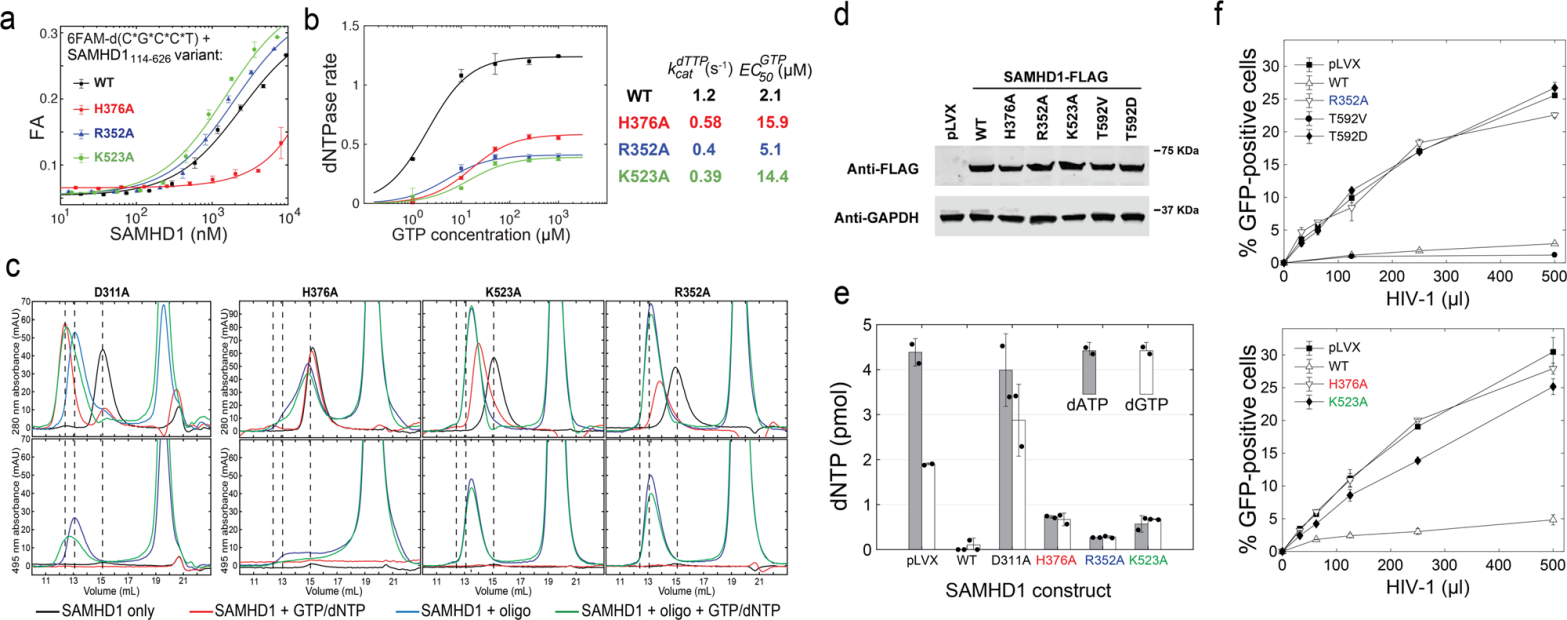
Functional effects of H376A, R352A, and K523A mutations. **a** Oligonucleotide-binding affinities of allosteric site mutants measured using the fluorescence anisotropy-binding assay (*n* = 2 independent experiments). **b** In vitro dNTPase activities of allosteric site mutants (*n* = 2 independent experiments). **c** Oligonucleotide binding and oligomerization properties of allosteric site mutants as monitored by SEC. **d** Expression of SAMHD1 variants in U937 cells. **e** dNTP depletion in U937 cells by WT and mutant SAMHD1 variants (*n* = 2 independent experiments). **f** HIV-1 restriction activity of WT and mutant SAMHD1 variants (*n* = 2 independent experiments). Error bars represent s.d. of two replicate measurements performed on distinct samples. Source data are provided as a Source Data File.

Finally, we used site-directed mutagenesis to evaluate the contribution of oligonucleotide binding to the antiretroviral function of SAMHD1. Oligonucleotides and nucleotide triphosphates bind at overlapping sites and the protein can form mixed-occupancy tetramers, making it difficult to explore the function of these distinct ligands independently using mutagenesis. We generated point mutants—H376A, R352A, and K523A—of three residues making similar hydrogen bonds with the triphosphate segment of dNTP bound in A2. The key difference between the three residues is that H376 mediates a critical interaction in oligonucleotide binding and is highly conserved in prokaryotic SAMHD1-like enzymes, whereas R352 and K523 do not interact with oligonucleotides and their conservation in prokaryotes is less pronounced (Fig. 5c–e). In agreement with structural data, H376A diminished oligonucleotide binding by SAMHD1 as evaluated by fluorescence anisotropy (Fig. 6a) and SEC (Fig. 6c), whereas R352A and K523A displayed similar oligonucleotide-binding affinities to WT and could both form dimers upon oligonucleotide binding. As expected, all three mutants diminished the ability of the protein to tetramerize in the presence of GTP/dNTP and displayed comparable reductions in the dNTPase activity in vitro (Fig. 6b, c). Notably, this partial in vitro dNTPase defect did not impair the ability of the enzyme to deplete dNTP levels in U937 cells as monitored by the primer extension assay following MTA-induced differentiation (Fig. 6d, e). All three mutations abolished the antiretroviral activity of SAMHD1, despite the ability of the mutant enzymes to deplete cellular dNTPs (Fig. 6d, f). We also evaluated whether the protein expression levels had an effect on the ability of the protein to deplete dNTPs and to restrict HIV replication (Supplementary Fig. 5). To this end, we investigated distinct stably transduced cell lines whose expression levels of WT and H376A variants of SAMHD1 differed up to fourfold. We observed that differences in SAMHD1 abundance had no significant effect on the efficiency of dNTP depletion or HIV restriction. Intriguingly, we observed small, but reproducible, differences in the dNTP depletion efficiency between WT and H376 variants in this series of experiments, which indicated that some important features of dNTP depletion may not be fully captured in the measurements of the total cellular dNTP content (see “Discussion”). Overall, the retroviral restriction defect correlated with the inability of all three mutations to form mixed-occupancy tetramers in the presence of oligonucleotides and nucleotide triphosphates (Fig. 6c, f). This was further supported by oligonucleotide titration experiments analyzed by SEC (Supplementary Fig. 5), which revealed a twofold increase in the oligonucleotide-binding stoichiometry of the R352 and K523 mutants compared to D311A variant. These observations suggest that oligonucleotide-dependent SAMHD1 dimerization is not sufficient for restriction, whereas formation of the oligonucleotide-bound, mixed-occupancy SAMHD1 tetramer that is lost in all three mutants H376A, R352, and K523, is required for the antiretroviral activity of the protein.

## Discussion

Studies by the Stivers group revealed that the interaction of SAMHD1 with single-stranded RNA and DNA oligonucleotides traps the enzyme in the inactive dimeric state and mapped an extensive nucleic acid footprint on the SAMHD1 surface using chemical cross-linking^47,48^. Here we offer atomic resolution insight into the interaction of SAMHD1 with nucleic acids. We show that the allosteric sites of SAMHD1 contribute to nucleic binding owing to their ability to accommodate oligonucleotides in place of the allosteric activators, GTP and dNTP. In agreement with findings by Stivers et al., we observe that oligonucleotide binding to the allosteric sites promotes SAMHD1 dimerization. At high concentrations, oligonucleotides can completely displace nucleotide triphosphates from the allosteric sites, which explains the inhibition of the dNTPase activity of SAMHD1 by oligonucleotide binding observed in earlier in vitro work^44–48^. Furthermore, we show that oligonucleotide binding to the allosteric sites can be sequence- and structure-specific because the presence of the GpsN modification enhances oligonucleotide- binding affinity and makes the interaction much less sensitive to the ionic strength. The findings are reminiscent of transcription factors, whose binding to nucleic acids is often a combination of nonspecific electrostatic interactions that are sensitive to ionic strength and interactions that are sequence-dependent and salt-tolerant^62^. The balance between these two distinct types of interactions may enhance target search efficiency by transcription factors^63^. Similarly, nucleic acid binding by SAMHD1 appears to combine nonspecific, salt-sensitive interactions mapped by Seamon et al.^48^ and the salt-tolerant, structure-, and sequence-specific interactions with the allosteric sites described here. The study offers a glimpse into how specific interactions with nucleic acids can alter allosteric properties of SAMHD1 and suggests that the immune function of the protein may be modulated by specific nucleic acid ligands that bind to SAMHD1 with relatively high affinity. The exact functional impact of these putative ligands is difficult to predict from our data with short GpsN-containing oligos, because the physiological nucleic acid-binding partners of SAMHD1 may have multiple binding sites and may engage additional interaction surfaces as suggested by the earlier cross-linking studies. Identification of specific nucleic acid modulators of SAMHD1 is therefore of great interest and will also reveal whether the GpsN modification is the physiologically relevant determinant of SAMHD1 specificity^53,54^ (see below) or whether it simply mimics some other structural feature present in these ligands.

**Fig. 7:**
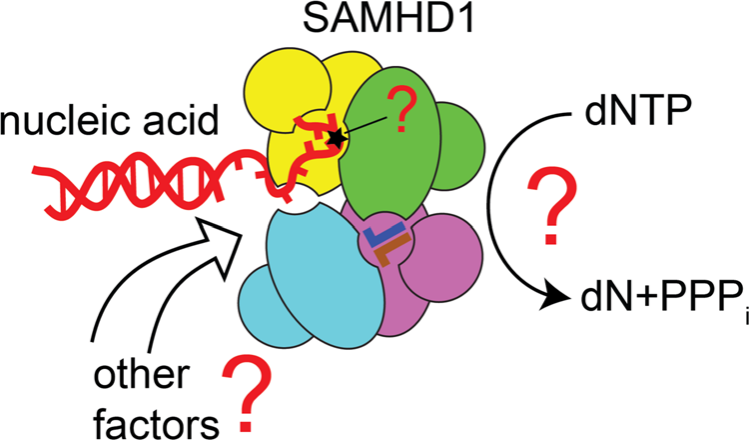
Contribution of nucleic acid binding to the antiviral activity of SAMHD1. Oligonucleotide-dependent oligomerization of SAMHD1 may function as a distinct dNTPase activation mechanism that is critical for restriction of retroviral replication but not for the overall depletion of cellular dNTP content. Alternatively, it may contribute to retroviral restriction in a dNTPase-independent fashion by stalling reverse transcription, recruiting other factors, or promoting degradation of viral nucleic acids. The GpsN modification (black star) may be involved in human innate immunity or may simply mimic some other structural feature present in the physiological nucleic acid ligands of SAMHD1.

We show that short GpsN-containing oligonucleotides, when added at substoichiometric amounts in the presence of GTP and dNTPs, promote formation of SAMHD1 tetramers with mixed occupancy of the allosteric sites and altered functionality. Mutagenesis experiments reveal a correlation between the formation of the oligonucleotide-bound, mixed-occupancy SAMHD1 tetramer and the restriction of retroviral replication. Remarkably, the three mutations—H376A, R352A, and K523A—recapitulate the puzzling phenotype previously described for the T592 phosphomimetics and several other SAMHD1 mutants: they abolish the antiretroviral activity, but not the dNTP depletion^23,25,27^. These observations suggest that interactions with nucleic acids may help explain the apparent contradiction between the presumed dNTPase-dependent mechanism of retroviral restriction and the decoupling between dNTP depletion and antiviral activity displayed by several SAMHD1 mutants. One possibility is that the mixed-occupancy tetramer represents an alternative dNTPase activation mechanism. The oligonucleotide-dependent dNTPase activity may be responsible for some spatiotemporal aspect of dNTP depletion that is critical for restriction, but not apparent in the measurements of the total cellular dNTP content. For example, nucleic acid binding may be required for robust dNTP depletion in the cytosol, whereas measurements of the total cellular dNTPs may be dominated by an oligonucleotide-independent mechanism and the residual dNTP content of the nucleus and other compartments. An alternative possibility is that a dNTPase-independent functionality of the oligonucleotide-bound SAMHD1 tetramer is required for restriction in addition to dNTP depletion. For example, SAMHD1 binding to nucleic acid intermediates of reverse transcription may create a steric block that exacerbates dNTP shortage and stalls reverse transcription. SAMHD1 may also recruit other factors that target paused RT or viral nucleic acid intermediates made vulnerable to degradation by the slow rate of reverse transcription at low dNTP levels (Fig. 7).

Recognition by SAMHD1 of the GpsN pattern, the modification known to occur naturally in the poorly understood restriction-modification mechanism of exogenous DNA suppression in prokaryotes^50–52^, hints at a functional link between nucleic acid phosphorothioation, innate immunity, and dNTP depletion. Although the effect of phosphorothioate modification on specific protein binding has been known for decades^64,65^, more recent work on the development of oligonucleotide therapies uncovered distinctive immunomodulatory properties of some phosphorothioated oligonucleotides^66,67^. Phosphorothioation promotes oligonucleotide binding to a subset of cellular proteins^64,68,69^, and the stereochemistry and location of phosphorothioate bonds substantially affects oligonucleotide bioactivity^70,71^. These observations prompted development of new methods for stereocontrolled installation of this chiral motif^72^. Our study reveals how chirality and positioning of phosphorothioate linkages can impact interaction of oligonucleotides with cellular proteins and opens a path for structure-guided optimization of these promising new therapeutics. SAMHD1 is probably not the only immune factor, whose activity is modulated by phosphorothioation in a sequence- and stereochemistry-dependent fashion. For example, phosphorothioation potentiates TLR9 activation by the CpG agonists, which are being explored as vaccine adjuvants and in cancer therapy^73^. In a striking parallel with the role of H376 in SAMHD1, TLR9 contains a conserved histidine (H641 in the human variant) that interacts with the phosphodiester linkage 3′ to the G of the CpG motif in the crystal structure of CpG-bound TLR9^74^. H641D mutation reduces or abolishes TLR9 activation in response to phosphorothioated CpG agonists^71^. Finally, two recent publications raise an intriguing possibility that GpsN recognition by SAMHD1 described here contributes to its immune function in humans. First, the GpsG modification has been detected in human RNA^54^ and, second, DNA phosphorothioation has been found widespread in the human microbiome^53^.

In summary, our results implicate nucleic acid binding in the antiretroviral activity of SAMHD1, shed new light on the immunomodulatory effects of synthetic phosphorothioated oligonucleotides, and raise questions about the role of nucleic acid phosphorothioation in human innate immunity. The structural and mechanistic insight offered by our work will help explore whether and how GpsN modifications in nucleic acids modulate dNTP metabolism and immune responses.

## Methods

### Preparation of proteins and oligonucleotides

The WT and mutant variants (Supplementary Table 1) of the full-length SAMHD1_1–626_ and HD-domain SAMHD1_114–626_ constructs were cloned into pET30 vectors (Novagen) and transformed into *E. coli* BL21(DE3) cells. Bacterial cells were grown in LB media at 37 °C to an OD_600_ ~0.6. Protein expression was induced by adding 1 mM isopropyl-β-d-thiogalactopyranoside followed by overnight incubation at 20 °C. Cells were harvested by centrifugation at 6000 × *g* for 15 min. To purify the protein, cells were disrupted by sonication and the cell lysate was centrifuged at 39,191 × *g* for 60 min. The supernatant was loaded onto a Strep-Tactin Sepharose column (IBA) equilibrated with 50 mM TRIS, pH 8, 1 M NaCl, and 20 mM β-mercaptoethanol. The protein was eluted with 2.5 mM desthiobiotin (IBA). The eluted proteins were further purified on a Superdex 200 column (GE Healthcare) containing 50 mM TRIS, pH 8, 100 mM NaCl, and 1 mM TCEP.

DNA and RNA oligonucleotides were ordered from either IDT or GE Dharmacon. ssDNA^57^ and ssRNA^40^ had the same sequences as in the original study^47^. Commercial phosphorothioated oligonucleotides contain roughly equimolar mixtures of the *Rp* and *Sp* stereoisomers for each phosphorothioate group. Pure *Rp* and *Sp* stereoisomers of d(TG*TTCA) and 6FAM-d(TG*TTCA) oligonucleotides were isolated by reverse-phase chromatography (Fig. 4b, f) on the Vydac C-18 column 218TP1010 (preparative 10 × 250 mm, 10-micron particle size, ~19-mL bed volume). Separation was achieved by applying a 90-mL 5.75–19.25% acetonitrile gradient in 50 mM TEAA, pH 7.0, at 3 mL/min flow rate.

### Crystallization, structure determination, and refinement

Automated screening for crystallization was carried out using the sitting-drop vapor-diffusion method with an Art Robbins Instruments Phoenix system in the X-ray Crystallography Core Laboratory at the University of Texas Health Science Center at San Antonio. SAMHD1:d(TG*TTCA) crystals were obtained at 22 °C from the Qiagen PACT screen condition #96 and optimized to 0.26 M sodium malonate, 20% w/v polyethylene glycol 3350, and 0.1 M bis Tris propane, pH 8.5, using an Art Robbins Instruments Scorpion system. A solution containing 4.5 mg/mL WT SAMHD1_114–626_, 50 mM Tris, pH 8.0, 100 mM NaCl, 1 mM TCEP, and 300 μM d(TG*TTCA) DNA was mixed 1:1 with well solution to a total volume of 1 μL and suitable crystals were produced by streak-seeding the crystal drop with a solution of crushed crystals. D311A SAMHD1:d(C*G*C*C*T) crystals were obtained at 22 °C from Microlytic MCSG-II condition #71 (0.2 M sodium tartrate dibasic, 20% w/v polyethylene glycol 3350) mixed 1:1 with 140 μM D311A SAMHD1_114–626_, 50 mM Tris, pH 8.0, 50 mM NaCl, 1 mM TCEP, and 250 μM d(C*G*C*C*T) DNA to a total volume of 0.4 μL. D311A SAMHD1:r(CGCCU) crystals were obtained at 22 °C from Microlytic MCSG-I condition #13 (0.6 M sodium chloride, 20% w/v polyethylene glycol 4000, and 0.1 M 2-[*N*-morpholino]ethanesulfonic acid:sodium hydroxide, pH 6.5) mixed 1:1 with 100 μM SAMHD1 D311A, 50 mM Tris, pH 8.0, 50 mM NaCl, 1 mM TCEP, and 500 μM CGCCU RNA to a total volume of 0.4 μL. The crystals were flash-cooled in liquid nitrogen prior to data collection at the Advanced Photon Source, Argonne, IL, beamlines 24-ID-C and 24-ID-E. Diffraction data were processed using *xia2*^75–78^ to resolution limits with CC_1/2_ ≥ 0.5^79^ and mean *Ι* / *σΙ* ≥ 1.0.

The structures of the SAMHD1 complexes were determined by the molecular replacement method implemented in PHASER^80^ using coordinates of SAMHD1 monomer from PDB entry 3U1N3 as the search model. Coordinates for each complex were refined using PHENIX^81^, including simulated annealing and TLS refinement, alternated with manual rebuilding using COOT^82^. Noncrystallographic symmetry positional and B-factor restraints were applied to the 2.6-Å D311A SAMHD1:CpsGpsCpsCpsT structure. An additional 720° of long-wavelength data at 1.7712 Å (7000 eV) were collected for the SAMHD1:TGpsTTCA crystal to boost the anomalous signal of the sulfur atoms in the guanosine phosphorothioate for anomalous difference Fourier analysis. The 720° sweep was experimentally determined to be the optimum strategy with 0.2-s exposures per 0.2° oscillation as collecting below this amount was insufficient to identify significant difference anomalous Fourier peaks and collecting beyond it generated substantial radiation damage to the crystal. All models were verified using composite omit map analysis^83^. Data collection and refinement statistics are shown in Table 1.

### Fluorescence-polarization assays of oligonucleotide binding

Oligonucleotide-binding assays were performed using oligonucleotides labeled at the 5′ end with 6FAM. Increasing concentrations of SAMHD1 variants were titrated into samples containing 50 nM of fluorescent oligonucleotide in 50 mM TRIS, pH 8, 100 mM NaCl, 5 mM MgCl_2_, and 1 mM TCEP. Fluorescence-polarization measurements were performed in 384-well plates (Corning 3575) on a Synergy 2 microplate reader (Biotek) using 485-/20-nm excitation and 530-/20-nm emission band-pass filters. All experiments were performed in duplicate with 20-μL solution volume in each well.

### Analytical ultracentrifugation and size-exclusion chromatography

SAMHD1 constructs were incubated with oligonucleotides and/or GTP/dNTP mixtures in 50 mM TRIS, pH 8, 100 mM NaCl, 5 mM MgCl_2_, and 0.5 mM TCEP. In all, 450-μL samples of A_280_~1.0 were loaded into titanium cells with quartz windows and sedimented at 141,995 × *g* at 20 °C in an Optima AUC (Beckman-Coulter) ultracentrifuge equipped with a 8-hole An50-Ti rotor. Absorbance readings were taken at 280 or 280/495 nm every 5 min. Fluorescence readout experiments were performed using an AUC detector from Aviv Biomedical. All data were analyzed with UltraScan-III by two-dimensional spectrum analysis^84–86^.

Stoichiometry in SAMHD1_114–626_:6FAM-d(C*G*C*C*T) complexes was investigated by SEC using a Superdex 200 10/300 GL column (GE Healthcare) and a BIO-RAD NGC chromatography system with multiwavelength UV-VIS absorbance detection capability (Biorad ChromLab) (Fig. 4a–c and Supplementary Figs. 3 and 5D, E). A fixed amount of purified SAMHD1 (~5 μM in Fig. 4a–c and ~2.5–3 μM in Supplementary Figs. 3 and 5D, E) was mixed with increasing amounts of 6FAM-d(C*G*C*C*T) with or without nucleotide triphosphates present (50 μM GTP and 50 μM of different dNTPs as specified). In the experiments with nucleotide triphosphates, 50 μM GTP and 50 μM of dNTP were also included in the running buffer. The running buffer contained 50 mM TRIS, pH 8, 100 mM NaCl, 5 mM MgCl_2_, and 1 mM TCEP. Total SAMHD1 concentration [SAMHD1]_*t*_ was determined by integrating the 280-nm absorbance peak of monomeric SAMHD1 with no oligonucleotide and no GTP/dNTP present. SAMHD1-bound 6FAM-d(C*G*C*C*T) was quantified by integrating 495-nm absorbance peaks of the chromatogram.

### NMR spectroscopy and dNTPase assays

NMR samples of oligonucleotides with or without SAMHD1 present were prepared in the buffer containing 50 mM Tris, pH 7.5, 150 mM NaCl, 5 mM MgCl_2_, and 10% D_2_O. ^31^P NMR spectra were acquired on a Bruker 700-MHz spectrometer equipped with a 5-mm room-temperature broadband RF probe. ^1^H NMR spectra were acquired on a 500-MHz spectrometer equipped with a 1.7-mm cryoprobe.

Kinetics of dNTP hydrolysis catalyzed by SAMHD1 was investigated using an NMR-based assay. Proton NMR spectra were recorded at regular time intervals and the relative peak intensity of the H6 proton signal of deoxythymidine triphosphate (substrate) versus deoxythymidine nucleoside (product) was measured as a function of time. The rate of dNTP hydrolysis was determined by linear fitting of the hydrolysis reaction curves using MATLAB software (Mathworks).

### Sequence analysis of prokaryotic HD-domain dNTPases

In order to explore the distribution of HD-domain dNTPases in prokaryotes, we made use of the library of proteins from completely sequenced bacterial genomes (February 2018 version) described in^87^, and local implementations of the BLAST toolkit^88^, the HHpred system^89^, the UCSC Sequence Analysis and Alignment system^90^, PAUP (https://paup.phylosolutions.com), and MrBayes 3.2.7^91^. The library is formatted to contain protein accession, nucleotide accession, and full taxonomic distribution on each protein sequence definition line, making it possible to directly examine the distribution within genomes and taxons of any clade found within the library.

Sequences of two prokaryotic HD-domain dNTPases of known structure, *E. coli* Dgt (PDB:4X9E) and *Enterococcus Faecalis* (PDB:3IRH), representing the Dgt-like and SAMHD1-like dNTPase families, respectively, were used as starting sequences in PSIBLAST homology searches. Lists of round 5 PSIBLAST matches for each search included members of the other family in some semblance of a fully aligned form and were truncated at the point where the first major bolus of sequences of the other family appeared. Sequences in the lists were scored using HHpred against 4x9e_A and 4lrl_A models retrieved from the HHpred website. All sequences preferred one or the other model by a factor of at least 10^18^ in *E* value and were thus assigned to one or the other of the two families. UCSC sequence alignment and modeling system was used to analyze the Dgt-like and SAMHD1-like sequence sets and also of the joint set. The analysis confirmed that the search and assignment procedure resulted in sequence sets that were near-complete and not contaminated with distantly related HD-domain sequences of non-dNTPase function. Numerous members of both sets appeared truncated at either the N or the C terminus. Spot-checking revealed examples of larger genes disrupted by frameshifts or premature terminators, and genes with the start codon misannotated at an internal position. The distribution of the truncated genes was mapped on the tree and they were not clustered. Hence, these members are believed to be legitimate dNTPase genes obscured by a consequence of gene inactivation, sequencing error, or annotation errors, and not to be a clade with shorter functional protein length. These sequences were excluded from the lists used for the final sequence alignment.

The taxonomic descriptions from the GenBank files were used to count the number of dNTPases of each family within taxons and the total numbers of sequenced chromosomes belonging to each taxon (Supplementary Fig. 4). There was no correction applied for the fact that some bacteria have two chromosomes. Only circular chromosomes of >200,000 bp were included in the analysis.

The Dgt-like and SAMHD1-like sequence sets were aligned separately to generate Logo representations of the two families (Supplementary Fig. 5). In order to normalize against heavy overrepresentation in some clades, the alignments were thinned about tenfold to contain only one sequence of each group within 70% identity. Because of low sequence homology within the C-lobe of the SAMHD1-like family, the multiple-sequence aligner failed to recapitulate the structural alignment of elements in the C-lobes of the four SAMHD1-like dNTPases of known structure. In order to overcome this, an iterative, structure-aided alignment of the C-lobe was performed using the HMM building tool of the UCSC sequence alignment and modeling system. Logos were computed at http://weblogo.threeplusone.com.

### Generation of U937 cells stably expressing SAMHD1 variants

Retroviral vectors encoding wild-type or mutant SAMHD1 proteins fused to the N-terminal FLAG peptide tag were created using the LPCX vector (Clontech). Recombinant viruses were produced in 293FT cells by cotransfecting the LPCX plasmids with the pVPack-GP and pVPack-VSV-G packaging plasmids (Stratagene). The pVPack-VSV-G plasmid encodes the vesicular stomatitis virus G envelope glycoprotein, which allows efficient entry into a wide range of vertebrate cells. Transduced human monocytic U937 cells were selected in 0.4 μg/ml puromycin (Sigma). Distinct selected cell lines differ in their levels of protein expression. These differences were used to evaluate the effect of protein expression levels on dNTP concentrations and HIV restriction (Supplementary Fig. 5A–C).

### Restriction assays

Recombinant retroviruses expressing GFP, pseudotyped with the VSV-G glycoprotein, were prepared as described^92^. For infections, 6 × 10^4^ cells seeded in 24-well plates were either treated with either concentration 10 ng/mL phorbol-12-myristate-3-acetate (PMA) or DMSO for 16 h. PMA stock solution was prepared in DMSO at 250 μg/mL. Subsequently, cells were incubated with the indicated retrovirus for 48 h at 37 °C. The percentage of GFP-positive cells was determined by flow cytometry (Becton Dickinson). Viral stocks were titrated by serial dilution on dog Cf2Th (ATCC# CRL-1430) cells.

### Cellular dNTP quantification

In total, 2 × 10^6^ to 3 × 10^6^ cells were collected for each cell type. Cells were washed twice with 1× PBS, pelleted, and resuspended in ice-cold 65% methanol. Cells were vortexed for 2 min and incubated at 95 °C for 3 min. Cells were centrifuged at 16,000 × *g* for 3 min and the supernatant was transferred to a new tube for the complete drying of methanol in a speed vac. The dried samples were resuspended in molecular-grade dH_2_O. An 18-nucleotide primer labeled at the 5′ end with 32 P (5′-GTCCCTGTTCGGGCGCCA-3′) was annealed at a 1:2 ratio to four different 19-nucleotide templates (5′-NTGGCGCCCGAACAGGGAC-3′), where “N” represents the nucleotide variation at the 5′ end. The reaction condition contains 200 fmoles of template primer, 2 μL of 0.5 mM dNTP mix for positive control or dNTP cell extract, 4 μL of excess HIV-1 RT, 25 mM Tris-HCl, pH 8.0, 2 mM dithiothreitol, 100 mM KCl, 5 mM MgCl_2_, and 10 μM oligo(dT) to a final volume of 20 μL. The reaction was incubated at 37 °C for 5 min before being quenched with 10 μL of 40 mM EDTA and 99% (vol/vol) formamide at 95 °C for 5 min. The extended primer products were resolved on a 14% urea–PAGE gel and analyzed using a phosphoimager. The extended products were quantified using QuantityOne 4.6.8 software to quantify percent volume of saturation. The quantified dNTP content of each sample was accounted for based on its dilution factor, so that each sample volume was adjusted to obtain a signal within the linear range of the assay.

### Antibodies

The following antibodies used were monoclonal ANTI-FLAG M2 antibody produced in mouse (F1804-5MG, Sigma-Aldrich) at a dilution of 1:1000 and anti-glyceraldehyde-3-phosphate dehydrogenase (AM4300, Invitrogen) at a dilution of 1:5000.

### Reporting summary

Further information on research design is available in the Nature Research Reporting Summary linked to this article.

## Supplementary information

Supplementary Information

Reporting Summary

## Data Availability

Data supporting the findings of this paper are available from the corresponding authors upon reasonable request. Atomic coordinates and structure factors have been deposited in the Protein Data Bank under accession codes PDB 6U6Y [ribo(CGCCU)], PDB 6U6X [deoxy(C*G*C*C*T)], and PDB 6U6Z [deoxy(TG*TTCA)]. Source data are provided with this paper.

## Source data

Source Data

## References

[1] Laguette N (2011). SAMHD1 is the dendritic- and myeloid-cell-specific HIV-1 restriction factor counteracted by Vpx. Nature.

[2] Hrecka K (2011). Vpx relieves inhibition of HIV-1 infection of macrophages mediated by the SAMHD1 protein. Nature.

[3] Goldstone DC (2011). HIV-1 restriction factor SAMHD1 is a deoxynucleoside triphosphate triphosphohydrolase. Nature.

[4] Lahouassa H (2012). SAMHD1 restricts the replication of human immunodeficiency virus type 1 by depleting the intracellular pool of deoxynucleoside triphosphates. Nat. Immunol..

[5] Fujita M (2008). Vpx is critical for reverse transcription of the human immunodeficiency virus type 2 genome in macrophages. J. Virol..

[6] Goujon C (2007). SIVSM/HIV-2 Vpx proteins promote retroviral escape from a proteasome-dependent restriction pathway present in human dendritic cells. Retrovirology.

[7] Kim B, Nguyen LA, Daddacha W, Hollenbaugh JA (2012). Tight interplay among SAMHD1 protein level, cellular dNTP levels, and HIV-1 proviral DNA synthesis kinetics in human primary monocyte-derived macrophages. J. Biol. Chem..

[8] Franzolin E (2013). The deoxynucleotide triphosphohydrolase SAMHD1 is a major regulator of DNA precursor pools in mammalian cells. Proc. Natl Acad. Sci. USA.

[9] Baldauf HM (2012). SAMHD1 restricts HIV-1 infection in resting CD4(+) T cells. Nat. Med..

[10] Diamond TL (2004). Macrophage tropism of HIV-1 depends on efficient cellular dNTP utilization by reverse transcriptase. J. Biol. Chem..

[11] Guyader M, Emerman M, Montagnier L, Peden K (1989). VPX mutants of HIV-2 are infectious in established cell lines but display a severe defect in peripheral blood lymphocytes. EMBO J..

[12] Schwefel D (2014). Structural basis of lentiviral subversion of a cellular protein degradation pathway. Nature.

[13] Berglund O, Karlstrom O, Reichard P (1969). A new ribonucleotide reductase system after infection with phage T4. Proc. Natl Acad. Sci. USA.

[14] Dwivedi B, Xue B, Lundin D, Edwards RA, Breitbart M (2013). A bioinformatic analysis of ribonucleotide reductase genes in phage genomes and metagenomes. BMC Evol. Biol..

[15] Beauchamp BB, Richardson CC (1988). A unique deoxyguanosine triphosphatase is responsible for the optA1 phenotype of *Escherichia coli*. Proc. Natl Acad. Sci. USA.

[16] Kornberg SR, Lehman IR, Bessman MJ, Simms ES, Kornberg A (1958). Enzymatic cleavage of deoxyguanosine triphosphate to deoxyguanosine and tripolyphosphate. J. Biol. Chem..

[17] Huber HE, Beauchamp BB, Richardson CC (1988). *Escherichia coli* dGTP triphosphohydrolase is inhibited by gene 1.2 protein of bacteriophage T7. J. Biol. Chem..

[18] Ji X (2013). Mechanism of allosteric activation of SAMHD1 by dGTP. Nat. Struct. Mol. Biol..

[19] Yan J (2013). Tetramerization of SAMHD1 is required for biological activity and inhibition of HIV infection. J. Biol. Chem..

[20] Zhu C (2013). Structural insight into dGTP-dependent activation of tetrameric SAMHD1 deoxynucleoside triphosphate triphosphohydrolase. Nat. Commun..

[21] Ji X, Tang C, Zhao Q, Wang W, Xiong Y (2014). Structural basis of cellular dNTP regulation by SAMHD1. Proc. Natl Acad. Sci. USA.

[22] Koharudin LM (2014). Structural basis of allosteric activation of sterile alpha motif and histidine-aspartate domain-containing protein 1 (SAMHD1) by nucleoside triphosphates. J. Biol. Chem..

[23] Bhattacharya A (2016). Effects of T592 phosphomimetic mutations on tetramer stability and dNTPase activity of SAMHD1 can not explain the retroviral restriction defect. Sci. Rep..

[24] Brandariz-Nunez A (2013). Contribution of oligomerization to the anti-HIV-1 properties of SAMHD1. Retrovirology.

[25] White TE (2013). The retroviral restriction ability of SAMHD1, but not its deoxynucleotide triphosphohydrolase activity, is regulated by phosphorylation. Cell Host Microbe.

[26] Cribier A, Descours B, Valadao AL, Laguette N, Benkirane M (2013). Phosphorylation of SAMHD1 by cyclin A2/CDK1 regulates its restriction activity toward HIV-1. Cell Rep..

[27] Welbourn S, Dutta SM, Semmes OJ, Strebel K (2013). Restriction of virus infection but not catalytic dNTPase activity is regulated by phosphorylation of SAMHD1. J. Virol..

[28] Antonucci JM (2018). SAMHD1 impairs HIV-1 gene expression and negatively modulates reactivation of viral latency in CD4(+) T cells. J. Virol.

[29] Kueck T, Cassella E, Holler J, Kim B, Bieniasz PD (2018). The aryl hydrocarbon receptor and interferon gamma generate antiviral states via transcriptional repression. Elife.

[30] Mlcochova P, Caswell SJ, Taylor IA, Towers GJ, Gupta RK (2018). DNA damage induced by topoisomerase inhibitors activates SAMHD1 and blocks HIV-1 infection of macrophages. EMBO J..

[31] Mlcochova P (2017). A G1-like state allows HIV-1 to bypass SAMHD1 restriction in macrophages. EMBO J..

[32] Osei Kuffour E (2018). USP18 (UBP43) abrogates p21-mediated inhibition of HIV-1. J. Virol..

[33] Schott K (2018). Dephosphorylation of the HIV-1 restriction factor SAMHD1 is mediated by PP2A-B55alpha holoenzymes during mitotic exit. Nat. Commun..

[34] Szaniawski MA (2018). SAMHD1 phosphorylation coordinates the anti-HIV-1 response by diverse interferons and tyrosine kinase inhibition. mBio.

[35] Daddacha W (2017). SAMHD1 promotes DNA end resection to facilitate DNA repair by homologous recombination. Cell Rep..

[36] Rice GI (2009). Mutations involved in Aicardi-Goutieres syndrome implicate SAMHD1 as regulator of the innate immune response. Nat. Genet..

[37] Martinez-Lopez A (2018). SAMHD1 deficient human monocytes autonomously trigger type I interferon. Mol. Immunol..

[38] White TE (2017). A SAMHD1 mutation associated with Aicardi-Goutieres syndrome uncouples the ability of SAMHD1 to restrict HIV-1 from its ability to downmodulate type I interferon in humans. Hum. Mutat..

[39] Herrmann A (2018). The SAMHD1-mediated block of LINE-1 retroelements is regulated by phosphorylation. Mob. DNA.

[40] Zhao K (2013). Modulation of LINE-1 and Alu/SVA retrotransposition by Aicardi-Goutieres syndrome-related SAMHD1. Cell Rep..

[41] White TE (2016). Modulation of LINE-1 Retrotransposition by a Human SAMHD1 Polymorphism. Virol. Rep..

[42] Coquel F (2018). SAMHD1 acts at stalled replication forks to prevent interferon induction. Nature.

[43] Kim YC, Kim KK, Yoon J, Scott DW, Shevach EM (2018). SAMHD1 posttranscriptionally controls the expression of Foxp3 and Helios in human T regulatory cells. J. Immunol..

[44] White TE (2013). Contribution of SAM and HD domains to retroviral restriction mediated by human SAMHD1. Virology.

[45] Beloglazova N (2013). Nuclease activity of the human SAMHD1 protein implicated in the Aicardi-Goutieres syndrome and HIV-1 restriction. J. Biol. Chem..

[46] Ryoo J (2014). The ribonuclease activity of SAMHD1 is required for HIV-1 restriction. Nat. Med..

[47] Seamon KJ, Sun Z, Shlyakhtenko LS, Lyubchenko YL, Stivers JT (2015). SAMHD1 is a single-stranded nucleic acid binding protein with no active site-associated nuclease activity. Nucleic Acids Res..

[48] Seamon KJ, Bumpus NN, Stivers JT (2016). Single-stranded nucleic acids bind to the tetramer interface of SAMHD1 and prevent formation of the catalytic homotetramer. Biochemistry.

[49] Goncalves A (2012). SAMHD1 is a nucleic-acid binding protein that is mislocalized due to aicardi-goutieres syndrome-associated mutations. Hum. Mutat..

[50] Wang L (2007). Phosphorothioation of DNA in bacteria by DND genes. Nat. Chem. Biol..

[51] Zhou X (2005). A novel DNA modification by sulphur. Mol. Microbiol.

[52] Wang L (2011). DNA phosphorothioation is widespread and quantized in bacterial genomes. Proc. Natl Acad. Sci. USA.

[53] Sun Y (2020). DNA phosphorothioate modifications are widely distributed in the human microbiome. Biomolecules.

[54] Wu Y (2020). RNA phosphorothioate modification in prokaryotes and eukaryotes. ACS Chem. Biol..

[55] Bjersing JL, Eriksson K, Tarkowski A, Collins LV (2004). The arthritogenic and immunostimulatory properties of phosphorothioate oligodeoxynucleotides rely on synergy between the activities of the nuclease-resistant backbone and CpG motifs. Inflammation.

[56] Stein CA, Castanotto D (2017). FDA-approved oligonucleotide therapies in 2017. Mol. Ther..

[57] Kim YC (2012). Oligodeoxynucleotides stabilize Helios-expressing Foxp3+ human T regulatory cells during in vitro expansion. Blood.

[58] Wang Z, Bhattacharya A, Villacorta J, Diaz-Griffero F, Ivanov DN (2016). Allosteric activation of SAMHD1 protein by deoxynucleotide triphosphate (dNTP)-dependent tetramerization requires dNTP concentrations that are similar to dNTP concentrations observed in cycling T cells. J. Biol. Chem..

[59] Wang Z (2018). Functionality of redox-active cysteines is required for restriction of retroviral replication by SAMHD1. Cell Rep..

[60] Bartlett PA, Eckstein F (1982). Stereochemical course of polymerization catalyzed by avian myeloblastosis virus reverse transcriptase. J. Biol. Chem..

[61] Mega R, Kondo N, Nakagawa N, Kuramitsu S, Masui R (2009). Two dNTP triphosphohydrolases from *Pseudomonas aeruginosa* possess diverse substrate specificities. FEBS J..

[62] Privalov PL, Dragan AI, Crane-Robinson C (2011). Interpreting protein/DNA interactions: distinguishing specific from non-specific and electrostatic from non-electrostatic components. Nucleic Acids Res..

[63] Esadze A, Kemme CA, Kolomeisky AB, Iwahara J (2014). Positive and negative impacts of nonspecific sites during target location by a sequence-specific DNA-binding protein: origin of the optimal search at physiological ionic strength. Nucleic Acids Res..

[64] Brown DA (1994). Effect of phosphorothioate modification of oligodeoxynucleotides on specific protein binding. J. Biol. Chem..

[65] Stivers JT, Nagarajan R (2006). Probing enzyme phosphoester interactions by combining mutagenesis and chemical modification of phosphate ester oxygens. Chem. Rev..

[66] Eckstein F (2014). Phosphorothioates, essential components of therapeutic oligonucleotides. Nucleic Acid Ther..

[67] Frazier KS (2015). Antisense oligonucleotide therapies: the promise and the challenges from a toxicologic pathologist’s perspective. Toxicol. Pathol..

[68] Liang XH, Sun H, Shen W, Crooke ST (2015). Identification and characterization of intracellular proteins that bind oligonucleotides with phosphorothioate linkages. Nucleic Acids Res..

[69] Shen W (2019). Chemical modification of PS-ASO therapeutics reduces cellular protein-binding and improves the therapeutic index. Nat. Biotechnol..

[70] Iwamoto N (2017). Control of phosphorothioate stereochemistry substantially increases the efficacy of antisense oligonucleotides. Nat. Biotechnol..

[71] Pohar J (2017). Phosphodiester backbone of the CpG motif within immunostimulatory oligodeoxynucleotides augments activation of Toll-like receptor 9. Sci. Rep..

[72] Knouse KW (2018). Unlocking P(V): reagents for chiral phosphorothioate synthesis. Science.

[73] Scheiermann J, Klinman DM (2014). Clinical evaluation of CpG oligonucleotides as adjuvants for vaccines targeting infectious diseases and cancer. Vaccine.

[74] Ohto U (2015). Structural basis of CpG and inhibitory DNA recognition by Toll-like receptor 9. Nature.

[75] Evans P (2006). Scaling and assessment of data quality. Acta Crystallogr. D Biol. Crystallogr..

[76] Kabsch W (2010). X. Acta Crystallogr. D Biol. Crystallogr..

[77] Winn MD (2011). Overview of the CCP4 suite and current developments. Acta Crystallogr. D Biol. Crystallogr..

[78] Winter G (2018). DIALS: implementation and evaluation of a new integration package. Acta Crystallogr. D Struct. Biol..

[79] Karplus PA, Diederichs K (2015). Assessing and maximizing data quality in macromolecular crystallography. Curr. Opin. Struct. Biol..

[80] McCoy AJ (2007). Phaser crystallographic software. J. Appl. Crystallogr..

[81] Adams PD (2010). PHENIX: a comprehensive Python-based system for macromolecular structure solution. Acta Crystallogr. D Biol. Crystallogr..

[82] Emsley P, Cowtan K (2004). Coot: model-building tools for molecular graphics. Acta Crystallogr. D Biol. Crystallogr..

[83] Terwilliger TC (2008). Iterative-build OMIT maps: map improvement by iterative model building and refinement without model bias. Acta Crystallogr. D Biol. Crystallogr..

[84] Demeler, B. & Gorbet, G. Analytical ultracentrifugation data analysis with UltraScan-III. In *Analytical Ultracentrifugation: Instrumentation, Software, and Applications* (eds Uchiyama, S., Stafford, W. F. & Laue, T.) Ch. 8, 119–143 (Springer, 2016).

[85] Brookes E, Cao W, Demeler B (2010). A two-dimensional spectrum analysis for sedimentation velocity experiments of mixtures with heterogeneity in molecular weight and shape. Eur. Biophys. J..

[86] Demeler B (2010). Methods for the design and analysis of sedimentation velocity and sedimentation equilibrium experiments with proteins. Curr. Protoc. Protein Sci..

[87] Cho BC, Hardies SC, Jang GI, Hwang CY (2018). Complete genome of streamlined marine actinobacterium Pontimonas salivibrio strain CL-TW6(T) adapted to coastal planktonic lifestyle. BMC Genomics.

[88] Altschul SF (1997). Gapped BLAST and PSI-BLAST: a new generation of protein database search programs. Nucleic Acids Res..

[89] Soding J, Biegert A, Lupas AN (2005). The HHpred interactive server for protein homology detection and structure prediction. Nucleic Acids Res..

[90] Hughey R, Krogh A (1996). Hidden Markov models for sequence analysis: extension and analysis of the basic method. Comput. Appl. Biosci..

[91] Ronquist F (2012). MrBayes 3.2: efficient Bayesian phylogenetic inference and model choice across a large model space. Syst. Biol..

[92] Diaz-Griffero F (2008). A human TRIM5alpha B30.2/SPRY domain mutant gains the ability to restrict and prematurely uncoat B-tropic murine leukemia virus. Virology.

[93] Marin J, Battistuzzi FU, Brown AC, Hedges SB (2017). The timetree of prokaryotes: new insights into their evolution and speciation. Mol. Biol. Evol..

