# Supplementary material for: Nucleic acid binding by SAMHD1 contributes to the antiretroviral activity and is enhanced by the GpsN modification
Source: Nat Commun. 2021 Feb 2;12:731. doi: 10.1038/s41467-021-21023-8 (PMC7854603; doi:10.1038/s41467-021-21023-8)
Supplement: Supplementary file 1 — Supplementary Information [file 41467_2021_21023_MOESM1_ESM.pdf]

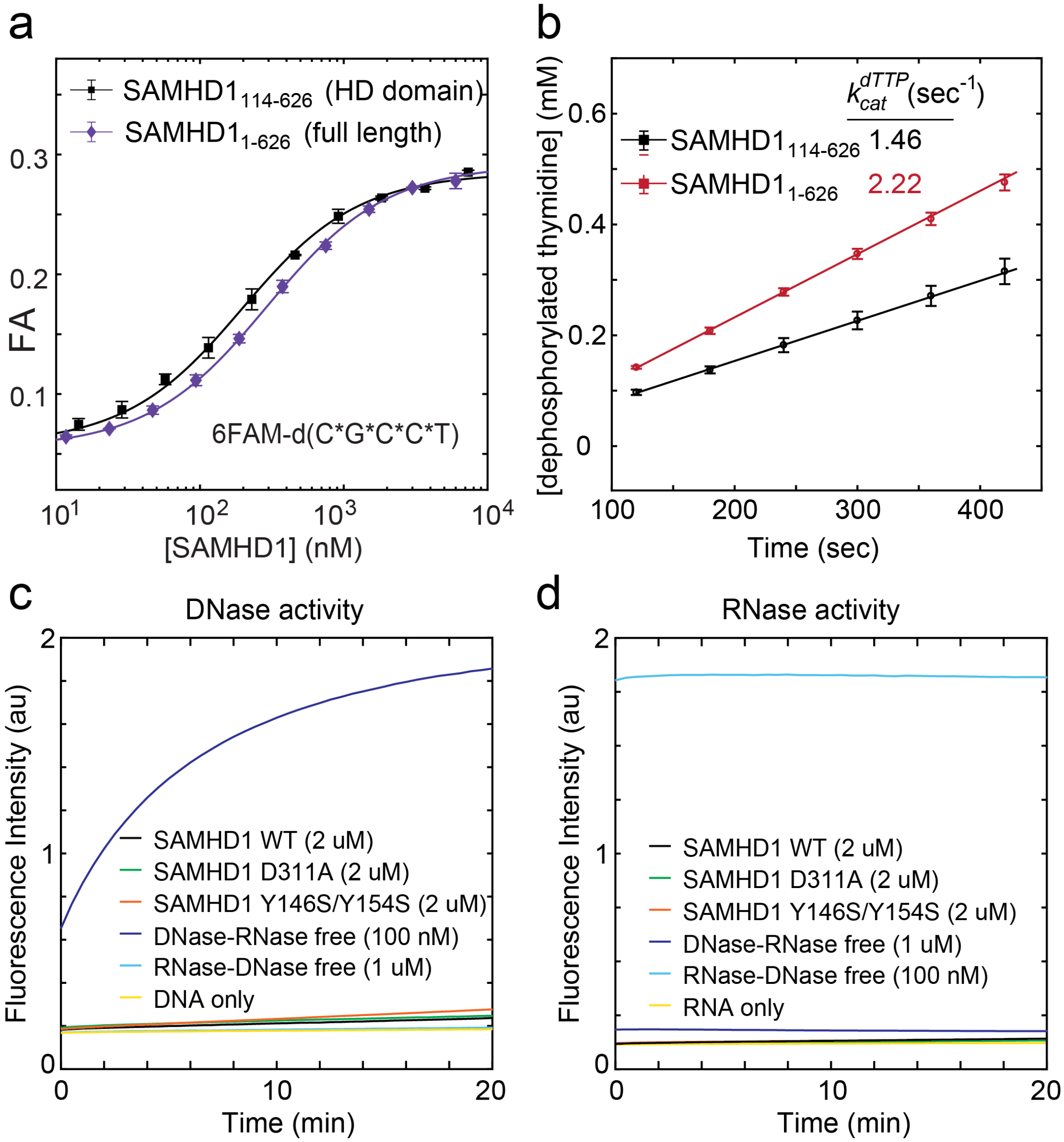

Supplementary Figure 1. Comparison of SAMHD1$_{1\text{-}626}$ and SAMHD1$_{114\text{-}626}$ constructs and evaluation of nuclease contamination. (A) Binding of short phosphorothioated oligonucleotides described in this study was not significantly different for the full-length WT SAMHD1 construct (SAMHD1$_{1\text{-}626}$) and the WT HD-domain construct (SAMHD1$_{114\text{-}626}$), indicating that the N-terminal SAM domain is not required for the interaction (n = 2 independent experiments). (B) The dNTPase activity of the two constructs is also similar (n = 2 independent experiments). (C,D) Nuclease activity is known to co-purify with recombinant SAMHD1 expressed in *E. coli*. This activity was monitored using quenched fluorescent oligonucleotides as previously described in ref. 24. Less than 5% of the 45-base nucleic acid substrates containing 5′-FAM and 3′-BHQ1 labels is cleaved in 20 min. Fluorescence anisotropy measurements reported in this study were performed in less than 2 min after the addition of SAMHD1 to oligonucleotides, so the expected effect of contaminating nuclease activity on these measurements is insignificant (see also Supplementary Fig. 2D). Error bars represent s.d. of two replicate measurements performed on distinct samples. Source data are provided as a Source Data File.

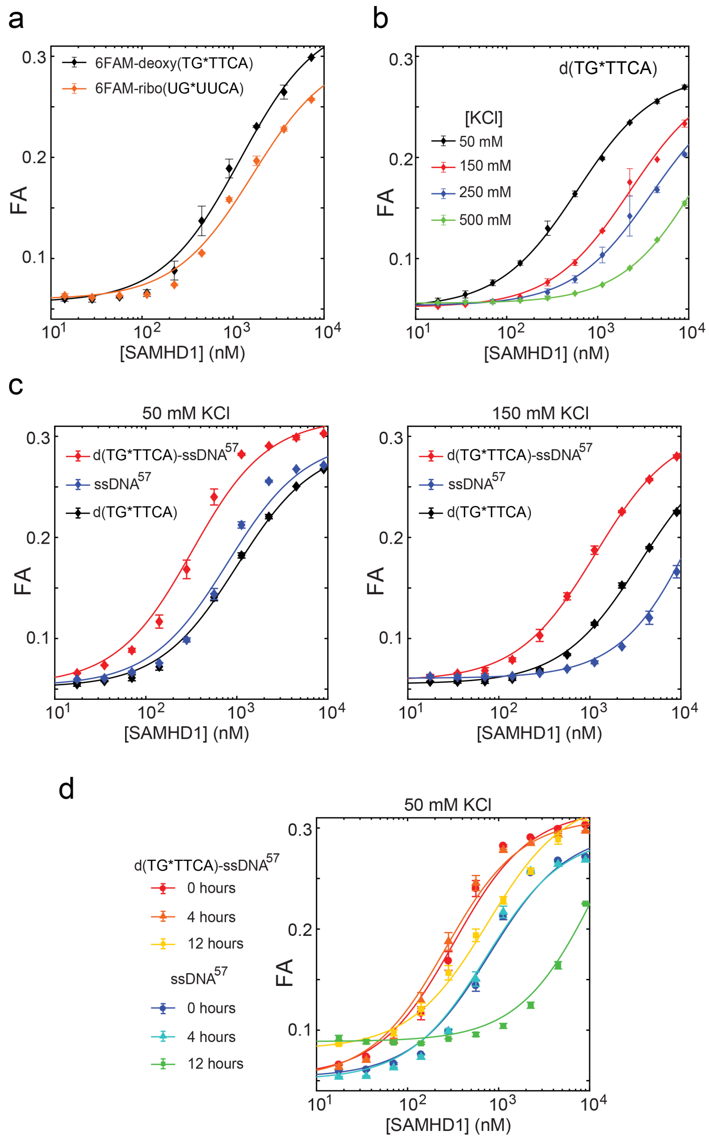

Supplementary Figure 2. Binding of oligonucleotides containing a single phoshphorothioate bond to SAMHD1$_{114\text{-}626}$. (A) Deoxyribo- and ribo-oligonucleotides containing a single GpsN modification bind to SAMHD1 with similar affinities (n = 2 independent experiments). (B) Tolerance of the 6FAM-deoxy(TG*TTCA) oligonucleotide binding affinity to increasing ionic strength of the buffer (n = 2 independent experiments). (C) 6FAM-deoxy(TG*TTCA) oligonucleotide was extended with the sequence of the ssDNA^{57} oligonucleotide (ref. 47) and binding affinity of this longer oligonucleotide was compared to 6FAM-deoxy(TG*TTCA) and 6FAM-ssDNA^{57} monitored using fluorescence anisotropy at two different ionic strengths. At 50 mM KCl the GpsN modification results in a 2-3 fold enhancement of apparent binding affinity, whereas at 150 mM KCl the effect is larger than an order of magnitude, which is in agreement with data shown in Fig. 1AB (n = 2 independent experiments). (D) To further evaluate the potential effect of contaminating nuclease activity on the binding assays, we repeated the 50 mM KCl fluorescence anisotropy measurements at several time increments following the addition of SAMHD1 to fluorescent oligonucleotides. We observe that it takes hours for the binding curves to deteriorate due to the contaminating nuclease activity (n = 2 independent experiments). Error bars represent s.d. of two replicate measurements performed on distinct samples. Source data are provided as a Source Data File.

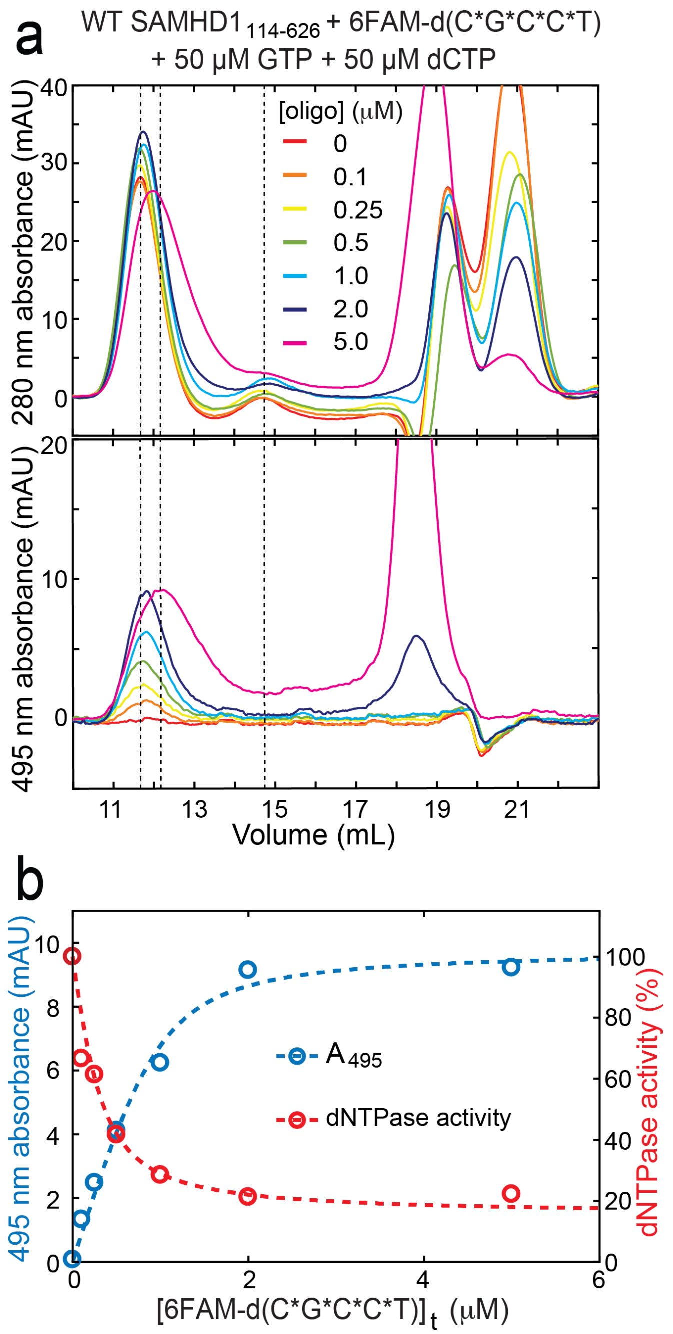

Supplementary Figure 3. Effect of oligonucleotide binding on the dNTPase activity of SAMHD1. (A) The same oligonucleotide titration experiment as in Fig. 4B but performed with the catalytically active WT SAMHD1$_{114\text{-}626}$. (B) The maximum A_{495} absorbance of the SAMHD1-associated oligonucleotide peak (blue) and the relative dTTP hydrolysis rate (red) plotted against the total concentration of oligonucleotide incubated with SAMHD1 prior to the SEC run. The dNTPase activity of the protein eluting at the 12 mL retention volume was measured immediately following the SEC run.

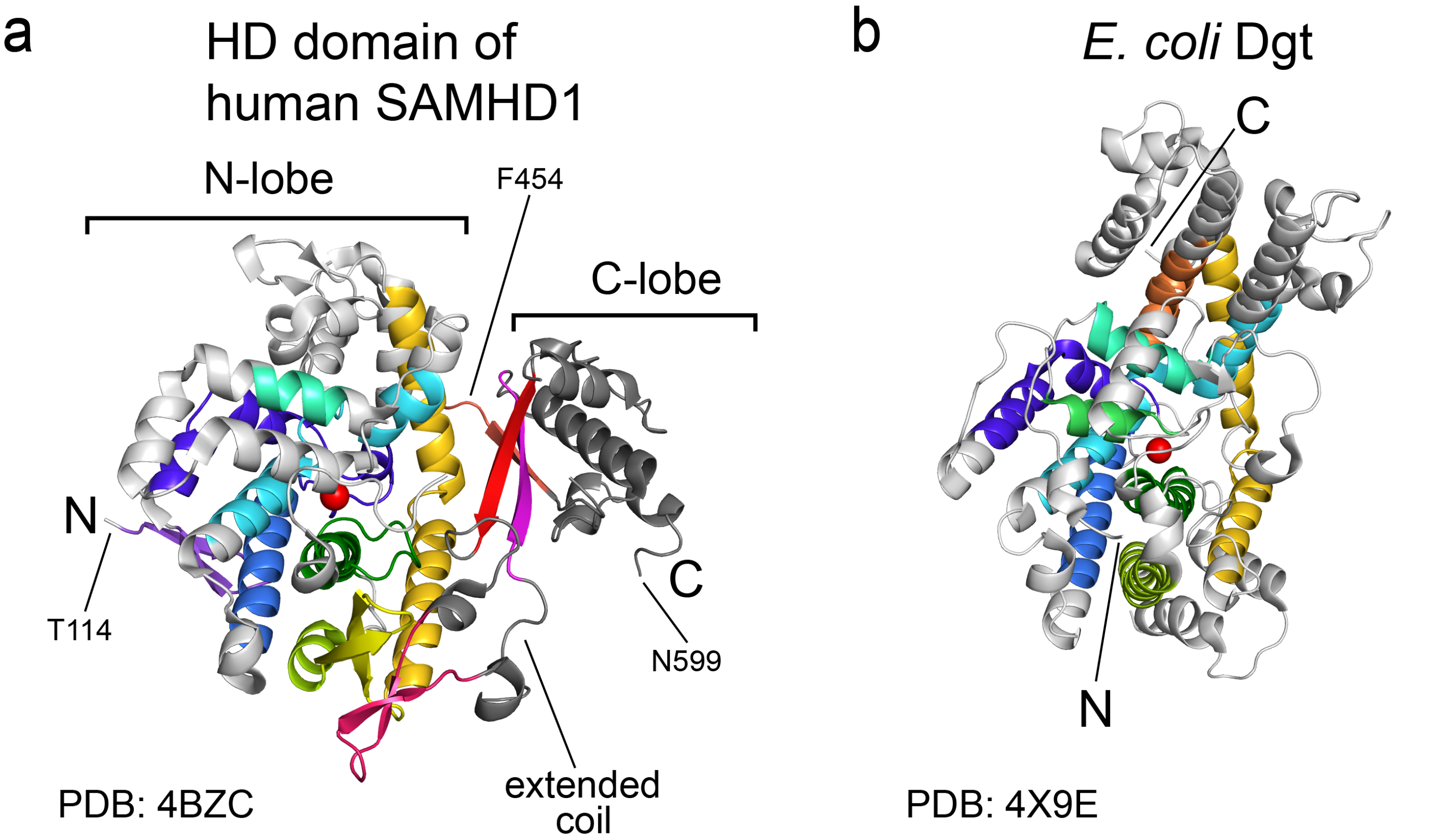

Supplementary Figure 4. Structural comparison of the HD domain of SAMHD1 and *E.coli* Dgt. (A)The HD domain of SAMHD1 consists of a larger N-terminal lobe (N-lobe, residues 114-453, light grey) and a smaller C-terminal lobe (C-lobe, residues 454-600, dark grey). The C-terminal segment of SAMHD1 (residues 600-626) is not observed in the crystal structures of SAMHD1 and is thought to be unstructured. The N-lobe contains the catalytic site of the enzyme, whereas the C-lobe contributes to the allosteric activation of the enzyme. A conserved extended coil segment emanates from the C-lobe, spans the length of the N-lobe and contributes residues to the allosteric binding sites and the tetramerization interface. This domain architecture is conserved in the SAMHD1-like family of prokaryotic dNTPases. Conserved secondary structure elements in the SAMHD1-like dNTPase family are color coded the same way as in the Fig. 5AC. Their pairwise alignment with other known structures of SAMHD1-like dNTPases yields Cα RMSD < 1.8 Å. Secondary structure elements shown in grey are less well conserved. (B) Dgt and other members of the Dgt-like family of prokaryotic dNTPases have the same active site architecture as SAMHD1-like dNTPases, but there are also some notable structural differences between the two families. Dgt-like dNTPases lack the C-lobe, and two beta hairpin elements conserved in the SAMHD1 family are not present in their catalytic lobes (Fig. 5C). Conserved secondary structure elements (Cα RMSD < 1.8 Å) in known Dgt-like structures are also color coded as in the Fig. 5AC.

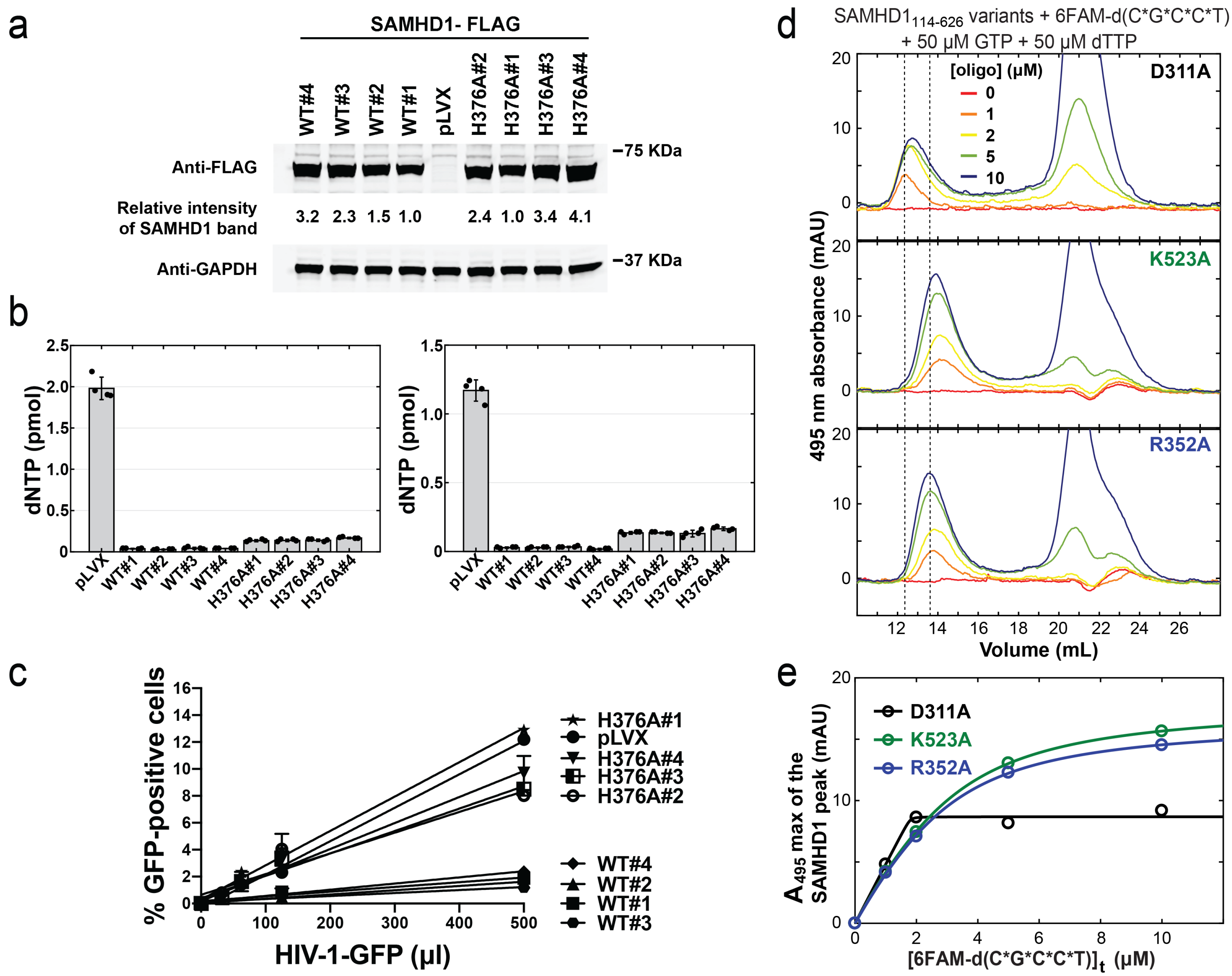

Supplementary Figure 5. Characterization of the H376A, R352A and K523A mutations. (A) Distinct stably transduced cell lines express SAMHD1 WT and H376A variants at different levels as quantified by fluorescent WB (n = 3 independent experiments). (B) Comparison of dNTP depletion after PMA-induced differentiation in distinct U937 cell lines expressing different amounts of SAMHD1 variants (n = 4 independent experiments). (C) Comparison of HIV restriction by distinct U937 cell lines expressing different amounts of SAMHD1 variants (n = 2 independent experiments). (D,E) Oligonucleotide binding stoichiometry by D311A, R352A and K523A variants in the presence of 50 µM GTP and 50 µM dTTP. Experiments were performed the same way as experiments in Fig. 4B and Fig. S3. R352A and K523A variants display a 2-fold increase in the oligonucleotide binding stoichiometry compared to D311A. This observation suggests that although R352A and K523A mutations do not affect oligonucleotide binding and oligonucleotide-dependent dimerization, they abolish the ability of the protein to form mixed-occupancy tetramers with 1:4 oligo:SAMHD1 stoichiometry in the presence of GTP/dNTP.

Primer Name	Sequence
H376A	5′ – CGCCGCGCTTACCAAGCAAAAGTAGGAAATATC –3′
K523A	5′ – GTTCTATTGCGCAACCGCTCCGAATCGCGC – 3′
R352A	5′ – CCGTATCTGTGCAGCAGATAAAGAAGTAGGGAATCTCTATG – 3′

Supplementary Table 1. Primer sequences for site-directed mutagenesis of SAMHD1.
