# Supplementary material for: Nucleic acid binding by SAMHD1 contributes to the antiretroviral activity and is enhanced by the GpsN modification
Source: Nat Commun. 2021 Feb 2;12:731. doi: 10.1038/s41467-021-21023-8 (PMC7854603; doi:10.1038/s41467-021-21023-8)
Supplement: Supplementary file 2 — Reporting Summary [file 41467_2021_21023_MOESM2_ESM.pdf]

nature research

Corresponding author(s): Dmitri Ivanov

Last updated by author(s): December 23, 2020

Reporting Summary

Nature Research wishes to improve the reproducibility of the work that we publish. This form provides structure for consistency and transparency in reporting. For further information on Nature Research policies, see Authors & Referees and the Editorial Policy Checklist .

Statistics

For all statistical analyses, confirm that the following items are present in the figure legend, table legend, main text, or Methods section.

n/a	Confirmed	
☐	☒	The exact sample size (*n*) for each experimental group/condition, given as a discrete number and unit of measurement
☐	☒	A statement on whether measurements were taken from distinct samples or whether the same sample was measured repeatedly
☒	☐	The statistical test(s) used AND whether they are one- or two-sided<br>*Only common tests should be described solely by name; describe more complex techniques in the Methods section.*
☒	☐	A description of all covariates tested
☒	☐	A description of any assumptions or corrections, such as tests of normality and adjustment for multiple comparisons
☒	☐	A full description of the statistical parameters including central tendency (e.g. means) or other basic estimates (e.g. regression coefficient) AND variation (e.g. standard deviation) or associated estimates of uncertainty (e.g. confidence intervals)
☒	☐	For null hypothesis testing, the test statistic (e.g. *F*, *t*, *r*) with confidence intervals, effect sizes, degrees of freedom and *P* value noted<br>*Give P values as exact values whenever suitable.*
☒	☐	For Bayesian analysis, information on the choice of priors and Markov chain Monte Carlo settings
☒	☐	For hierarchical and complex designs, identification of the appropriate level for tests and full reporting of outcomes
☒	☐	Estimates of effect sizes (e.g. Cohen's *d*, Pearson's *r*), indicating how they were calculated

Our web collection on statistics for biologists contains articles on many of the points above.

Software and code

Policy information about availability of computer code

Data collection	To collect the fluorescence polarization data, we used Biotek Gen5 version 2.09.<br>To collect the crystal structure data, we used the PHASER 2.8.2 crystallographic software.<br>To collect the NMR data, we used Bruker Topspin 3.2.<br>To collect the size-exclusion chromatography data, we used Biorad ChromLab 6.0.
Data analysis	MATLAB R2016B (Mathworks) was used to analyze the fluorescence polarization, NMR dNTPase and oligonucleotide stereospecificity, and size-exclusion chromatography data.<br>Ultrascan III was used to analyze the analytical ultracentrifugation data.<br>PYMOL 1.8.7.0 was used to analyze the crystal structures.<br>To refine the crystal structures, PHENIX 1.15.2 and COOT 0.8.9.2 was used.<br>To quantify the primer products on a gel, QuantityOne 4.6.8 software was used.<br>To analyze sequence alignment data, MrBayes 3.2.7 was used.

For manuscripts utilizing custom algorithms or software that are central to the research but not yet described in published literature, software must be made available to editors/reviewers. We strongly encourage code deposition in a community repository (e.g. GitHub). See the Nature Research guidelines for submitting code & software for further information.

Data

Policy information about availability of data

All manuscripts must include a data availability statement. This statement should provide the following information, where applicable:

- Accession codes, unique identifiers, or web links for publicly available datasets
- A list of figures that have associated raw data
- A description of any restrictions on data availability

Data supporting the findings of this paper are available from the corresponding authors upon reasonable request. Atomic coordinates and structure factors have

been deposited in the Protein Data Bank under accession codes PDB 6U6Y(https://www.rcsb.org/structure/6U6Y) [ribo(CGCCU)], PDB 6U6X (https://www.rcsb.org/structure/6U6X) [deoxy(C*G*C*C*T), and PDB 6U6Z (https://www.rcsb.org/structure/6U6Z) [deoxy(TG*TTCA)]. Source data are provided with this paper.

Field-specific reporting

Please select the one below that is the best fit for your research. If you are not sure, read the appropriate sections before making your selection.

[x] Life sciences   [ ] Behavioural & social sciences   [ ] Ecological, evolutionary & environmental sciences

For a reference copy of the document with all sections, see nature.com/documents/nr-reporting-summary-flat.pdf

Life sciences study design

All studies must disclose on these points even when the disclosure is negative.

Sample size	No sample size calculation was performed. The sample size was chosen on the basis of previous experience with each specific assay. Most assays reveal significant differences with only samples per measurement and are highly reproducible when experiments are repeated on multiple occasions.
Data exclusions	No data was excluded from this study.
Replication	To verify the reproducibility of our data, we have done duplicates of our measurements. Multiple biophysical techniques under the same conditions produced the same findings. All attempts at replication were successful.
Randomization	No randomization was performed. Covariance are not relevant for experiments using cell lines.
Blinding	Investigators were blinded to group allocation for measurements of cellular dNTP levels. Blinding was not possible for all other experiments because sample prepararation and sample measurements were performed by the same individual.

Reporting for specific materials, systems and methods

We require information from authors about some types of materials, experimental systems and methods used in many studies. Here, indicate whether each material, system or method listed is relevant to your study. If you are not sure if a list item applies to your research, read the appropriate section before selecting a response.

Materials & experimental systems

n/a	Involved in the study	
[ ]	[x]	Antibodies
[ ]	[x]	Eukaryotic cell lines
[x]	[ ]	Palaeontology
[x]	[ ]	Animals and other organisms
[x]	[ ]	Human research participants
[x]	[ ]	Clinical data

Methods

n/a	Involved in the study	
[x]	[ ]	ChIP-seq
[x]	[ ]	Flow cytometry
[x]	[ ]	MRI-based neuroimaging

Antibodies

Antibodies used	Monoclonal Anti-FLAG ® M2 antibody; Supplier: SIGMA-ALDRICH; LOT #: SLBM0089V; CATALOG #: F1804-5MG<br>Mouse monoclonal anti-glyceraldehyde-3-phosphate dehydrogenase (GAPDH); Supplier: Invitrogen; CATALOG #: AM4300
Validation	These are very widely used antibodies, described in more than 4000 peer reviewed publications. More details regarding manufacturer's validation procedures can be found here: https://www.sigmaaldrich.com/catalog/product/sigma/f1804?lang=en&region=US and https://www.thermofisher.com/order/genome-database/details/antibody/AM4300.html. In this study cells transfected with the empty pLVX vector used as negative controls displayed no significant staining with the antibody.

Eukaryotic cell lines

Policy information about cell lines

Cell line source(s)	Human HEK29T/17 cells (ATCC #: CRL-11268); Human U937 cells (ATCC #: CRL-1593.2); Dog Cf2Th cells (ATCC #: CRL-1430) all cells were ordered from ATCC.org
Authentication	Cells were authenticated by ATCC and Certificate of Analysis was provided when cells were ordered. Cells used in the experiments described in the study were propagated from the original ATCC stocks. No additional authentication was performed.
Mycoplasma contamination	Cells tested negative for mycoplasma contamination using the Universal Mycoplasma Detection Kit ordered from ATCC.
Commonly misidentified lines (See ICLAC register)	No commonly misidentified cell lines were used in this study.
