# Supplementary material for: Nucleic acid binding by SAMHD1 contributes to the antiretroviral activity and is enhanced by the GpsN modification
Source: Nat Commun. 2021 Feb 2;12:731. doi: 10.1038/s41467-021-21023-8 (PMC7854603; doi:10.1038/s41467-021-21023-8)
Supplement: Supplementary file 3 — Source Data [file 41467_2021_21023_MOESM3_ESM.pdf]

Figure 1a Fluorescence polarization (C*G*C*C*T vs ssDNA57)

F*DNA1	50 mM KCl	378.5	378	369.5	363.5	348.5	324.5	287
		0.707107	0	0.707107	0.707107	0.707107	0.707107	15.55635
	150 mM KCl	372.5	365.5	358.5	345.5	336	279	224.5
		0.707107	0.707107	0.707107	2.12132	19.79899	2.828427	10.6066
	250 mM KCl	371	364.5	352	333	305.5	259	208.5
		1.414214	2.12132	0	1.414214	0.707107	7.071068	3.535534
	500 mM KCl	364	347.5	328.5	303.5	268.5	215.5	165.5
		2.828427	2.12132	2.12132	2.12132	3.535534	0.707107	2.12132
ssDNA57	50 mM KCl	354.5	355.5	354	350	331.5	252.5	184
		2.12132	2.12132	1.414214	0	3.535534	14.84924	4.242641
	150 mM KCl	305	224.5	166.5	127.5	109	97	91.5
		1.414214	4.949747	6.363961	2.12132	2.828427	1.414214	0.707107
	250 mM KCl	189	129.5	106	98.5	96.5	92.5	107.5
		0	10.6066	0	2.12132	2.12132	0.707107	19.09188
	500 mM KCl	119	116	117.5	116.5	114	111.5	111.5
		1.414214	1.414214	3.535534	2.12132	1.414214	3.535534	2.12132
	[prot] (nM)	18150	9075	4537.5	2268.75	1134.375	567.1875	283.5938

219.5	156.5	118.5	101	89.5	100	80	76
19.09188309	14.84924	9.192388	0	0.707107	0	1.414214	1.414214
162.5	121.5	99	86	90	78.5	77	83
7.778174593	3.535534	4.242641	2.828427	12.72792	2.12132	1.414214	9.899495
155.5	120	99	87	81.5	80	80.5	77.5
2.121320344	1.414214	1.414214	0	0.707107	1.414214	0.707107	0.707107
130	104.5	90.5	85	82	80	79.5	78.5
2.828427125	2.12132	3.535534	2.828427	4.242641	7.071068	0.707107	3.535534
132.5	108	92.5	85	81.5	79	78.5	76
0.707106781	2.828427	2.12132	0	0.707107	0	0.707107	1.414214
100.5	89.5	86	85	85.5	93	85.5	85
13.43502884	0.707107	1.414214	0	0.707107	11.31371	0.707107	1.414214
93	92	92	92	91.5	90.5	104.5	91.5
2.828427125	2.828427	2.828427	2.828427	2.12132	0.707107	13.43503	2.12132
110.5	109	110	109.5	110.5	118	109.5	109.5
0.707106781	2.828427	1.414214	2.12132	3.535534	16.97056	0.707107	0.707107
141.796875	70.89844	35.44922	17.72461	8.862305	4.431152	2.215576	1.107788

74.5	75.5	76.5	75	75.5
0.707107	0.707107	0.707107	2.828427	2.12132
74	76	87.5	79	75.5
0	0	19.09188	7.071068	0.707107
77.5	78	77.5	77.5	78
0.707107	0	0.707107	0.707107	0
79	80	79	78	82
1.414214	0	1.414214	1.414214	1.414214
79.5	77.5	77.5	77.5	79.5
0.707107	0.707107	0.707107	2.12132	2.12132
86.5	85.5	85.5	85	91
0.707107	0.707107	2.12132	2.828427	5.656854
92	93.5	93	89.5	91
1.414214	0.707107	1.414214	2.12132	1.414214
108.5	107	108.5	106	109
2.12132	2.828427	2.12132	5.656854	4.242641
0.553894	0.276947	0.138474	0.069237	0.034618

Figure 1b Fluorescence polarization (C*G*C*C*U vs ssRNA40)

F*RNA1	50 mM KCl	363	360	352	342.5
		2.82842713	1.41421356	1.41421356	0.70710678
	150 mM KCl	356	343	331	307.5
		1.41421356	5.65685425	1.41421356	0.70710678
	250 mM KCl	349.5	330	305.5	272
		0.70710678	2.82842713	3.53553391	1.41421356
	500 mM KCl	331	292	261	220
		2.82842713	11.3137085	1.41421356	5.65685425
ssRNA40	50 mM KCl	255	250.5	244	220
		0	2.12132034	1.41421356	7.07106781
	150 mM KCl	225.5	175	134	104
		0.70710678	9.89949494	2.82842713	4.24264069
	250 mM KCl	140	99	94.5	88.5
		4.24264069	1.41421356	2.12132034	3.53553391
	500 mM KCl	100.5	100	98	97
		2.12132034	2.82842713	4.24264069	2.82842713
	[prot] (nM)	18150	9075	4537.5	2268.75

325.5	295.5	255	202.5	155	118.5	96
7.77817459	13.4350288	16.9705628	26.1629509	18.3847763	10.6066017	1.41421356
270.5	224.5	177.5	138.5	110	91.5	81
2.12132034	12.0208153	12.0208153	13.4350288	8.48528137	4.94974747	1.41421356
236	191	147	116.5	96.5	83.5	80
5.65685425	7.07106781	5.65685425	3.53553391	2.12132034	2.12132034	1.41421356
180.5	140	109	95	87.5	77.5	79.5
12.0208153	12.7279221	8.48528137	2.82842713	0.70710678	0.70710678	3.53553391
191	145	110.5	95	88	81.5	80.5
7.07106781	9.89949494	3.53553391	4.24264069	2.82842713	0.70710678	0.70710678
94	89	84.5	84	83	81	80.5
1.41421356	2.82842713	0.70710678	0	1.41421356	0	0.70710678
87.5	86	85.5	83.5	83	82.5	82
2.12132034	1.41421356	0.70710678	2.12132034	1.41421356	0.70710678	1.41421356
95	90.5	92.5	91.5	90	90	91.5
2.82842713	3.53553391	2.12132034	2.12132034	1.41421356	2.82842713	2.12132034
1134.375	567.1875	283.59375	141.796875	70.8984375	35.4492188	17.7246094

85	78.5	76.5	74.5	71.5	71.5	72
2.82842713	2.12132034	0.70710678	0.70710678	2.12132034	0.70710678	1.41421356
82.5	74	73	72.5	72	72	73
9.19238816	0	0	0.70710678	1.41421356	0	1.41421356
78	75	75	74	74	75.5	75
1.41421356	0	0	1.41421356	2.82842713	0.70710678	0
77	75.5	75	76.5	76.5	76.5	75
4.24264069	2.12132034	0	3.53553391	4.94974747	2.12132034	2.82842713
78.5	77.5	77.5	77.5	78.5	78	78
0.70710678	0.70710678	0.70710678	0.70710678	0.70710678	1.41421356	0
82	82	80.5	82.5	81.5	80	83
1.41421356	0	0.70710678	0.70710678	2.12132034	1.41421356	2.82842713
85	80.5	81.5	81.5	81	94	87.5
7.07106781	2.12132034	3.53553391	0.70710678	1.41421356	16.9705628	6.36396103
89	89	89.5	91	90	90.5	90
4.24264069	1.41421356	0.70710678	0	2.82842713	4.94974747	2.82842713
8.86230469	4.43115234	2.21557617	1.10778809	0.55389404	0.27694702	0.13847351

76	83
7.07106781	8.48528137
72.5	103.5
0.70710678	43.1335137
75	76
1.41421356	1.41421356
74.5	82.5
3.53553391	9.19238816
78	80.5
1.41421356	0.70710678
80.5	98
0.70710678	18.3847763
80.5	82
2.12132034	2.82842713
88	91.5
5.65685425	3.53553391
0.06923676	0.03461838

Figure 1c Fluorescence polarization of mutants

Column No.	Conc(WT) nM	FstarDNA-Pol	FRNA-Pol	Conc(D311A)	FstarDNA-Pol
1	7647	348	291	45441	372
2	3823.5	332	233	22720.5	355
3	1911.75	321	172	11360.25	346
4	955.875	308	126	5680.125	342
5	477.9375	286	102	2840.0625	335
6	238.96875	258	86	1420.03125	326
7	119.484375	217	79	710.015625	312
8	59.7421875	169	80	355.007813	288
9	29.8710938	132	77	177.503906	244
10	14.9355469	105	76	88.7519531	186
11	7.46777344	92	75	44.3759766	138
12	3.73388672	82	76	22.1879883	110
13	1.86694336	80	76	11.0939941	92
14	0.93347168	80	76	5.54699707	85
15	0.46673584	79	77	2.77349854	80
16	0.23336792	77	77	1.38674927	79
17	0.11668396	79	75	0.69337463	77
18	0.05834198	77	75	0.34668732	76

FRNA-Pol	Conc(T592D)nM	FstarDNA-Pol	FRNA-Pol	Conc(YY) nM	FDNA-Pol	FRNA-Pol
391	4044	347	203	6066	92	75
377	2022	323	147	3033	88	76
355	1011	304	110	1516.5	85	78
315	505.5	280	91	758.25	85	77
262	252.75	247	83	379.125	84	76
198	126.375	204	77	189.5625	83	73
146	63.1875	159	75	94.78125	81	72
114	31.59375	125	76	47.390625	82	75
95	15.796875	102	74	23.6953125	81	74
85	7.8984375	90	75	11.8476563	79	73
80	3.94921875	84	75	5.92382813	77	74
78	1.974609375	83	74	2.96191406	80	73
77	0.987304688	80	75	1.48095703	78	72
76	0.493652344	81	75	0.74047852	78	73
76	0.246826172	80	74	0.37023926	78	72
76	0.123413086	80	74	0.18511963	80	72
76	0.061706543	81	72	0.09255982	79	72
74	0.030853272	78	73	0.04627991	78	70

Conc(SAMHD1wt)nM	FDNA1RegBor	FDNA1StarBonds
41000	263	358
20500	275	358
10250	262	357
5125	231	355
2562.5	186	351
1281.25	136	337
640.625	100	320
320.3125	85	286
160.15625	84	227
80.078125	72	168
40.0390625	73	125
20.01953125	70	99
10.00976563	73	90
5.004882813	68	84
2.502441406	71	82
1.251220703	70	79
0.625610352	75	78
0.312805176	71	79

Figure 2I FloPol (G base substitution)

[SAMHD1 WT	<F*DNA1>	Stdev(F*DNA1	<F*DNA2>	Stdev(F*DNA2	<F*DNA3>
30661.7647	353.333333	6.35085296	198.333333	1.52752523	167.333333
15330.8824	349	3.60555128	164.666667	3.05505046	135
7665.44118	344.666667	0.57735027	137.333333	2.30940108	111.333333
3832.72059	337	3	114	1	97.3333333
1916.36029	327.333333	3.05505046	97.6666667	1.15470054	89
958.180147	315.666667	3.51188458	86.6666667	0.57735027	83
479.090074	298.666667	4.04145188	81	1	79
239.545037	264	11.7898261	80.6666667	0.57735027	80
119.772518	220.333333	12.6622799	77.6666667	1.52752523	78.6666667
59.8862592	175.333333	11.0151411	76	1.73205081	76
29.9431296	136	5.19615242	76.6666667	1.52752523	76.3333333
14.9715648	112	4.35889894	76.3333333	1.15470054	76.3333333
7.4857824	97	3.60555128	76.6666667	1.15470054	76.3333333
3.7428912	89.6666667	3.05505046	76.3333333	1.52752523	77.3333333
1.8714456	84.3333333	1.52752523	76.3333333	1.52752523	76
0.9357228	80.3333333	1.15470054	75.6666667	1.52752523	76.3333333
0.4678614	79.6666667	2.51661148	75.3333333	1.15470054	75.3333333
0.2339307	79	1.73205081	74	1	74

Stdev(F*DNA3	<FRNA1>	Stdev(FRNA1)	<FRNA1x1>	Stdev(FRNA1x1)
3.05505046	360	1	409.666667	0.57735027
3.46410162	350	3.60555128	407.666667	1.15470054
2.081666	325.333333	2.081666	398	1
2.88675135	285.666667	1.52752523	380.666667	0.57735027
4.35889894	234	2	356	1
1.73205081	173.333333	3.05505046	319	3.46410162
2.64575131	124.333333	2.081666	261.333333	7.3711148
1.73205081	95.3333333	2.88675135	174.666667	11.06044
1.52752523	81.3333333	1.15470054	109	8.88819442
1	76.3333333	1.52752523	85.3333333	4.93288286
1.52752523	76.3333333	2.51661148	78.6666667	2.081666
2.081666	75.6666667	1.15470054	73.3333333	1.52752523
1.15470054	75	1	71.6666667	0.57735027
1.52752523	76.6666667	2.51661148	70	1
2	74.6666667	3.7859389	70.6666667	0.57735027
2.88675135	76.3333333	2.081666	68.6666667	0.57735027
0.57735027	75	3	68	1.73205081
1.73205081	75.3333333	1.52752523	70	1

Figure 2J dNTPase vs Oligos

[DNA1] uM	<DNA1-Rate>	Std(DNA1-Rat	[RNA1] uM	<RNA1-Rate>
0	3.24652525	0.07328603	0	3.3647215
1	3.4554376	0.03908377	1	3.21714955
2	3.0665297	0.2155789	2	2.8324235
5	2.9497512	0.3459981	5	2.4230146
10	3.3737073	0.4325194	10	1.798028
20	3.0493332	0.31711547	20	1.7044532
50	3.10023595	0.22279853	50	1.7026268
100	2.565203	0.14422009	100	1.3773563

Std(RNA1-Rat	[starDNA1] u	<starDNA1-Ra	Std(starDNA1-	[starDNA2] u	<starDNA2-Ra	Std(starDNA2-
0.35818734	0	2.32253105	0.00561011	0	3.4896413	0.13469833
0.18476849	0.1	2.66694385	0.49688825	10	3.2524383	0.09613145
0.11950161	0.2	2.8267614	0.14307075	20	3.0848919	0.48488052
0.41280427	0.5	2.6579078	0.00796952	50	3.06594515	0.32985959
0.1287691	1	2.21021765	0.01209966	100	3.01515805	0.29471171
0.05552613	2	1.9565546	0.09546366			
0.01914308	5	1.29631225	0.59972123			
0.16633414	10	0.75680479	0.31841359			
	20	0.56400764	0.13292676			
	50	0.29671805	0.05857541			

[starDNA3] uM	<starDNA3-Ra	Std(starDNA3-Rate)
0	3.4896413	0.13469833
10	3.7351815	0.25589968
20	3.56668485	0.01738302
50	3.0883654	0.08294886
100	3.2504761	0.25925603

Figure 2K (dNTPase competition)

[GTP] (uM)	0uM DNA		10uM DNA	
1	0.0608	0.03394113	-0.0288	0.03691097
5	0.635	0.09630794	0.1037	0.05656854
10	1.03705	0.04348707	0.19395	0.00898026
25	1.54385	0.10740952	0.72105	0.01817264
50	1.7935	0.13859293	0.98845	0.19919198
100	1.8696	0.03903229	1.6739	0.02856711
200	1.77965	0.04617407	1.29655	0.04136575

2uM DNA

-0.1552	0.13138044
0.0161	0.06109403
0.08635	0.06710443
0.33415	0.10599531
0.6654	0.0311127
1.1129	0.24946727
1.3028	0.21665752

Figure 2L Fluorescence polarization (oligo competition)

no GTP	277	289.5	281.5	250	206.5
	9.899	0.707	6.364	5.657	2.121
0.1 mM dNTP	221	216.5	189	161.5	134
	1.41421356	0.70710678	2.82842713	0.70710678	1.41421356
0.25 mM dNTI	182.5	175.5	146	119	103.5
	0.707	4.95	2.828	0	0.707
0.5 mM dNTP	155.5	141.5	117	103	96
	4.95	7.778	1.414	1.414	1.414
0.75 mM dNTI	142.5	125.5	107	97.5	92.5
	2.121	7.778	2.828	3.536	2.121
1 mM dNTP	133	118.5	102	94.5	90
	7.071	9.192	2.828	2.121	4.243
[prot] (nM)	9000	4500	2250	1125	562.5

163.5	129.5	110	98.5	91.5	87.5	92
4.95	4.95	7.071	4.95	3.536	3.536	11.314
113.5	101.5	94.5	90	87.5	88	86
0.70710678	0.70710678	0.70710678	0	0.70710678	1.41421356	1.41421356
94	87.5	85.5	83.5	83.5	82	82
0	0.707	0.707	0.707	0.707	0	1.414
91	87.5	86	87	85.5	84.5	84
1.414	3.536	2.828	1.414	2.121	3.536	1.414
90	87.5	85.5	87	87	85.5	84.5
0	0.707	0.707	0	2.828	0.707	2.121
87.5	86.5	86.5	85	85	83	84.5
3.536	2.121	2.121	2.828	1.414	1.414	3.536
281.25	140.625	70.313	35.156	17.578	8.789	4.395

85.5	85.5	84	81.5	85	83	84.5
3.536	2.121	1.414	0.707	1.414	2.828	0.707
87	85.5	87	86.5	86	84.5	87.5
0	0.70710678	1.41421356	0.70710678	0	2.12132034	0.70710678
82.5	81.5	82.5	81.5	81	82.5	83.5
0.707	0.707	0.707	0.707	0	0.707	0.707
84.5	85	84	84	82	83	84.5
2.121	2.828	1.414	1.414	0	0	0.707
85	84	85	86	84.5	84	86
0	1.414	1.414	0	0.707	1.414	1.414
83	84.5	83	83.5	83.5	85.5	84
1.414	2.121	2.828	3.536	3.536	2.121	1.414
2.197	1.099	0.549	0.275	0.137	0.069	0.034

Figure 3a G base location

[SAMHD1](nM	FPS006PK1	STDEV(FPS006	FPS006PK2	STDEV(FPS006
7250	390	0	247.5	0.707
3625	350.5	9.192	161.5	12.021
1812.5	310	1.414	134.5	3.536
906.25	259	12.728	115.5	0.707
453.125	192.5	20.506	100	2.828
226.563	126.5	13.435	91	2.828
113.281	95.5	4.95	85	0
56.641	90	1.414	84.5	0.707
28.32	87	4.243	83.5	0.707
14.16	87.5	3.536	83.5	0.707
7.08	88	1.414	83	1.414
3.54	87	2.828	83.5	0.707
1.77	88.5	0.707	83	0
0.885	86.5	2.121	82.5	0.707
0.443	86.5	0.707	83.5	0.707
0.221	86.5	0.707	83.5	0.707
0.111	86.5	0.707	81.5	0.707
0.055	86	4.243	81.5	2.121
0.028	86	1.414	83.5	2.121
0.014	85.5	2.121	84.5	0.707

[SAMHD1](nM	FPS010PK1	STDEV(FPS010	[SAMHD1](nM	FPS006_CYU1	STDEV(FPS006	[SAMHD1](nM
7650	74.5	0.707	8350	109	0	8350
3825	76	1.414	4175	100	2.828	4175
1912.5	79	0	2087.5	98	2.828	2087.5
956.25	82	1.414	1043.75	95	1.414	1043.75
478.125	82	1.414	521.875	91.5	2.121	521.875
239.0625	83	0	260.938	88	2.828	260.938
119.53125	81	2.828	130.469	87	2.828	130.469
59.76563	79.5	3.536	65.234	86.5	2.121	65.234
29.88281	80	2.828	32.617	84.5	0.707	32.617
14.94141	82	2.828	16.309	86.5	0.707	16.309
7.4707	82.5	2.121	8.154	86.5	0.707	8.154
3.73535	82	2.828	4.077	83.5	0.707	4.077
1.86768	81.5	0.707	2.039	84.5	0.707	2.039
0.93384	81	0	1.019	86	1.414	1.019
0.46692	82	0	0.51	84	0	0.51
0.23346	81.5	0.707	0.255	86.5	0.707	0.255
0.11673	81	0	0.127	84	1.414	0.127
0.05836	80.5	2.121	0.064	83	2.828	0.064
0.02918	79.5	0.707	0.032	84	1.414	0.032
0.01459	82.5	0.707	0.016	85	2.828	0.016

FPS006_CYU2	STDEV(FPS006_CYU2)
103.5	0.707
94	0
93	1.414
91	0
89.5	0.707
89.5	0.707
88	1.414
86	0
85.5	0.707
87	1.414
87	1.414
86.5	2.121
87	1.414
85.5	0.707
86.5	0.707
85.5	0.707
86	1.414
85	0
84	1.414
86	1.414

Figure 4C

```
%DNA only titration: taking 495 nm absorbance integral of the
%protein-associated peak (12-14 mL)
x1 = [0.5 0.75 1 2 3 4 5];
y1 = [4.8 6.4 8.3 33.2 43.5 44.8 43.5];


%now we normalize to total protein
y2 = y1*68.8/(82*58.2);

%DNA-dNTP titration; taking 495 nm absorbance integral of the
%protein-associated peak (11.27-13.27 mL)
x3 = [0.25 0.5 1 1.875 3.75 7.5];
y3 = [3.48 6.99 12.56 15.99 21.6 21.06];

%now we normalize to total protein
y4 = y3*68.8/(82*58.2);
```

Figure 6a FloPol of mutants

[SAMHD1](nM	WT	STDEV(WT)	[R352A] (nM)	R352A
9450	352.5	0.70711	13100	388.5
4725	296.5	3.5355	6550	363
2362.5	256	5.6569	3275	321.5
1181.2	213.5	7.7782	1637.5	272
590.62	147	11.314	818.75	194
295.31	103.5	6.364	409.38	125.5
147.66	89	0	204.69	95
73.828	84	1.4142	102.34	88
36.914	81	1.4142	51.172	86
18.457	82	1.4142	25.586	86.5
9.2285	82.5	0.70711	12.793	86.5
4.6143	81.5	2.1213	6.3965	85
2.3071	81	1.4142	3.1982	84
1.1536	81.5	0.70711	1.5991	84
0.57678	81.5	0.70711	0.79956	86
0.28839	81.5	0.70711	0.39978	87
0.1442	80.5	0.70711	0.19989	85.5
0.072098	82.5	2.1213	0.099945	84
0.036049	81	0	0.049973	85.5
0.018024	82	0	0.024986	85.5

STDEV(R352A	[K523A] (nM)	K523A	STDEV(K523A	[H376A](nM)	H376A (FPS0C	STDEV(H376A
2.1213	7250	383.5	0.70711	8100	187.5	33.23
0	3625	362	15.556	4050	131	2.83
3.5355	1812.5	309.5	3.5355	2025	124.5	9.19
7.0711	906.25	243	9.8995	1012.5	112.5	7.78
11.314	453.12	160	12.728	506.25	104	4.24
10.607	226.56	105.5	3.5355	253.13	98.5	0.71
2.8284	113.28	89	1.4142	126.56	97.5	0.71
1.4142	56.641	86	0	63.28	96.5	2.12
1.4142	28.32	84	0	31.64	93.5	0.71
2.1213	14.16	84.5	2.1213	15.82	95.5	0.71
2.1213	7.0801	83	1.4142	7.91	95.5	2.12
0	3.54	84	1.4142	3.96	94.5	0.71
1.4142	1.77	84	1.4142	1.98	94.5	2.12
2.8284	0.88501	86	1.4142	0.99	93.5	2.12
1.4142	0.4425	82.5	0.70711	0.49	95	0
1.4142	0.22125	85.5	0.70711	0.25	95	0
2.1213	0.11063	83.5	0.70711	0.12	94.5	0.71
0	0.055313	84	1.4142	0.06	93.5	0.71
0.70711	0.027657	82.5	0.70711	0.03	94	0
0.70711	0.013828	85	0	0.02	94.5	2.12

-FPS006PK1)

Figure 6b dNTPase rate of mutants

WT	0.3756	1.0796	1.1784	1.2003	1.2437
STDEV	0.0055	0.0521	0.0876	0.0284	0.003
R352A	0.0143	0.2961	0.3938	0.3858	0.394
STDEV	0.0059	0.0315	0.0195	0.0285	0.0289
H376A	0.0092	0.2156	0.4598	0.563	0.5529
STDEV	0.0083	0.0108	0.0174	0.0207	0.0102
K523A	0.0557	0.1295	0.3382	0.3635	0.3745
STDEV	0.0734	0.0006	0.0169	0.0147	0.01
2Y	0	0	0	0	0
STDEV	0	0	0	0	0
[GTP] (uM)	1	10	50	250	1000

Figure 6E	bar plot of mu

tants

```
dATP_data = [4.6 4.17; 0 0; 4.56 3.42; 0.75 0.71; 0.27 0.27; 0.44 0.7];
dGTP_data = [1.89 1.92; 0.21 0; 3.44 2.31; 0.77 0.57; 0.3 0.26; 0.68 0.67];
dATP_mean = mean(dATP_data,2);
dATP_error = std(dATP_data,0,2);
dGTP_mean = mean(dGTP_data,2);
dGTP_error = std(dGTP_data,0,2);
```

Figure 6F HIV-1 restriction activity

	PLVX CTRL		SAMHD1 WT	
500	25.6	25.5	2.92	
250	16.9	17.2	1.95	1.75
125	9.39	10.4	1.31	1.01
62.5	5.21	5.77		
32.25	3.61	3.49		
0	0	0	0	0

	PLVX CTRL		SAMHD1 WT	
500	28.9	32	5.38	4.28
250	18.9	19.2	3.36	2.73
125	12.1	10.2	2.61	2.21
62.5	5.67	5.72	1.84	1.8
32.25	3.21	3.74		
0	0	0	0	0

R352A		T592V		T592D	
22.3	22.8	1.35	1.02	27.3	26.1
17.9	18.7			17.2	16.7
9.85	7	0.97		11.1	11
6.2				5.33	4.47
4.22	5.19			2.69	3.34
0	0	0		0	0

H376A		K523A	
27.3	28.5	26	24.3
20.1	19.9	13.9	13.8
11.6	10.2	7.93	9.2
6.26	5.85	4.38	4.06
3.14	3.23	2.63	2.21
0	0	0	0

Figure S1A dNTPase HD vs Full-length

HD domain	374.5	359	349.5	331.5	292.5
	2.12132034	1.41421356	2.12132034	7.77817459	0.70710678
[HD] (nM)	7335	3667.5	1833.75	916.875	458.4375
Full-length	381.5	366	359.5	338.5	302
	0.70710678	8.48528137	0.70710678	3.53553391	4.24264069
[FL] (nM)	12000	6000	3000	1500	750

246.5	194.5	159.5	125	108	94.5	89
12.0208153	12.0208153	6.36396103	9.89949494	7.07106781	4.94974747	2.82842713
229.21875	114.609375	57.3046875	28.6523438	14.3261719	7.16308594	3.58154297
260	204.5	158.5	124.5	103	93.5	86.5
7.07106781	4.94974747	6.36396103	4.94974747	0	2.12132034	2.12132034
375	187.5	93.75	46.875	23.4375	11.71875	5.859375

85	83.5	82	81.5	83	82.5	81
4.24264069	2.12132034	2.82842713	2.12132034	1.41421356	0.70710678	1.41421356
1.79077148	0.89538574	0.44769287	0.22384644	0.11192322	0.05596161	0.0279808
83	80.5	80.5	79	79	79.5	78
1.41421356	0.70710678	2.12132034	0	1.41421356	2.12132034	0
2.9296875	1.46484375	0.73242188	0.36621094	0.18310547	0.09155273	0.04577637

82.5
0.70710678
0.0139904
79
1.41421356
0.02288818

Figure S1B dNTPase rates of HD vs FL

FL-SAMHD1	0.09705	0.13758	0.18235	0.22678	0.27111
	0.00477	0.00633	0.01275	0.01590	0.01814
HD domain	0.14206	0.20821	0.27826	0.34691	0.41008
	0.00250	0.00587	0.00667	0.00922	0.01118
Time	180	240	300	360	420

0.31535
0.02301
0.47594
0.01449
480

Figure S1C DNase activity

WT	D311A	2Y	Dnase
1823	1918	1856	6521
1852	1961	1898	7650
1880	1985	1925	8611
1896	2005	1948	9459
1913	2033	1978	10215
1925	2046	1999	10903
1942	2063	2021	11505
1956	2076	2045	12069
1967	2091	2070	12584
1978	2104	2085	13046
1998	2120	2111	13472
2004	2134	2137	13848
2022	2147	2157	14217
2035	2162	2181	14545
2041	2173	2198	14848
2061	2187	2221	15142
2069	2195	2241	15408
2081	2212	2266	15649
2096	2219	2283	15877
2109	2234	2306	16081
2122	2248	2327	16292
2130	2262	2350	16482
2146	2272	2367	16660
2153	2286	2396	16821
2156	2297	2416	16974
2178	2306	2439	17136
2185	2317	2461	17261
2201	2328	2480	17402
2208	2336	2505	17513
2226	2350	2523	17626
2235	2361	2543	17738
2255	2374	2569	17861
2263	2385	2589	17953
2274	2399	2610	18056
2286	2401	2633	18143
2298	2415	2659	18230
2311	2431	2675	18293
2323	2435	2701	18355
2335	2449	2724	18448
2351	2465	2747	18503
2372	2476	2770	18575
2372	2485	2795	18666
2384	2501	2813	18703
2395	2512	2831	18769
2412	2523	2851	18816
2422	2534	2878	18874

2434	2541	2898	18927
2445	2549	2923	18969
2462	2559	2944	19025
2472	2576	2965	19048
2491	2586	2988	19092
2502	2596	3012	19133
2504	2608	3041	19171
2520	2619	3058	19217
2532	2628	3076	19243
2545	2645	3100	19273
2559	2649	3124	19290
2567	2660	3144	19325
2586	2668	3165	19377
2593	2676	3190	19390
2606	2692	3219	19424
2619	2703	3234	19441
2635	2716	3251	19454
2642	2718	3279	19477
2659	2731	3302	19506
2667	2747	3327	19525
2684	2756	3346	19540
2696	2763	3372	19562
2705	2770	3387	19586
2717	2787	3412	19584
2731	2797	3431	19617
2740	2802	3455	19630
2749	2814	3474	19654
2762	2824	3497	19646
2778	2836	3517	19664
2790	2852	3536	19673
2800	2854	3562	19710
2810	2869	3583	19714
2824	2877	3605	19713
2831	2889	3626	19722
2843	2896	3647	19738
2860	2909	3668	19738
2874	2918	3696	19756
2884	2927	3719	19767
2899	2943	3739	19759
2907	2947	3763	19792
2919	2963	3781	19784
2932	2969	3806	19803
2948	2984	3821	19797
2951	2990	3847	19791
2964	2994	3868	19808
2976	3007	3885	19801
2989	3022	3913	19827
3001	3031	3933	19819

3014	3038	3951	19836
3020	3044	3978	19812
3040	3061	4003	19840
3047	3073	4026	19832
3054	3083	4049	19849
3066	3084	4065	19873
3078	3099	4080	19839
3092	3108	4105	19867
3104	3118	4128	19845
3112	3127	4154	19877
3121	3140	4173	19868
3141	3151	4193	19861
3151	3162	4217	19871
3159	3167	4238	19881
3168	3182	4254	19882
3187	3187	4275	19883
3197	3194	4295	19885
3215	3211	4315	19865
3220	3219	4348	19864
3230	3226	4361	19863
3244	3238	4381	19891
3253	3248	4405	19880
3265	3254	4434	19878
3279	3267	4448	19895
3285	3281	4467	19867
3293	3288	4490	19862
3312	3297	4519	19885
3326	3304	4536	19877
3333	3313	4552	19889
3343	3324	4580	19892
3352	3338	4594	19872
3363	3348	4615	19868
3373	3358	4643	19872
3386	3369	4661	19884
3408	3378	4680	19879
3407	3388	4708	19858
3422	3398	4727	19877
3437	3398	4739	19874
3438	3417	4767	19851
3459	3422	4780	19871
3468	3433	4802	19874
3480	3450	4829	19860
3488	3452	4846	19854
3499	3463	4864	19865
3508	3471	4890	19856
3522	3490	4910	19863
3531	3490	4926	19852
3546	3507	4949	19859

3553	3517	4970	19842
3565	3523	4991	19856
3581	3532	5009	19849
3591	3538	5029	19842
3595	3553	5046	19853
3605	3551	5079	19827
3617	3577	5085	19827
3626	3583	5112	19848
3640	3588	5133	19828
3652	3598	5149	19843
3667	3609	5169	19827
3671	3612	5195	19831
3685	3632	5214	19818
3701	3637	5236	19840
3709	3653	5255	19820
3715	3658	5274	19828
3735	3667	5288	19819
3746	3682	5321	19823
3753	3689	5333	19814
3762	3698	5359	19814
3763	3706	5366	19806
3789	3714	5387	19800
3796	3725	5402	19813
3806	3733	5434	19797
3816	3745	5449	19792
3829	3751	5483	19819
3844	3767	5489	19806
3847	3774	5511	19790
3861	3791	5525	19779
3861	3793	5551	19784
3886	3803	5569	19773
3893	3812	5583	19774
3904	3817	5602	19772
3908	3829	5621	19762
3923	3839	5644	19770
3930	3852	5667	19769
3946	3860	5682	19764
3957	3866	5705	19752
3962	3880	5723	19757
3979	3887	5739	19760
3988	3894	5769	19728
4004	3896	5779	19755
4010	3912	5803	19746
4017	3924	5821	19737
4026	3941	5837	19729
4038	3945	5859	19745
4043	3955	5873	19718
4062	3961	5894	19729

4071	3970	5914	19710
4083	3978	5935	19731
4090	3990	5958	19712
4107	4004	5968	19718
4117	4011	5977	19715
4126	4022	6003	19694
4140	4026	6022	19703
4149	4044	6041	19684
4156	4047	6063	19699
4170	4057	6083	19679
4179	4058	6105	19695
4192	4075	6121	19689
4198	4086	6130	19674
4212	4091	6152	19692
4221	4103	6166	19675
4229	4109	6190	19680
4235	4118	6199	19679
4255	4130	6232	19653
4261	4134	6236	19664
4275	4146	6263	19658
4285	4154	6289	19663
4291	4171	6304	19656
4308	4174	6322	19648
4316	4178	6332	19647
4326	4203	6354	19642
4340	4199	6374	19631
4349	4215	6381	19635
4358	4221	6411	19640
4370	4231	6423	19642
4382	4242	6441	19614
4390	4246	6460	19616
4402	4258	6476	19630
4411	4271	6491	19613
4409	4272	6517	19614
4435	4286	6536	19618
4446	4301	6540	19604
4445	4302	6562	19599
4460	4315	6569	19588
4467	4324	6596	19594
4478	4334	6615	19604
4496	4344	6632	19609
4501	4351	6651	19580
4514	4363	6669	19581
4522	4373	6683	19580
4532	4378	6703	19571
4541	4382	6714	19565
4553	4390	6733	19568
4558	4405	6756	19570

4574	4417	6778	19571
4578	4415	6784	19555
4595	4431	6803	19561
4602	4442	6828	19556
4612	4441	6842	19557
4618	4455	6856	19549
4636	4462	6867	19548
4644	4471	6888	19543
4653	4486	6906	19552
4657	4488	6917	19541
4670	4504	6935	19534
4674	4517	6948	19523
4691	4519	6970	19513
4699	4526	6980	19501
4704	4537	6999	19500
4722	4542	7023	19513
4735	4554	7033	19514
4740	4560	7050	19514
4754	4575	7068	19486
4762	4582	7078	19497
4766	4586	7103	19497
4784	4603	7112	19472
4781	4605	7139	19486
4806	4613	7150	19485
4816	4624	7163	19485
4822	4628	7177	19481
4829	4637	7199	19489
4841	4654	7217	19476
4851	4653	7231	19465
4857	4665	7244	19453
4867	4677	7269	19455
4881	4690	7274	19460
4889	4677	7298	19455
4900	4693	7316	19448
4905	4714	7326	19442
4918	4720	7343	19460
4930	4728	7353	19442
4943	4740	7372	19443
4948	4743	7384	19432
4952	4752	7399	19424
4964	4757	7425	19427
4978	4770	7442	19434
4984	4776	7455	19414
4996	4784	7462	19421
5010	4792	7482	19394
5016	4801	7505	19412
5022	4809	7508	19407
5033	4818	7533	19408

5041	4826	7550	19423
5058	4831	7558	19390
5066	4848	7571	19373
5080	4851	7579	19382
5079	4860	7605	19392
5085	4867	7618	19394
5100	4878	7632	19381
5112	4883	7648	19400
5122	4899	7657	19363
5124	4907	7668	19367
5143	4916	7690	19367
5150	4913	7708	19360
5156	4925	7716	19368
5169	4939	7739	19353
5175	4947	7752	19332
5187	4958	7762	19334
5196	4956	7779	19347
5202	4975	7788	19352
5210	4977	7811	19331
5222	4985	7818	19332
5229	4994	7839	19346
5240	4996	7845	19333
5257	5012	7867	19312
5259	5018	7876	19318
5268	5031	7900	19320
5278	5035	7909	19317
5285	5045	7921	19321
5291	5054	7943	19308
5303	5057	7951	19312
5322	5065	7965	19304
5323	5080	7983	19307
5336	5085	8006	19291
5340	5094	8013	19303
5348	5099	8027	19308
5362	5100	8039	19294
5371	5119	8052	19282
5373	5125	8064	19289
5384	5133	8068	19277
5396	5138	8096	19271
5404	5142	8114	19272
5420	5159	8114	19252
5418	5157	8147	19250
5437	5177	8149	19250
5447	5182	8164	19247
5456	5178	8177	19249
5460	5191	8188	19239
5466	5198	8205	19239
5474	5203	8211	19236

5483	5218	8242	19243
5495	5227	8244	19224
5502	5234	8263	19225
5515	5244	8269	19237
5514	5253	8289	19221
5531	5251	8299	19230
5537	5271	8312	19206
5552	5269	8320	19208
5563	5277	8337	19219
5568	5290	8345	19208
5574	5300	8361	19201
5589	5304	8373	19193
5591	5317	8388	19205
5596	5319	8401	19202
5611	5328	8419	19191
5616	5334	8425	19183
5624	5339	8443	19182
5640	5347	8455	19168
5641	5364	8465	19176
5658	5373	8482	19168
5662	5380	8503	19166
5667	5376	8508	19148
5678	5390	8527	19164
5685	5396	8538	19165
5698	5404	8553	19163
5709	5415	8568	19144
5718	5424	8573	19158
5723	5426	8586	19132
5728	5436	8600	19138
5738	5442	8610	19135
5748	5452	8625	19131
5752	5457	8638	19127
5766	5466	8653	19105
5782	5475	8670	19142
5793	5478	8670	19122
5787	5480	8682	19109
5799	5497	8706	19107
5809	5501	8714	19097
5813	5517	8726	19099
5823	5512	8735	19091
5824	5528	8753	19109
5852	5539	8767	19101
5850	5539	8763	19088
5861	5538	8791	19088
5870	5556	8799	19087
5867	5567	8813	19078
5880	5568	8820	19091
5894	5574	8846	19079

5903	5584	8848	19053
5911	5598	8868	19083
5917	5605	8884	19077
5924	5605	8883	19061
5937	5615	8904	19063
5948	5618	8912	19054
5954	5629	8921	19057
5954	5636	8932	19046
5960	5645	8946	19042
5968	5649	8956	19056
5981	5653	8964	19050
5983	5674	8989	19031
5993	5665	9000	19030
6004	5678	9008	19030
6006	5689	9016	19026
6025	5692	9036	19006
6030	5703	9040	19025
6036	5709	9066	19037
6042	5714	9061	19020
6057	5722	9078	19015
6059	5732	9076	19003
6069	5729	9095	19012
6072	5752	9116	19006
6089	5751	9121	18991
6100	5759	9131	18982
6101	5766	9142	19003
6115	5776	9162	18987
6111	5788	9168	18991
6129	5788	9178	18979
6137	5793	9191	18976
6135	5802	9206	18966
6146	5809	9220	18957
6162	5822	9225	18955
6158	5826	9243	18951
6171	5831	9250	18955
6176	5840	9261	18954
6185	5842	9274	18939
6204	5851	9286	18945
6208	5865	9288	18938
6208	5868	9303	18925
6223	5875	9313	18947
6235	5878	9323	18920
6230	5897	9341	18922
6249	5903	9356	18942
6251	5902	9357	18927
6244	5908	9377	18908
6261	5915	9389	18916
6270	5918	9403	18920

6279	5934	9408	18913
6293	5936	9413	18918
6291	5949	9424	18910
6296	5960	9445	18892
6313	5971	9447	18884
6321	5964	9449	18889
6321	5973	9472	18892
6337	5989	9479	18881
6341	5994	9493	18886
6345	6000	9511	18896
6357	6001	9521	18872
6360	6012	9536	18881
6371	6023	9540	18866
6379	6025	9553	18859
6386	6031	9568	18861
6405	6035	9570	18877
6407	6051	9577	18857
6406	6046	9596	18861
6418	6063	9608	18854
6422	6065	9614	18837
6422	6079	9634	18841
6441	6084	9642	18843
6454	6095	9653	18838
6459	6107	9660	18845
6466	6113	9671	18812
6474	6109	9685	18829
6479	6120	9688	18817
6480	6132	9711	18826
6495	6131	9710	18801
6499	6141	9725	18815
6505	6147	9733	18808
6516	6151	9741	18801
6520	6160	9757	18816
6533	6159	9765	18792
6537	6168	9787	18791
6546	6182	9784	18811
6543	6182	9788	18788
6567	6192	9826	18791
6572	6198	9822	18780
6573	6209	9830	18765
6577	6218	9835	18782
6593	6225	9855	18764
6592	6225	9866	18768
6605	6232	9875	18773
6611	6241	9889	18775
6630	6252	9889	18773
6625	6266	9902	18747
6636	6263	9926	18756

6640	6271	9928	18745
6645	6278	9940	18757
6656	6291	9946	18748
6656	6299	9956	18736
6665	6295	9970	18737
6667	6303	9974	18719
6683	6317	9988	18739
6689	6327	9992	18730
6692	6333	10012	18735
6699	6343	10021	18699
6712	6345	10022	18722
6717	6346	10034	18716
6721	6352	10060	18706
6732	6365	10070	18710
6736	6360	10083	18696
6743	6379	10081	18713
6747	6379	10089	18695
6761	6382	10100	18690
6768	6395	10110	18691
6771	6398	10125	18679
6778	6411	10123	18688
6797	6418	10139	18692
6800	6425	10148	18681
6790	6431	10166	18681
6807	6442	10182	18674
6811	6448	10187	18668
6825	6457	10193	18649
6822	6457	10206	18659
6839	6476	10216	18661
6849	6468	10218	18647
6849	6479	10234	18660
6857	6482	10242	18638
6865	6486	10254	18649
6869	6500	10260	18648
6881	6507	10275	18642
6880	6499	10269	18646
6893	6520	10280	18632
6905	6529	10295	18643
6900	6531	10314	18604
6912	6540	10324	18632
6924	6539	10338	18615
6928	6562	10350	18614
6925	6563	10340	18592
6935	6570	10352	18610
6942	6566	10373	18611
6958	6584	10372	18616
6958	6588	10401	18588
6963	6591	10396	18597

6967	6602	10416	18580
6978	6605	10429	18584
6988	6607	10434	18595
6992	6625	10443	18584
7000	6636	10445	18569
7008	6629	10464	18574
7015	6637	10470	18569
7019	6645	10483	18577
7031	6651	10505	18563
7033	6654	10500	18584
7048	6673	10509	18548
7056	6671	10521	18549
7050	6674	10545	18562
7058	6684	10538	18543
7070	6687	10552	18543
7077	6698	10552	18545
7076	6698	10576	18549
7089	6707	10590	18523
7091	6716	10580	18537
7097	6726	10601	18527
7106	6738	10604	18532
7111	6734	10622	18534
7124	6751	10619	18527
7127	6747	10641	18514
7130	6761	10654	18519
7142	6759	10653	18524
7146	6772	10659	18510
7159	6778	10670	18509
7160	6782	10681	18507
7165	6788	10683	18510
7169	6789	10695	18503
7174	6803	10708	18493
7186	6809	10716	18492
7186	6808	10719	18481
7194	6819	10727	18488
7197	6828	10744	18486
7205	6828	10756	18487
7219	6838	10761	18473
7221	6847	10767	18468
7223	6860	10787	18472
7235	6856	10786	18472
7242	6871	10801	18471
7243	6870	10801	18460
7246	6863	10822	18444
7255	6882	10832	18455
7257	6898	10839	18455
7271	6895	10846	18445
7288	6899	10836	18445

7293	6908	10860	18432
7289	6924	10867	18427
7288	6924	10883	18445
7303	6934	10875	18442
7308	6924	10906	18423
7311	6943	10909	18413
7323	6944	10928	18431
7324	6958	10930	18424
7337	6968	10929	18411
7344	6970	10934	18407
7341	6964	10940	18398
7354	6972	10964	18416
7357	6989	10961	18408
7369	6988	10980	18402
7375	7001	10984	18385
7380	7003	10991	18390
7381	7010	11001	18396
7382	7010	11004	18391
7394	7019	11012	18378
7399	7021	11019	18384
7408	7027	11044	18396
7415	7037	11043	18368
7416	7044	11060	18389
7415	7048	11050	18370
7432	7055	11059	18380
7451	7056	11060	18386
7441	7069	11076	18361
7451	7080	11109	18373
7447	7084	11109	18351
7469	7094	11103	18366
7464	7087	11128	18362
7474	7106	11124	18338
7480	7110	11134	18351
7472	7110	11135	18335
7494	7122	11139	18333
7500	7125	11151	18347
7500	7123	11171	18348
7514	7138	11176	18342
7515	7138	11181	18330
7514	7153	11194	18338
7534	7154	11200	18328
7530	7158	11218	18303
7542	7170	11209	18301
7539	7167	11229	18322
7542	7178	11232	18309
7562	7183	11234	18319
7564	7193	11258	18307
7557	7201	11261	18314

7575	7196	11271	18301
7567	7216	11277	18292
7585	7210	11276	18304
7589	7220	11286	18289
7589	7219	11296	18278
7597	7232	11305	18285
7599	7244	11310	18295
7620	7232	11331	18278
7616	7252	11325	18271
7616	7253	11333	18273
7624	7262	11334	18286
7634	7259	11358	18267
7645	7269	11352	18277
7643	7278	11372	18280
7650	7294	11376	18259
7654	7291	11380	18266
7660	7291	11382	18247
7669	7296	11401	18259
7676	7307	11405	18259
7682	7307	11417	18259
7687	7324	11426	18252
7698	7316	11430	18241
7693	7334	11443	18236
7696	7336	11438	18228
7707	7333	11443	18233
7706	7348	11458	18235
7724	7351	11467	18232
7726	7352	11479	18230
7727	7364	11474	18222
7735	7379	11478	18225
7741	7374	11490	18241
7745	7372	11506	18207
7755	7382	11509	18219
7760	7386	11513	18207
7763	7400	11525	18198
7773	7401	11527	18214
7770	7414	11549	18202
7780	7410	11549	18197
7783	7413	11564	18210
7796	7424	11566	18196
7793	7439	11583	18195
7804	7443	11580	18195
7803	7449	11587	18188
7821	7450	11588	18175
7814	7453	11597	18189
7830	7458	11612	18165
7827	7457	11624	18178
7839	7472	11623	18169

7837	7484	11640	18168
7852	7482	11667	18156
7860	7479	11649	18180
7859	7491	11655	18153
7855	7496	11662	18154
7876	7504	11683	18175
7869	7513	11678	18163
7882	7518	11696	18152
7883	7520	11705	18167
7890	7528	11706	18157
7897	7538	11712	18159
7896	7540	11716	18133
7914	7542	11728	18137
7908	7551	11728	18129
7911	7548	11743	18128
7923	7554	11757	18152
7922	7571	11758	18141
7933	7569	11759	18143
7934	7571	11769	18133
7943	7589	11778	18132
7945	7585	11783	18128
7949	7588	11779	18121
7963	7588	11806	18110
7974	7601	11810	18117
7973	7605	11815	18115
7976	7603	11821	18130
7981	7620	11840	18109
7991	7626	11835	18086
7997	7630	11855	18117
8001	7632	11830	18105
8004	7638	11848	18105
8007	7646	11852	18099
8004	7642	11869	18105
8018	7658	11877	18088
8024	7660	11891	18102
8025	7663	11897	18088
8037	7662	11902	18082
8036	7675	11909	18090
8044	7681	11925	18093
8045	7681	11916	18094
8051	7690	11922	18083
8065	7690	11940	18099
8067	7700	11942	18083
8065	7707	11950	18075
8072	7718	11956	18070
8077	7718	11949	18061
8079	7717	11966	18075
8084	7720	11966	18052

8097	7735	11971	18064
8103	7738	11976	18062
8105	7746	11998	18059

Rnase	Buffer
1694	1676
1711	1695
1723	1703
1735	1710
1739	1718
1748	1722
1758	1727
1761	1725
1765	1736
1766	1741
1777	1743
1782	1744
1787	1749
1787	1754
1791	1761
1800	1764
1807	1764
1812	1768
1818	1772
1820	1777
1828	1777
1834	1786
1839	1784
1844	1788
1849	1789
1852	1797
1857	1803
1862	1801
1867	1807
1877	1810
1877	1816
1879	1814
1883	1819
1891	1826
1896	1824
1897	1830
1904	1835
1907	1837
1916	1842
1918	1840
1923	1851
1926	1847
1934	1856
1935	1855
1943	1860
1949	1863

1949	1872
1956	1873
1963	1875
1972	1877
1970	1884
1976	1889
1979	1894
1985	1898
1989	1896
1997	1904
2003	1903
2006	1911
2011	1912
2011	1919
2016	1923
2025	1926
2027	1932
2031	1932
2039	1934
2047	1938
2045	1943
2053	1944
2058	1947
2064	1957
2065	1951
2072	1955
2081	1957
2081	1960
2089	1968
2091	1973
2092	1974
2097	1976
2099	1986
2107	1986
2120	1989
2116	1995
2126	2001
2128	1999
2135	2002
2140	2008
2136	2015
2153	2015
2155	2018
2156	2022
2162	2026
2170	2035
2175	2032
2176	2034

2183	2041
2187	2038
2194	2045
2194	2049
2198	2052
2204	2060
2207	2059
2211	2064
2220	2069
2226	2076
2224	2077
2232	2075
2240	2078
2240	2086
2242	2091
2253	2091
2256	2101
2259	2095
2261	2104
2265	2108
2275	2109
2280	2116
2285	2117
2293	2118
2299	2123
2298	2128
2308	2131
2307	2134
2311	2136
2322	2141
2323	2144
2326	2144
2332	2152
2336	2157
2347	2155
2345	2160
2348	2168
2351	2168
2358	2172
2363	2177
2369	2182
2374	2184
2377	2186
2380	2193
2387	2197
2389	2199
2400	2200
2405	2204

2407	2208
2411	2212
2415	2213
2422	2225
2427	2223
2429	2228
2437	2229
2442	2236
2441	2243
2451	2244
2453	2244
2458	2247
2461	2250
2465	2258
2472	2262
2476	2266
2482	2272
2486	2273
2492	2273
2497	2276
2501	2282
2505	2284
2514	2287
2508	2290
2520	2296
2521	2297
2526	2303
2535	2304
2539	2309
2541	2310
2547	2316
2551	2321
2562	2326
2563	2324
2564	2333
2572	2333
2577	2337
2581	2345
2584	2345
2595	2345
2596	2347
2598	2354
2604	2358
2610	2362
2614	2366
2614	2371
2624	2377
2630	2376

2633	2379
2638	2385
2643	2388
2649	2394
2652	2389
2660	2396
2663	2404
2667	2404
2669	2408
2675	2416
2684	2415
2683	2420
2687	2422
2693	2431
2701	2428
2702	2437
2704	2434
2716	2445
2722	2446
2727	2448
2730	2454
2733	2454
2736	2456
2745	2464
2744	2465
2754	2469
2753	2476
2755	2474
2759	2484
2768	2484
2776	2486
2783	2491
2783	2494
2790	2499
2793	2504
2801	2506
2802	2510
2812	2513
2814	2512
2816	2521
2829	2520
2827	2529
2831	2527
2835	2537
2841	2536
2850	2542
2854	2539
2852	2551

2859	2553
2863	2556
2871	2560
2873	2560
2880	2570
2885	2563
2895	2579
2888	2576
2897	2584
2903	2583
2910	2589
2915	2588
2917	2593
2920	2599
2927	2600
2930	2603
2937	2610
2945	2611
2943	2615
2956	2619
2950	2623
2960	2626
2961	2631
2968	2637
2977	2637
2981	2640
2986	2644
2987	2647
2996	2650
2994	2654
3002	2656
3002	2659
3011	2669
3012	2664
3021	2668
3025	2677
3031	2677
3040	2682
3040	2686
3050	2688
3051	2695
3055	2699
3058	2703
3065	2708
3069	2709
3077	2710
3080	2717
3083	2721

3088	2723
3092	2730
3096	2727
3102	2730
3106	2737
3109	2740
3117	2742
3123	2753
3128	2752
3131	2754
3131	2762
3136	2757
3149	2765
3155	2768
3160	2778
3158	2783
3159	2776
3172	2781
3167	2783
3171	2785
3179	2792
3187	2797
3191	2801
3198	2807
3196	2807
3203	2810
3207	2817
3219	2818
3221	2821
3230	2824
3226	2825
3235	2829
3236	2836
3244	2842
3250	2840
3252	2844
3257	2850
3263	2858
3267	2856
3266	2865
3274	2867
3280	2869
3287	2868
3290	2873
3292	2879
3300	2877
3300	2886
3311	2887

3318	2888
3317	2893
3323	2895
3328	2905
3329	2903
3338	2912
3338	2914
3345	2915
3350	2921
3355	2921
3363	2925
3360	2927
3365	2928
3369	2936
3375	2938
3385	2947
3393	2942
3389	2953
3395	2952
3400	2951
3404	2965
3406	2964
3413	2966
3422	2969
3431	2974
3422	2983
3436	2980
3432	2985
3439	2985
3448	2984
3447	3000
3456	2999
3459	3000
3461	3007
3466	3013
3477	3011
3478	3021
3479	3020
3493	3025
3494	3025
3497	3031
3501	3033
3507	3040
3509	3041
3513	3047
3520	3048
3528	3053
3532	3054

3534	3053
3539	3060
3542	3063
3545	3063
3547	3068
3557	3071
3555	3081
3565	3080
3571	3084
3579	3091
3576	3092
3585	3095
3581	3099
3591	3096
3598	3108
3599	3110
3605	3112
3608	3109
3611	3122
3617	3126
3621	3128
3622	3134
3635	3129
3636	3141
3642	3133
3648	3141
3651	3147
3655	3148
3654	3152
3658	3155
3668	3159
3668	3161
3680	3169
3687	3166
3689	3178
3693	3183
3694	3180
3696	3187
3699	3189
3708	3192
3718	3196
3714	3197
3724	3197
3722	3208
3725	3208
3735	3212
3738	3214
3736	3216

3742	3223
3750	3224
3755	3227
3759	3234
3766	3236
3763	3236
3771	3245
3776	3244
3782	3255
3788	3255
3788	3260
3795	3262
3800	3264
3803	3273
3810	3270
3809	3278
3813	3282
3819	3283
3824	3286
3829	3289
3832	3294
3837	3295
3845	3297
3853	3308
3846	3304
3851	3311
3855	3307
3865	3314
3861	3323
3870	3318
3875	3325
3878	3327
3888	3337
3891	3333
3892	3342
3896	3351
3903	3346
3908	3345
3908	3352
3913	3354
3919	3362
3928	3369
3929	3370
3926	3369
3933	3371
3945	3377
3950	3382
3950	3378

3955	3391
3959	3389
3967	3394
3971	3400
3974	3405
3977	3394
3978	3410
3984	3410
3988	3415
3989	3420
3994	3424
4001	3422
4007	3427
4010	3437
4012	3431
4018	3433
4024	3436
4020	3443
4029	3441
4036	3453
4035	3450
4046	3455
4045	3464
4056	3462
4061	3471
4065	3469
4067	3473
4067	3479
4076	3483
4083	3481
4082	3488
4083	3484
4086	3495
4097	3503
4098	3502
4100	3509
4106	3509
4112	3505
4107	3515
4121	3516
4118	3520
4129	3524
4133	3532
4142	3534
4133	3537
4139	3540
4150	3542
4153	3549

4151	3546
4169	3553
4162	3555
4165	3563
4173	3559
4174	3564
4184	3571
4192	3569
4187	3573
4191	3578
4199	3580
4209	3583
4205	3589
4208	3595
4217	3590
4224	3591
4224	3597
4230	3604
4224	3609
4234	3618
4246	3615
4247	3620
4250	3627
4252	3625
4256	3624
4265	3639
4271	3636
4265	3645
4275	3644
4275	3648
4289	3648
4286	3648
4293	3659
4300	3661
4298	3668
4306	3667
4309	3675
4317	3673
4322	3678
4322	3676
4324	3686
4334	3685
4339	3687
4340	3691
4344	3693
4348	3707
4343	3702
4351	3707

4361	3711
4363	3712
4362	3710
4368	3724
4376	3718
4376	3727
4382	3732
4382	3739
4391	3731
4397	3744
4395	3739
4405	3742
4404	3744
4402	3754
4412	3757
4418	3760
4422	3765
4429	3763
4427	3769
4425	3775
4442	3780
4445	3782
4449	3782
4452	3784
4454	3789
4464	3794
4466	3794
4464	3798
4477	3799
4480	3797
4480	3808
4488	3812
4493	3816
4497	3816
4503	3822
4507	3819
4507	3826
4510	3828
4510	3829
4520	3842
4526	3840
4533	3843
4531	3844
4533	3847
4543	3853
4551	3849
4547	3859
4553	3854

4551	3865
4562	3869
4567	3870
4565	3875
4569	3879
4581	3878
4582	3881
4588	3883
4592	3895
4598	3889
4599	3899
4602	3899
4606	3902
4610	3906
4622	3915
4615	3913
4623	3911
4632	3917
4628	3921
4633	3923
4639	3929
4643	3929
4648	3935
4652	3934
4660	3940
4663	3946
4666	3940
4663	3953
4666	3958
4673	3950
4685	3959
4691	3964
4693	3968
4697	3968
4691	3963
4706	3966
4709	3973
4710	3977
4720	3987
4724	3991
4720	3989
4726	3994
4729	3997
4732	4000
4737	4003
4744	4005
4748	4012
4747	4014

4758	4013
4759	4022
4761	4026
4772	4028
4770	4022
4772	4037
4778	4029
4786	4034
4792	4039
4796	4036
4810	4045
4798	4052
4801	4057
4811	4058
4810	4059
4823	4061
4830	4060
4830	4060
4833	4074
4835	4069
4847	4070
4843	4082
4853	4082
4854	4092
4863	4087
4860	4091
4868	4087
4875	4101
4871	4099
4875	4106
4877	4107
4881	4110
4887	4108
4895	4115
4891	4120
4896	4127
4909	4125
4909	4131
4908	4137
4915	4135
4921	4138
4934	4142
4926	4139
4934	4140
4944	4144
4942	4154
4939	4158
4950	4153

4957	4163
4958	4158
4963	4166

Figure S1D RNase activity

WT	D311A	2Y
2348	2307	2426
2389	2335	2453
2417	2348	2474
2432	2364	2490
2449	2376	2500
2464	2387	2515
2481	2397	2531
2496	2412	2539
2513	2418	2549
2522	2426	2562
2533	2438	2565
2549	2447	2576
2559	2460	2587
2567	2465	2597
2574	2468	2610
2584	2481	2618
2602	2488	2628
2610	2499	2632
2621	2508	2646
2630	2514	2656
2643	2521	2662
2649	2530	2675
2660	2541	2680
2675	2546	2692
2682	2556	2699
2693	2563	2707
2700	2569	2720
2711	2575	2729
2726	2583	2737
2729	2595	2745
2744	2602	2755
2753	2607	2763
2760	2623	2774
2769	2622	2782
2779	2630	2792
2790	2637	2801
2799	2647	2808
2808	2654	2816
2820	2664	2826
2830	2672	2834
2839	2677	2847
2845	2687	2849
2860	2698	2861
2862	2702	2871
2876	2708	2880

2886	2717	2887
2893	2725	2899
2904	2728	2907
2911	2737	2913
2924	2746	2921
2930	2756	2932
2945	2760	2937
2950	2770	2951
2958	2775	2961
2969	2784	2969
2979	2790	2980
2988	2796	2988
2999	2805	2998
3006	2810	3002
3015	2817	3011
3022	2831	3022
3031	2833	3029
3038	2843	3041
3052	2848	3056
3061	2856	3061
3071	2861	3068
3078	2869	3073
3091	2881	3087
3100	2887	3090
3106	2893	3106
3115	2899	3114
3125	2910	3125
3138	2911	3132
3144	2918	3141
3152	2928	3147
3160	2934	3155
3172	2943	3164
3179	2952	3176
3186	2955	3186
3194	2964	3193
3210	2972	3204
3219	2977	3215
3222	2987	3219
3232	2994	3228
3244	2999	3238
3257	3007	3250
3264	3019	3256
3272	3023	3267
3282	3032	3276
3293	3034	3292
3299	3041	3297
3311	3050	3310
3320	3057	3313

3328	3068	3334
3334	3071	3336
3350	3079	3348
3358	3087	3359
3361	3094	3370
3375	3100	3385
3389	3110	3390
3393	3114	3400
3403	3120	3416
3413	3128	3420
3423	3136	3435
3432	3142	3447
3437	3150	3451
3451	3158	3465
3456	3163	3475
3466	3171	3481
3476	3178	3497
3490	3188	3509
3501	3197	3520
3506	3202	3529
3518	3207	3543
3520	3216	3552
3534	3227	3565
3542	3228	3571
3552	3235	3580
3565	3242	3589
3576	3254	3607
3577	3257	3613
3585	3265	3628
3598	3269	3633
3605	3278	3643
3618	3284	3654
3626	3288	3666
3635	3299	3679
3643	3303	3691
3654	3315	3702
3666	3318	3711
3677	3326	3716
3684	3336	3731
3684	3341	3738
3700	3353	3750
3710	3360	3763
3716	3360	3772
3729	3372	3780
3736	3376	3798
3747	3383	3806
3752	3391	3816
3761	3400	3827

3776	3403	3839
3784	3411	3846
3789	3419	3851
3796	3422	3871
3813	3434	3882
3818	3436	3891
3824	3448	3902
3833	3453	3910
3846	3464	3917
3856	3462	3935
3864	3477	3946
3871	3481	3954
3880	3493	3967
3889	3497	3976
3900	3505	3988
3908	3511	3997
3914	3519	4011
3925	3521	4020
3938	3529	4031
3948	3535	4045
3948	3545	4055
3965	3556	4072
3976	3560	4078
3985	3568	4085
3988	3570	4099
4001	3578	4108
4010	3583	4120
4018	3596	4132
4030	3605	4147
4041	3611	4151
4045	3614	4162
4054	3622	4182
4061	3631	4180
4075	3634	4200
4087	3642	4211
4095	3652	4224
4102	3662	4230
4109	3662	4246
4122	3673	4256
4126	3684	4265
4139	3685	4278
4148	3697	4289
4150	3707	4295
4167	3706	4310
4173	3716	4320
4180	3720	4332
4194	3729	4347
4205	3736	4355

4209	3743	4370
4222	3750	4379
4234	3758	4391
4245	3763	4398
4252	3771	4413
4255	3775	4424
4268	3790	4434
4278	3794	4448
4283	3795	4461
4295	3800	4479
4302	3809	4482
4313	3816	4488
4321	3824	4504
4329	3837	4520
4337	3841	4527
4347	3846	4542
4355	3856	4556
4368	3862	4558
4373	3859	4574
4386	3876	4582
4390	3884	4591
4401	3887	4608
4414	3896	4619
4417	3899	4620
4432	3907	4646
4437	3910	4648
4452	3920	4661
4451	3930	4671
4461	3930	4684
4479	3944	4697
4480	3943	4712
4489	3953	4720
4499	3960	4733
4503	3968	4743
4511	3972	4751
4520	3980	4764
4532	3989	4777
4540	3993	4792
4549	3999	4795
4556	4016	4808
4561	4016	4825
4578	4013	4830
4585	4029	4842
4592	4034	4855
4600	4040	4861
4613	4051	4876
4617	4055	4895
4629	4060	4903

4632	4066	4912
4644	4070	4924
4656	4084	4932
4668	4088	4946
4670	4094	4955
4677	4094	4973
4686	4106	4986
4698	4108	4997
4705	4122	5007
4710	4126	5017
4722	4128	5029
4732	4136	5043
4741	4145	5047
4750	4147	5064
4757	4157	5073
4766	4161	5086
4773	4168	5097
4786	4178	5111
4787	4182	5118
4800	4188	5127
4810	4195	5138
4819	4197	5154
4826	4207	5164
4838	4212	5174
4845	4217	5187
4853	4221	5196
4862	4231	5215
4871	4230	5224
4875	4245	5232
4885	4251	5245
4891	4256	5256
4906	4261	5270
4914	4263	5279
4921	4278	5288
4927	4282	5297
4944	4289	5319
4943	4290	5324
4955	4297	5337
4966	4304	5346
4972	4313	5357
4983	4325	5364
4990	4327	5380
5000	4328	5399
5005	4335	5408
5015	4342	5422
5023	4349	5429
5032	4353	5442
5045	4368	5452

5056	4369	5465
5063	4379	5471
5069	4375	5487
5077	4384	5499
5086	4391	5507
5096	4402	5518
5104	4405	5533
5104	4407	5543
5124	4418	5559
5122	4428	5565
5140	4433	5576
5145	4438	5591
5156	4439	5607
5163	4450	5614
5174	4454	5629
5182	4458	5634
5189	4468	5644
5201	4473	5655
5204	4478	5673
5214	4483	5686
5218	4488	5696
5229	4495	5709
5237	4504	5723
5246	4509	5734
5262	4515	5741
5261	4521	5755
5275	4530	5764
5284	4529	5779
5296	4537	5790
5299	4546	5801
5317	4554	5810
5316	4555	5815
5323	4562	5838
5328	4568	5843
5347	4578	5856
5350	4575	5869
5357	4585	5880
5365	4592	5891
5374	4596	5904
5389	4601	5919
5395	4610	5921
5396	4616	5938
5414	4621	5948
5414	4621	5960
5424	4633	5969
5432	4636	5984
5444	4649	5996
5453	4646	6004

5454	4649	6018
5469	4662	6031
5471	4667	6040
5486	4671	6058
5491	4677	6063
5501	4686	6074
5515	4688	6084
5517	4697	6099
5522	4703	6114
5534	4709	6121
5541	4706	6132
5549	4719	6143
5561	4720	6162
5568	4734	6168
5577	4732	6169
5582	4733	6187
5596	4751	6200
5601	4753	6209
5605	4757	6223
5617	4765	6235
5626	4771	6244
5631	4773	6253
5644	4782	6269
5649	4783	6280
5659	4788	6285
5661	4796	6305
5675	4805	6314
5680	4811	6320
5690	4813	6335
5702	4812	6347
5708	4822	6357
5717	4832	6365
5719	4837	6379
5733	4838	6393
5740	4844	6402
5744	4851	6420
5757	4848	6428
5761	4859	6432
5776	4868	6443
5778	4874	6458
5794	4879	6469
5797	4881	6484
5800	4885	6491
5807	4895	6504
5817	4899	6515
5833	4908	6533
5836	4907	6539
5846	4922	6549

5853	4924	6560
5861	4925	6574
5872	4935	6583
5876	4939	6593
5879	4952	6608
5891	4950	6620
5902	4954	6624
5908	4964	6642
5924	4964	6647
5925	4978	6659
5938	4977	6669
5945	4977	6679
5946	4985	6698
5959	4998	6711
5970	4998	6716
5977	5005	6730
5986	5009	6737
5993	5016	6748
6000	5020	6761
6007	5022	6774
6017	5028	6787
6022	5037	6796
6036	5038	6798
6042	5046	6813
6037	5053	6827
6063	5058	6845
6064	5060	6853
6069	5064	6864
6075	5074	6869
6084	5080	6883
6089	5082	6892
6102	5087	6909
6110	5086	6920
6121	5098	6928
6128	5107	6938
6130	5108	6951
6142	5115	6964
6155	5118	6971
6161	5123	6979
6162	5127	6990
6182	5130	7006
6184	5142	7018
6194	5145	7032
6199	5150	7032
6202	5159	7049
6208	5162	7063
6218	5166	7069
6226	5171	7085

6239	5176	7093
6250	5179	7098
6256	5182	7119
6263	5192	7127
6271	5200	7134
6281	5201	7152
6292	5209	7150
6300	5219	7174
6308	5222	7177
6307	5224	7187
6325	5225	7200
6330	5230	7214
6333	5237	7224
6342	5240	7236
6347	5253	7254
6359	5257	7259
6368	5255	7266
6379	5261	7276
6378	5271	7281
6391	5270	7307
6406	5275	7311
6407	5281	7317
6408	5290	7333
6425	5292	7338
6435	5295	7350
6435	5300	7369
6449	5311	7372
6445	5311	7380
6460	5310	7401
6473	5325	7407
6479	5325	7416
6491	5333	7430
6491	5342	7439
6507	5338	7457
6504	5339	7461
6525	5355	7473
6525	5357	7483
6528	5364	7492
6538	5365	7504
6548	5370	7510
6553	5373	7519
6563	5380	7539
6573	5387	7538
6578	5394	7558
6581	5397	7565
6589	5406	7582
6608	5411	7589
6608	5416	7599

6623	5418	7606
6626	5426	7618
6637	5428	7628
6646	5437	7639
6650	5440	7648
6658	5440	7661
6664	5442	7673
6675	5447	7686
6680	5452	7694
6689	5464	7707
6696	5465	7715
6706	5463	7716
6721	5476	7735
6725	5481	7744
6731	5480	7754
6738	5491	7762
6747	5497	7776
6748	5495	7788
6766	5501	7797
6762	5505	7809
6780	5507	7817
6780	5517	7829
6788	5527	7842
6801	5528	7849
6806	5532	7860
6814	5539	7859
6822	5543	7883
6836	5542	7894
6839	5551	7897
6851	5547	7907
6856	5557	7923
6866	5561	7928
6870	5571	7942
6879	5574	7950
6885	5581	7956
6890	5581	7975
6898	5583	7981
6906	5589	8000
6917	5600	7999
6920	5601	8013
6936	5603	8018
6941	5610	8029
6954	5619	8039
6957	5621	8054
6966	5623	8063
6965	5626	8077
6981	5627	8090
6986	5636	8095

6997	5644	8105
7006	5647	8115
7014	5658	8124
7024	5652	8133
7023	5657	8145
7038	5668	8153
7039	5672	8163
7051	5676	8177
7053	5681	8189
7057	5687	8194
7061	5686	8205
7071	5698	8221
7084	5700	8229
7097	5702	8230
7099	5706	8238
7110	5714	8256
7118	5711	8256
7123	5721	8272
7124	5724	8283
7142	5726	8300
7146	5731	8301
7153	5739	8321
7167	5742	8329
7164	5745	8343
7186	5756	8348
7192	5750	8354
7192	5756	8363
7199	5765	8377
7211	5770	8394
7219	5777	8399
7237	5777	8400
7230	5787	8418
7230	5781	8421
7243	5795	8431
7256	5799	8440
7258	5793	8448
7269	5804	8464
7273	5812	8475
7289	5817	8479
7295	5815	8484
7300	5824	8500
7307	5829	8511
7321	5828	8521
7328	5838	8528
7333	5834	8538
7347	5840	8546
7348	5844	8554
7358	5855	8567

7373	5857	8582
7376	5861	8587
7371	5868	8598
7390	5875	8602
7396	5881	8613
7389	5879	8625
7404	5880	8632
7413	5886	8641
7420	5888	8649
7426	5895	8666
7442	5905	8673
7450	5897	8679
7450	5911	8694
7458	5912	8700
7460	5919	8716
7477	5913	8718
7482	5929	8727
7497	5925	8736
7502	5931	8746
7505	5940	8753
7508	5940	8761
7518	5939	8774
7528	5949	8783
7528	5951	8790
7537	5954	8800
7556	5967	8808
7560	5967	8816
7560	5971	8832
7576	5977	8844
7576	5981	8841
7586	5981	8864
7596	5984	8871
7597	5990	8878
7609	5994	8881
7624	5997	8904
7625	6011	8897
7630	6013	8915
7641	6016	8922
7644	6014	8926
7656	6017	8945
7659	6026	8950
7679	6031	8957
7673	6040	8973
7686	6034	8977
7690	6040	8987
7701	6051	9005
7701	6048	9000
7716	6049	9014

7720	6057	9022
7730	6063	9032
7732	6067	9044
7744	6072	9055
7750	6072	9061
7763	6076	9067
7760	6072	9075
7778	6090	9093
7784	6091	9092
7789	6102	9102
7793	6095	9116
7805	6100	9126
7811	6102	9127
7816	6107	9138
7831	6119	9150
7845	6119	9154
7853	6122	9174
7844	6122	9185
7861	6131	9190
7858	6136	9188
7869	6136	9198
7872	6144	9201
7881	6138	9215
7890	6154	9228
7901	6156	9235
7908	6153	9245
7917	6164	9261
7929	6160	9263
7927	6168	9275
7935	6166	9278
7943	6169	9285
7960	6179	9289
7961	6181	9305
7964	6188	9300
7978	6199	9318
7989	6196	9336
7998	6197	9340
7999	6206	9349
8007	6207	9357
8010	6215	9366
8025	6214	9378
8026	6223	9385
8034	6221	9391
8043	6223	9405
8050	6231	9407
8057	6231	9419
8066	6236	9432
8070	6242	9440

8087	6243	9442
8086	6252	9450
8094	6249	9457
8105	6252	9462
8108	6267	9477
8120	6259	9479
8126	6271	9482
8136	6268	9503
8137	6274	9516
8149	6284	9517
8148	6285	9532
8163	6282	9530
8169	6280	9541
8175	6289	9555
8182	6296	9555
8182	6300	9565
8195	6308	9579
8200	6302	9580
8213	6306	9594
8216	6316	9586
8224	6318	9611
8230	6319	9623
8241	6327	9628
8240	6330	9629
8250	6334	9636
8263	6336	9642
8263	6342	9654
8271	6343	9665
8286	6344	9683
8288	6354	9684
8301	6354	9683
8314	6359	9698
8304	6365	9702
8312	6364	9712
8330	6360	9723
8339	6373	9729
8346	6379	9739
8349	6383	9748
8360	6381	9760
8364	6395	9763
8381	6393	9775
8386	6395	9780
8385	6402	9785
8391	6400	9794
8398	6407	9811
8404	6417	9819
8421	6418	9820
8424	6420	9833

8425	6420	9830
8433	6421	9852
8439	6423	9853
8454	6427	9860

Dnase	Rnase	Buffer
2708	31212	2286
2989	31344	2301
3266	31412	2308
3531	31456	2312
3790	31490	2321
4055	31505	2328
4314	31519	2332
4556	31521	2339
4808	31537	2346
5053	31538	2343
5287	31562	2353
5529	31558	2353
5757	31572	2358
5983	31563	2360
6213	31562	2367
6445	31555	2368
6658	31539	2374
6876	31557	2376
7088	31530	2383
7304	31551	2383
7512	31549	2387
7733	31533	2386
7935	31537	2387
8135	31535	2393
8330	31503	2396
8529	31513	2396
8721	31484	2404
8929	31497	2405
9115	31487	2407
9300	31472	2413
9489	31477	2415
9672	31452	2420
9854	31459	2415
10034	31450	2423
10220	31439	2423
10383	31439	2424
10560	31417	2427
10741	31410	2430
10912	31407	2435
11077	31385	2438
11239	31399	2441
11410	31395	2443
11579	31390	2442
11735	31384	2449
11895	31364	2451

12060	31359	2455
12207	31346	2450
12377	31343	2452
12520	31342	2461
12682	31330	2462
12822	31328	2465
12975	31318	2469
13119	31318	2469
13271	31291	2468
13418	31309	2476
13548	31271	2481
13696	31272	2482
13838	31262	2482
13979	31250	2485
14110	31247	2486
14244	31244	2491
14381	31208	2493
14517	31204	2491
14656	31224	2492
14787	31208	2498
14910	31206	2499
15036	31188	2498
15168	31179	2505
15289	31177	2510
15419	31183	2512
15554	31172	2512
15675	31163	2515
15797	31143	2512
15906	31137	2523
16025	31144	2520
16148	31120	2522
16266	31110	2524
16377	31080	2525
16476	31108	2527
16599	31100	2532
16718	31073	2530
16821	31066	2535
16943	31064	2539
17052	31064	2540
17156	31056	2541
17254	31037	2543
17371	31019	2545
17464	31022	2545
17566	31020	2549
17682	31004	2550
17780	30985	2552
17883	31010	2557
17985	30984	2557

18075	30972	2562
18177	30962	2563
18267	30958	2564
18365	30953	2565
18464	30944	2570
18553	30951	2569
18650	30926	2573
18725	30923	2573
18828	30923	2577
18917	30913	2581
19014	30908	2576
19105	30888	2578
19183	30874	2580
19272	30869	2586
19368	30874	2588
19455	30860	2592
19539	30860	2593
19617	30841	2591
19698	30851	2600
19764	30836	2596
19865	30837	2598
19956	30821	2598
20033	30811	2602
20105	30813	2606
20198	30799	2607
20251	30776	2605
20338	30791	2616
20412	30766	2616
20492	30783	2610
20564	30782	2619
20641	30751	2616
20708	30755	2620
20794	30751	2621
20862	30741	2625
20926	30713	2628
21006	30710	2632
21079	30721	2629
21145	30701	2633
21216	30701	2634
21282	30682	2639
21342	30690	2638
21415	30660	2641
21485	30690	2644
21553	30654	2646
21608	30652	2646
21676	30665	2649
21748	30643	2645
21799	30651	2653

21859	30621	2650
21928	30644	2654
21998	30617	2656
22062	30626	2660
22115	30619	2659
22171	30614	2662
22238	30610	2662
22312	30594	2668
22348	30611	2665
22405	30586	2669
22473	30586	2670
22532	30569	2675
22592	30576	2675
22638	30558	2674
22707	30564	2681
22751	30553	2682
22805	30549	2685
22856	30537	2684
22908	30546	2689
22964	30536	2687
23038	30523	2690
23083	30531	2692
23131	30521	2695
23197	30524	2699
23236	30506	2698
23283	30508	2704
23338	30508	2706
23392	30481	2702
23443	30502	2707
23492	30481	2709
23540	30474	2714
23593	30467	2709
23632	30484	2714
23674	30475	2721
23739	30452	2711
23781	30465	2719
23819	30470	2720
23867	30442	2717
23910	30424	2720
23963	30442	2728
23998	30399	2730
24054	30423	2732
24092	30414	2731
24134	30410	2733
24189	30401	2735
24225	30411	2732
24261	30402	2737
24302	30384	2740

24360	30376	2741
24395	30362	2743
24428	30390	2745
24474	30373	2747
24505	30367	2751
24543	30384	2755
24588	30354	2751
24633	30364	2755
24670	30343	2751
24703	30337	2756
24741	30321	2763
24774	30327	2763
24802	30310	2765
24862	30314	2765
24883	30310	2771
24905	30309	2765
24959	30312	2772
24980	30280	2771
25031	30288	2771
25069	30295	2775
25095	30272	2773
25123	30253	2777
25157	30263	2777
25195	30261	2781
25214	30237	2780
25262	30267	2785
25269	30235	2784
25325	30227	2790
25355	30225	2790
25371	30231	2790
25416	30223	2793
25440	30212	2790
25467	30194	2797
25495	30175	2794
25518	30205	2800
25564	30180	2801
25585	30171	2800
25621	30150	2801
25639	30182	2803
25675	30153	2802
25681	30116	2807
25727	30134	2808
25744	30124	2810
25776	30132	2813
25813	30121	2809
25837	30111	2816
25857	30111	2819
25884	30110	2819

25912	30083	2818
25926	30087	2819
25963	30077	2828
25987	30086	2826
26006	30058	2826
26040	30075	2831
26053	30064	2826
26081	30026	2834
26110	30055	2834
26142	30029	2836
26163	30012	2833
26180	30035	2839
26195	30030	2841
26220	30001	2845
26244	30021	2842
26275	29998	2842
26297	29989	2844
26314	29989	2847
26334	29968	2847
26368	29968	2849
26371	29961	2852
26389	29965	2851
26425	29955	2856
26436	29964	2856
26456	29934	2853
26486	29928	2859
26493	29923	2859
26509	29923	2861
26547	29902	2865
26556	29919	2863
26573	29888	2863
26578	29902	2867
26606	29872	2865
26645	29872	2870
26652	29873	2868
26663	29878	2869
26690	29869	2872
26706	29845	2873
26720	29842	2877
26742	29846	2876
26766	29856	2880
26786	29819	2881
26809	29843	2885
26811	29807	2882
26824	29806	2883
26839	29819	2884
26865	29813	2889
26875	29797	2893

26900	29790	2893
26897	29781	2893
26929	29787	2891
26940	29776	2896
26944	29768	2893
26985	29758	2894
26986	29754	2898
26988	29748	2900
27018	29755	2902
27036	29737	2906
27049	29732	2906
27061	29714	2906
27077	29713	2909
27082	29706	2908
27093	29710	2907
27145	29702	2914
27133	29697	2912
27150	29686	2913
27163	29664	2916
27164	29668	2915
27194	29657	2918
27202	29656	2918
27206	29656	2922
27227	29658	2924
27231	29642	2922
27248	29637	2929
27263	29640	2926
27279	29644	2928
27281	29640	2931
27302	29632	2930
27305	29600	2931
27310	29609	2932
27338	29602	2933
27345	29583	2935
27365	29604	2938
27359	29567	2941
27374	29580	2939
27379	29553	2938
27395	29544	2941
27426	29542	2942
27419	29547	2947
27433	29537	2949
27450	29519	2947
27443	29529	2949
27457	29489	2950
27479	29508	2951
27476	29505	2957
27502	29516	2952

27500	29495	2954
27530	29491	2956
27526	29483	2959
27516	29475	2962
27546	29481	2964
27561	29452	2965
27563	29473	2959
27561	29441	2964
27586	29448	2966
27586	29431	2970
27581	29413	2965
27600	29408	2972
27616	29402	2970
27614	29394	2972
27634	29402	2965
27631	29361	2974
27637	29391	2977
27635	29394	2979
27656	29379	2972
27670	29364	2985
27674	29352	2980
27679	29338	2979
27691	29339	2981
27691	29337	2983
27694	29319	2986
27700	29308	2983
27713	29309	2987
27720	29293	2989
27718	29301	2990
27734	29280	2989
27730	29281	2988
27735	29279	2997
27763	29281	2994
27734	29273	2997
27759	29254	2999
27771	29245	3000
27779	29254	2995
27789	29235	2997
27783	29236	2997
27775	29218	3000
27792	29209	3005
27797	29219	3004
27812	29218	3004
27820	29205	3005
27826	29194	3001
27811	29208	3000
27841	29175	3008
27855	29180	3006

27845	29185	3011
27851	29169	3014
27842	29165	3014
27866	29157	3014
27850	29140	3015
27867	29139	3020
27870	29145	3016
27875	29126	3013
27881	29136	3020
27879	29112	3022
27893	29129	3022
27894	29124	3026
27904	29095	3024
27910	29090	3026
27905	29077	3027
27921	29076	3029
27905	29078	3036
27924	29079	3026
27910	29060	3031
27923	29057	3035
27930	29055	3033
27909	29055	3037
27950	29024	3038
27943	29041	3039
27944	29012	3039
27958	29015	3042
27948	29008	3039
27955	29001	3041
27957	28981	3042
27955	28985	3041
27964	28972	3047
27973	28977	3043
27970	28958	3045
27966	28965	3051
27978	28958	3053
27966	28942	3047
27970	28928	3048
27969	28913	3050
27986	28920	3057
27982	28907	3055
27985	28921	3052
27979	28901	3054
27990	28895	3061
27986	28893	3062
27998	28879	3061
28001	28875	3060
27999	28879	3061
28000	28878	3061

27999	28859	3068
28012	28847	3067
28002	28860	3067
28019	28834	3074
28011	28839	3066
28035	28833	3070
28024	28816	3070
28044	28806	3068
28022	28815	3078
28018	28806	3074
28018	28792	3073
28039	28794	3075
28040	28776	3072
28040	28794	3077
28042	28779	3076
28049	28763	3077
28027	28754	3084
28038	28764	3079
28039	28765	3084
28040	28753	3083
28032	28730	3085
28040	28736	3088
28056	28718	3090
28054	28721	3085
28045	28707	3087
28052	28703	3097
28043	28693	3091
28052	28694	3092
28049	28708	3093
28053	28678	3097
28060	28674	3095
28046	28649	3094
28052	28648	3100
28059	28671	3100
28055	28646	3101
28043	28643	3103
28043	28649	3102
28056	28613	3102
28054	28622	3106
28056	28606	3105
28065	28601	3107
28073	28602	3108
28053	28601	3105
28077	28592	3103
28047	28582	3107
28073	28591	3108
28061	28577	3109
28063	28585	3111

28059	28575	3113
28062	28566	3114
28067	28540	3116
28049	28530	3122
28058	28534	3121
28078	28540	3115
28071	28534	3116
28062	28508	3125
28073	28535	3122
28058	28502	3121
28057	28501	3119
28063	28507	3123
28048	28500	3124
28073	28489	3122
28078	28471	3127
28083	28472	3129
28074	28469	3130
28053	28460	3131
28056	28437	3126
28064	28441	3132
28064	28444	3130
28063	28437	3133
28066	28437	3133
28052	28404	3137
28048	28421	3136
28059	28420	3139
28066	28394	3142
28060	28391	3137
28050	28392	3142
28060	28406	3140
28037	28370	3144
28060	28365	3148
28041	28383	3150
28062	28361	3146
28056	28361	3146
28052	28354	3146
28059	28338	3150
28067	28363	3148
28037	28344	3148
28032	28332	3152
28036	28323	3151
28060	28320	3157
28044	28307	3153
28042	28306	3153
28020	28287	3155
28027	28292	3156
28030	28288	3158
28036	28294	3157

28028	28261	3165
28033	28271	3162
28030	28259	3166
28032	28255	3159
28032	28248	3166
28033	28251	3160
28018	28236	3165
28019	28231	3166
28031	28247	3173
28020	28233	3173
28016	28215	3171
28027	28223	3166
28013	28220	3170
27993	28203	3171
28006	28185	3176
27990	28178	3172
28006	28188	3176
28008	28168	3176
28006	28182	3179
28000	28155	3174
28009	28166	3177
28001	28151	3180
27990	28167	3179
28006	28150	3177
27958	28126	3183
27991	28125	3187
27991	28115	3182
27973	28114	3181
27997	28115	3186
27980	28099	3185
27985	28119	3190
28003	28087	3185
27977	28089	3189
27980	28090	3189
27983	28088	3191
27970	28080	3191
27968	28064	3193
27977	28057	3195
27965	28043	3191
27978	28046	3196
27968	28028	3196
27977	28031	3199
27942	28029	3198
27943	28021	3196
27949	28007	3199
27951	28019	3198
27966	27991	3198
27937	28004	3201

27964	27992	3198
27946	27987	3203
27936	27967	3202
27932	27975	3205
27916	27962	3204
27944	27959	3200
27921	27963	3211
27934	27955	3208
27928	27943	3203
27917	27942	3212
27923	27933	3204
27919	27942	3210
27918	27903	3210
27912	27891	3211
27908	27902	3208
27897	27905	3214
27892	27903	3224
27905	27890	3208
27882	27887	3219
27899	27874	3220
27887	27869	3218
27881	27876	3215
27894	27855	3220
27880	27838	3220
27869	27861	3220
27871	27829	3222
27879	27855	3227
27877	27824	3228
27870	27840	3224
27860	27816	3227
27867	27816	3224
27859	27829	3231
27857	27815	3227
27850	27806	3227
27840	27779	3230
27836	27786	3231
27839	27788	3231
27857	27776	3235
27856	27770	3225
27825	27759	3233
27821	27760	3230
27819	27769	3232
27828	27735	3232
27837	27758	3238
27808	27739	3240
27827	27721	3238
27815	27716	3240
27827	27713	3241

27810	27717	3244
27797	27695	3239
27798	27700	3243
27809	27698	3244
27794	27697	3245
27806	27683	3244
27806	27674	3242
27800	27670	3246
27792	27662	3249
27782	27667	3248
27774	27641	3249
27784	27632	3250
27774	27650	3252
27761	27643	3246
27771	27624	3253
27767	27644	3253
27770	27625	3252
27749	27630	3254
27751	27611	3255
27749	27589	3251
27751	27595	3257
27741	27594	3256
27734	27575	3259
27739	27593	3255
27736	27570	3262
27748	27572	3261
27729	27584	3259
27731	27573	3263
27708	27548	3264
27726	27539	3264
27720	27525	3264
27703	27537	3265
27718	27519	3266
27709	27537	3269
27711	27510	3263
27693	27507	3266
27687	27513	3264
27697	27498	3268
27691	27500	3274
27693	27496	3269
27678	27480	3271
27680	27485	3273
27678	27469	3272
27669	27479	3271
27663	27469	3272
27653	27455	3275
27657	27441	3279
27652	27442	3278

27640	27447	3277
27653	27430	3276
27640	27425	3280
27632	27415	3281
27643	27401	3277
27652	27394	3283
27627	27403	3277
27641	27384	3284
27613	27386	3290
27618	27376	3282
27611	27370	3279
27593	27375	3289
27617	27369	3286
27619	27378	3286
27588	27369	3285
27599	27350	3284
27608	27342	3287
27582	27357	3286
27569	27336	3283
27608	27334	3288
27569	27318	3292
27573	27324	3291
27588	27315	3295
27573	27301	3297
27566	27306	3299
27562	27304	3294
27565	27291	3291
27554	27294	3291
27549	27260	3291
27554	27269	3302
27551	27266	3297
27551	27257	3301
27535	27245	3300
27534	27244	3298
27523	27258	3299
27538	27235	3300
27526	27224	3306
27523	27232	3301
27507	27221	3298
27491	27228	3299
27508	27221	3305
27498	27194	3306
27512	27199	3306
27503	27186	3304
27492	27191	3308
27497	27176	3308
27464	27169	3305
27460	27167	3307

27470	27180	3312
27469	27153	3310
27465	27156	3310
27463	27148	3313

Figure S2A FloPol (deoxy vs ribo)

deoxy	390	350.5	310	259	192.5
STDEV	0	9.19238816	1.41421356	12.7279221	20.5060967
ribo	342	307	268.5	220	150
STDEV	1.41421356	2.82842713	6.36396103	2.82842713	1.41421356
[prot] (nM)	7250	3625	1812.5	906.25	453.125

126.5	95.5	90	87	87.5	88	87
13.4350288	4.94974747	1.41421356	4.24264069	3.53553391	1.41421356	2.82842713
107	93.5	92	89.5	92	91.5	91.5
1.41421356	0.70710678	0	0.70710678	2.82842713	2.12132034	3.53553391
226.563	113.281	56.641	28.32	14.16	7.08	3.54

88.5	86.5	86.5	86.5	86.5	86	86
0.70710678	2.12132034	0.70710678	0.70710678	0.70710678	4.24264069	1.41421356
91	90	89	91	92	90	89.5
0	0	1.41421356	1.41421356	1.41421356	1.41421356	0.70710678
1.77	0.885	0.443	0.221	0.111	0.055	0.028

85.5
2.12132034
91.5
2.12132034
0.014

Figure S2B length dependence

TGTTCA	390	350.5	310	259	192.5
STDEV	0	9.19238816	1.41421356	12.7279221	20.5060967
[prot] (nM)	7250	3625	1812.5	906.25	453.125
TGTTCA-ssDNA15	326.5	282.5	247	199.5	154.5
STDEV	0.70710678	0.70710678	7.07106781	0.70710678	2.12132034
TGTTCA-ssDNA32	359.5	329	293.5	248	191
STDEV	0.70710678	1.41421356	2.12132034	1.41421356	1.41421356
[prot] (nM)	7270	3635	1817.5	908.75	454.375
TGTTCA-ssDNA57	390.5	381.5	367.5	341.5	299
STDEV	0.70710678	0.70710678	0.70710678	0.70710678	1.41421356
[prot] (nM)	26150	13075	6537.5	3268.75	1634.375
ssDNA57	327	263	210.5	170.5	148
STDEV	1.41421356	2.82842713	2.12132034	0.70710678	2.82842713
[prot] (nM)	26150	13075	6537.5	3268.75	1634.375

126.5	95.5	90	87	87.5	88	87
13.4350288	4.94974747	1.41421356	4.24264069	3.53553391	1.41421356	2.82842712
226.563	113.281	56.641	28.32	14.16	7.08	3.54
124	108.5	101.5	96.5	96	94.5	93.5
4.24264069	4.94974747	3.53553391	2.12132034	4.24264069	3.53553391	2.12132034
151	129	118	111.5	110	108.5	104
1.41421356	1.41421356	1.41421356	0.70710678	1.41421356	3.53553391	0
227.1875	113.59375	56.796875	28.3984375	14.1992188	7.09960938	3.54980469
240	177.5	134.5	116	111	104.5	103.5
1.41421356	0.70710678	2.12132034	0	0	2.12132034	0.70710678
817.1875	408.59375	204.296875	102.148438	51.0742188	25.5371094	12.7685547
133	126	123	119.5	129	122	119
1.41421356	1.41421356	0	0.70710678	8.48528137	0	0
817.1875	408.59375	204.296875	102.148438	51.0742188	25.5371094	12.7685547

88.5	86.5	86.5	86.5	86.5	86	86
0.70710678	2.12132034	0.70710678	0.70710678	0.70710678	4.24264069	1.41421356
1.77	0.885	0.443	0.221	0.111	0.055	0.028
91.5	93	89.5	91.5	91	89	90
2.12132034	2.82842713	0.70710678	2.12132034	0	0	1.41421356
104.5	104.5	105	103	103.5	103.5	103.5
0.70710678	3.53553391	1.41421356	0	2.12132034	0.70710678	0.70710678
1.77490234	0.88745117	0.44372559	0.22186279	0.1109314	0.0554657	0.02773285
101.5	101.5	100	99.5	101.5	100	99.5
0.70710678	2.12132034	1.41421356	3.53553391	2.12132034	0	2.12132034
6.38427734	3.19213867	1.59606934	0.79803467	0.39901733	0.19950867	0.09975433
120	121.5	120.5	130.5	122	119.5	120.5
1.41421356	0.70710678	0.70710678	16.263456	1.41421356	0.70710678	0.70710678
6.38427734	3.19213867	1.59606934	0.79803467	0.39901733	0.19950867	0.09975433

85.5
2.12132034
0.014
91
1.41421356
104
0
0.01386643
101
0
0.04987717
121.5
0.70710678
0.04987717

Figure S2C FloPol (TGTTCA salt dependence)

50 mM KCl	FPS006	364	354	334	298	250.5
		1.41421356	0	0	2.82842712	3.53553391
	FPS006_ssDNA57	396	394.5	390	380.5	371
		1.41421356	0.70710678	2.82842712	0.70710678	1.41421356
	ssDNA57	361	358.5	354	340	288
		1.41421356	0.70710678	0	1.41421356	4.24264069

150 mM KCl	FPS006	342	303.5	260	212.5	162.5
		0	2.12132034	1.41421356	3.53553391	2.12132034
	FPS006_ssDNA57	385.5	368.5	342	304	257
		3.53553391	2.12132034	1.41421356	1.41421356	5.65685425
	ssDNA57	319.5	230	170.5	132	110.5
		0.70710678	8.48528137	9.19238816	0	2.12132034
	[WT] (nM)	18000	9000	4500	2250	1125

197.5	141	104.5	89	84.5	80	79
4.94974747	2.82842712	4.94974747	4.24264069	0.70710678	2.82842712	0

321.5	233	165.5	127	106.5	95.5	89.5
10.6066017	12.7279221	9.19238816	2.82842712	0.70710678	0.70710678	0.70710678
201.5	141	109.5	97	88.5	87	83.5
7.77817459	2.82842712	0.70710678	1.41421356	0.70710678	0	0.70710678

121	99.5	87.5	84	83.5	83.5	83.5
1.41421356	2.12132034	2.12132034	2.82842712	2.12132034	0.70710678	0.70710678

198.5	147	114	99	95	91	89
4.94974747	8.48528137	2.82842712	4.24264069	1.41421356	2.82842712	1.41421356
101.5	95.5	91.5	90.5	90	91	90
0.70710678	0.70710678	0.70710678	2.12132034	2.82842712	1.41421356	2.82842712
562.5	281.25	140.625	70.3125	35.15625	17.578125	8.7890625

79	80.5	77.5	78	80	78.5	77
0	0.70710678	0.70710678	1.41421356	1.41421356	0.70710678	0
85	85.5	84.5	82	84.5	83.5	82
0	0.70710678	0.70710678	1.41421356	2.12132034	0.70710678	0
83	82	82.5	82	83	81.5	81
1.41421356	0	2.12132034	1.41421356	1.41421356	2.12132034	0
83	80.5	80.5	81.5	81	83	80.5
2.82842712	0.70710678	0.70710678	0.70710678	0	1.41421356	3.53553391
87	88	86	87	86.5	87.5	86.5
0	1.41421356	0	1.41421356	0.70710678	2.12132034	2.12132034
90.5	90.5	89	91.5	91	89	89
0.70710678	3.53553391	4.24264069	0.70710678	1.41421356	0	1.41421356
4.39453125	2.19726563	1.09863281	0.54931641	0.2746582	0.1373291	0.06866455

79
1.41421356

84.5
2.12132034
84
1.41421356

88
11.3137085

86
1.41421356
88.5
0.70710678
0.03433228

Figure S2C FloPol Kinetic time points

0 hr	FPS006	364	354	334	298	250.5
		1.41421356	0	0	2.82842712	3.53553391
	FPS006_ssDNA57	396	394.5	390	380.5	371
		1.41421356	0.70710678	2.82842712	0.70710678	1.41421356
	ssDNA57	361	358.5	354	340	288
		1.41421356	0.70710678	0	1.41421356	4.24264069
4 hr	FPS006	358.5	343.5	315.5	278	228
		2.12132034	0.70710678	3.53553391	2.82842712	7.07106781
	FPS006_ssDNA57	393	388	383.5	374	366
		1.41421356	0	2.12132034	0	1.41421356
	ssDNA57	357	355	350	342	293.5
		0	0	1.41421356	1.41421356	7.77817459
12 hr	FPS006	378	302	221	157	119
		7.07106781	4.24264069	8.48528137	7.07106781	7.07106781
	FPS006_ssDNA57	427	403	378.5	342	307.5
		2.82842712	4.24264069	6.36396103	4.24264069	4.94974747
	ssDNA57	363	303.5	227.5	176	148.5
		2.82842712	0.70710678	4.94974747	4.24264069	3.53553391

197.5	141	104.5	89	84.5	80	79
4.94974747	2.82842712	4.94974747	4.24264069	0.70710678	2.82842712	0
321.5	233	165.5	127	106.5	95.5	89.5
10.6066017	12.7279221	9.19238816	2.82842712	0.70710678	0.70710678	0.70710678
201.5	141	109.5	97	88.5	87	83.5
7.77817459	2.82842712	0.70710678	1.41421356	0.70710678	0	0.70710678
173.5	119	90	79	75.5	73.5	75
4.94974747	5.65685425	4.24264069	4.24264069	0.70710678	3.53553391	0
328	257.5	182	128.5	102	92.5	84.5
9.89949494	12.0208153	11.3137085	3.53553391	1.41421356	3.53553391	0.70710678
210	141.5	106	91.5	79.5	78.5	80
9.89949494	3.53553391	2.82842712	0.70710678	0.70710678	0.70710678	1.41421356
102.5	96	96	95	97.5	96	98
4.94974747	7.07106781	9.89949494	5.65685425	6.36396103	7.07106781	5.65685425
265	218	171	139	128.5	124	120.5
8.48528137	9.89949494	8.48528137	4.24264069	0.70710678	1.41421356	2.12132034
137	131	125	126	127	132.5	130.5
2.82842712	0	1.41421356	1.41421356	2.82842712	3.53553391	2.12132034

79	80.5	77.5	78	80	78.5	77
0	0.70710678	0.70710678	1.41421356	1.41421356	0.70710678	0
85	85.5	84.5	82	84.5	83.5	82
0	0.70710678	0.70710678	1.41421356	2.12132034	0.70710678	0
83	82	82.5	82	83	81.5	81
1.41421356	0	2.12132034	1.41421356	1.41421356	2.12132034	0
74.5	76	75.5	74.5	76.5	79.5	76.5
2.12132034	1.41421356	4.94974747	2.12132034	2.12132034	0.70710678	0.70710678
83	83.5	82	78.5	79.5	81.5	78.5
1.41421356	0.70710678	2.82842712	0.70710678	3.53553391	0.70710678	2.12132034
83	79.5	81	79	81	79	80
4.24264069	0.70710678	1.41421356	0	0	1.41421356	2.82842712
102.5	102.5	104	104	104	106	104.5
4.94974747	6.36396103	7.07106781	8.48528137	4.24264069	7.07106781	2.12132034
120.5	119	118.5	116	117	114.5	116
3.53553391	2.82842712	0.70710678	1.41421356	2.82842712	0.70710678	0
134	133	131.5	131	134	131	130
4.24264069	0	0.70710678	0	0	1.41421356	1.41421356

79
1.41421356
84.5
2.12132034
84
1.41421356
74.5
0.70710678
81
0
82
0
105
0
116.5
0.70710678
133
1.41421356

Figure S3	Oligonucleotide binding and dNTPase activity	
	FPLC	%DNA-dNTP titration: taking 495 nm maximum<br>x1 = [0 0.1 0.25 0.5 1 2 5];<br>y1 = [0.078 1.334 2.482 4.115 6.236 9.14 9.
	dNTPase activity	%DNA-dNTP titration: taking 495 nm maximum<br>x3 = [0 0.1 0.25 0.5 1 2 5];<br>y3 = [100 66.62 61.37 41.69 28.50 21.27 22.

```
of the protein-bound oligo peak

22];
```


```
of the protein-bound oligo peak

22];
```

Fig Supplementary 5BC
dNTP depletion

dATP	pLVX	WT#1	WT#2	WT#3
	2.18	0.04	0.02	0.04
	1.95	0.04	0.03	0.06
	1.9	0.03	0.03	0.04
	1.89	0.04	0.03	0.03

dGTP	pLVX	WT#1	WT#2	WT#3
	1.2	0.03	0.02	0.03
	1.24	0.02	0.03	0.03
	1.18	0.03	0.03	0.03
	1.06	0.03	0.03	0.04

HIV restriction of mutants

	pLVX		WT#1	
500	12.2	12.2	1.9	1.85
250				
125	2.13	2.5	0.77	0.95
62.5				
32.25				
0	0	0	0	0

SEC titration curve of mutants

```
%line1 - D311A-F*DNA1 DNA-dNTP
x1 = [0 1 2 5 10];
y1 = [0 4.808 8.627 8.156 9.180];

%line2 - K523A-F*DNA1 DNA-dNTP
x2 = [0 1 2 5 10];
y2 = [0 4.2170 7.4430 13.0470 15.6490];

%line2 - R352A-F*DNA1 DNA-dNTP
x3 = [0 1 2 5 10];
y3 = [0 4.131 7.08 12.28 14.52];
```

WT#4	H376A#1	H376A#2	H376A#3	H376A#4
0.04	0.12	0.12	0.15	0.17
0.04	0.14	0.15	0.15	0.18
0.04	0.15	0.15	0.12	0.16
0.04	0.13	0.13	0.14	0.16

WT#4	H376A#1	H376A#2	H376A#3	H376A#4
0.02	0.14	0.14	0.1	0.17
0.01	0.12	0.14	0.13	0.18
0.02	0.14	0.13	0.13	0.15
0.02	0.14	0.13	0.16	0.16

WT#2		WT#3		WT#4		H376[illegible]
1.46	1.76	1.52	0.85	2.2	2.53	13.1
0.59	0.38	0.14	0.96	0.69	0.97	3.19
						2.06
						0.43
0	0	0	0	0	0	0

6A#1		H376A#2		H376A#3		H376A#4	
	12.7	7.89	8.15	8.58	8.42	10.6	8.81
	4.84	4.1	4	3.59	3.17	3.63	2.95
	2.61	1.66	1.22	2.14	1.12	1.39	1.2
	0.71	0.69	0.93	0.52	0.35	0.52	0.57
	0	0	0	0	0	0	0

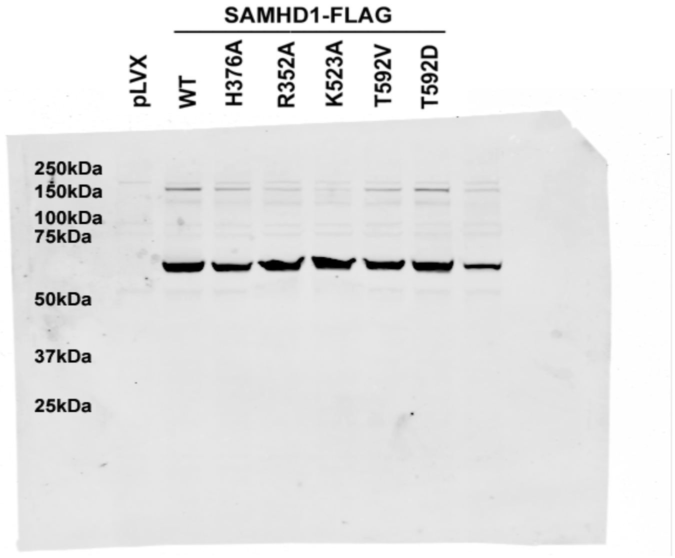
SAMHD1-FLAG
pLVX
WT
H376A
R352A
K523A
T592V
T592D
250kDa
150kDa
100kDa
75kDa
50kDa
37kDa
25kDa

anti-Flag

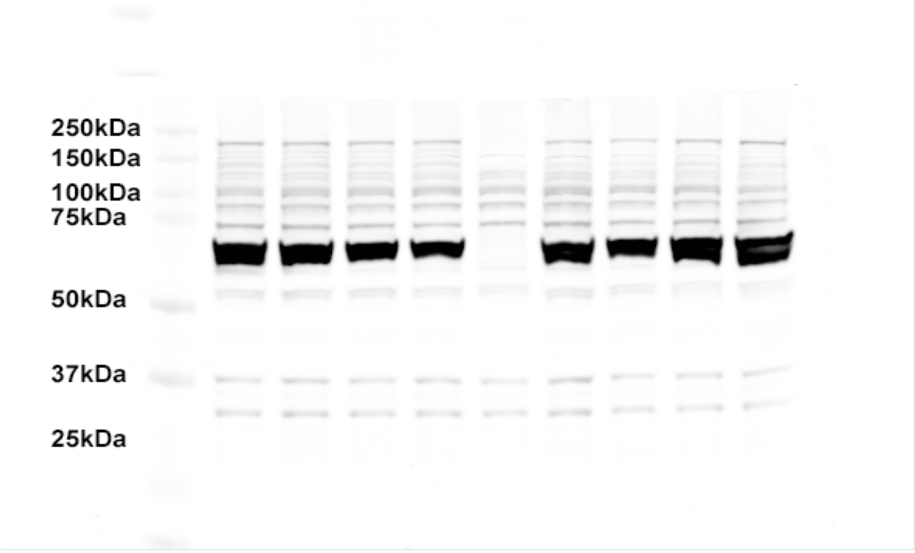
250kDa
150kDa
100kDa
75kDa
50kDa
37kDa
25kDa

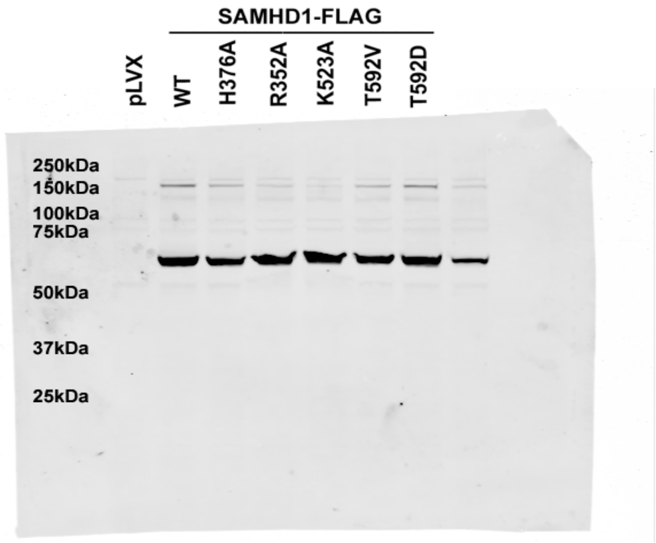
SAMHD1-FLAG
pLVX
WT
H376A
R352A
K523A
T592V
T592D
250kDa
150kDa
100kDa
75kDa
50kDa
37kDa
25kDa

anti-Flag

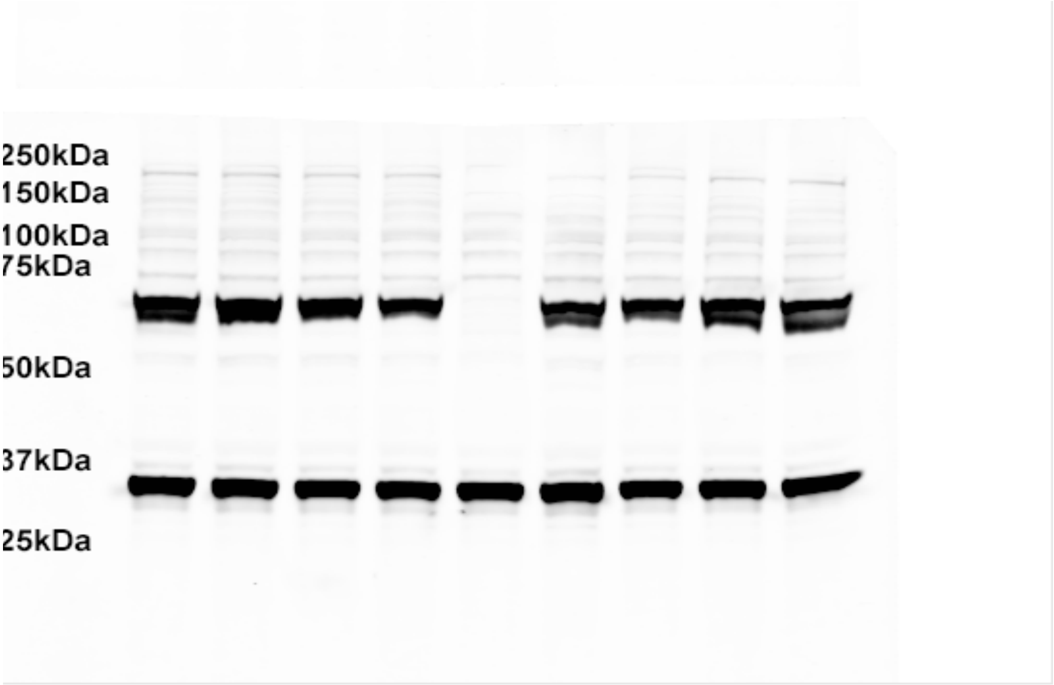
250kDa
150kDa
100kDa
75kDa
50kDa
37kDa
25kDa
